# Supplementary figures and images for: Accelerometer compared with questionnaire measures of physical activity in relation to body size and composition: a large cross-sectional analysis of UK Biobank
Source: BMJ Open. 2019 Jan 29;9(1):e024206. doi: 10.1136/bmjopen-2018-024206 (PMC6352868; doi:10.1136/bmjopen-2018-024206)

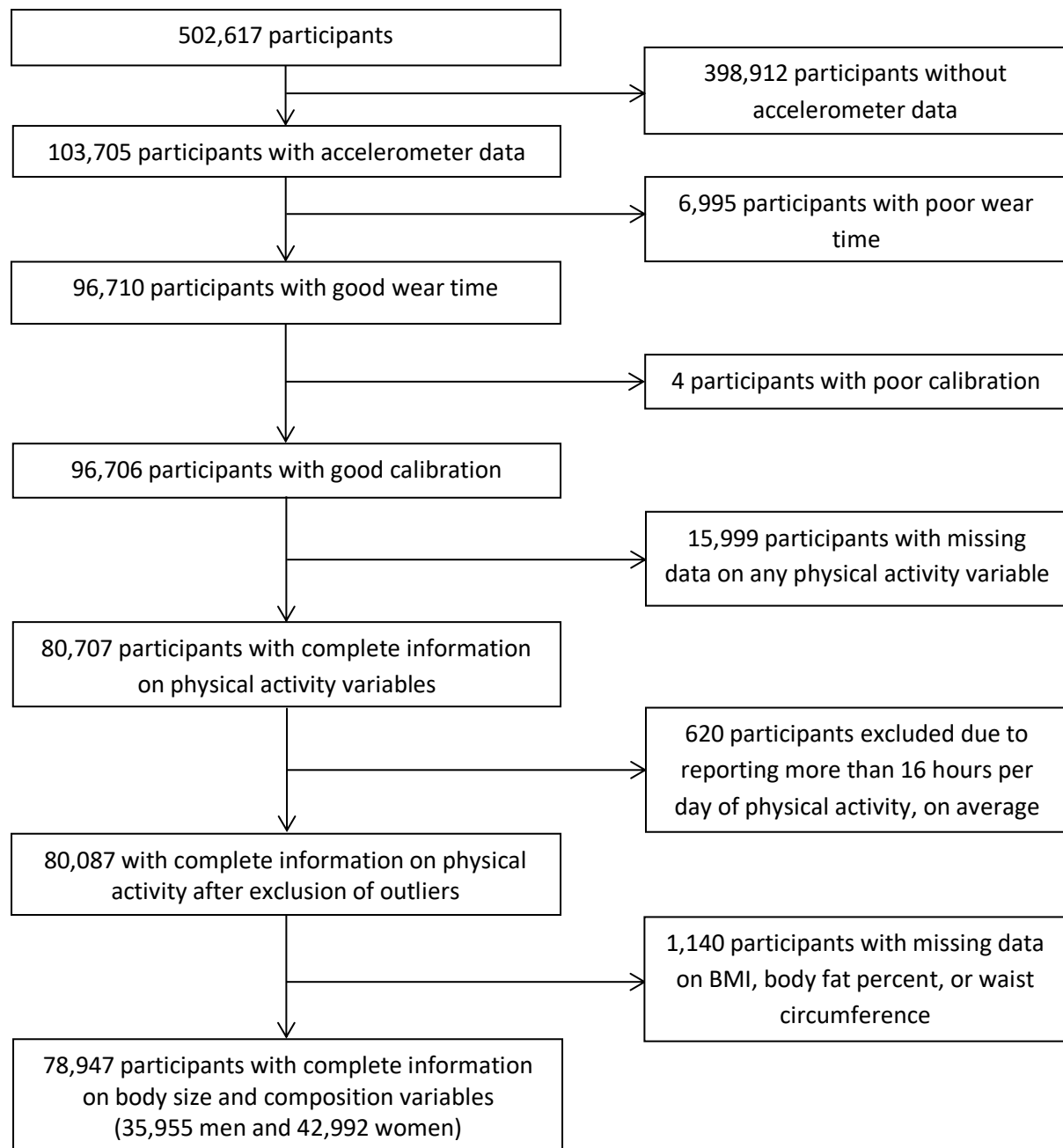

Supplement: Supplementary file 1 [file bmjopen-2018-024206supp001.pdf]
