# Supplementary material for: Accelerometer compared with questionnaire measures of physical activity in relation to body size and composition: a large cross-sectional analysis of UK Biobank
Source: BMJ Open. 2019 Jan 29;9(1):e024206. doi: 10.1136/bmjopen-2018-024206 (PMC6352868; doi:10.1136/bmjopen-2018-024206)
Supplement: Supplementary file 2 [file bmjopen-2018-024206supp002.pdf]

Supplementary Table 1. Physical activity questions from the UK Biobank baseline questionnaire

Question	Potential Responses
In a typical WEEK, on how many days did you walk for at least 10 minutes at a time? (Include walking that you do at work, travelling to and from work, and for sport or leisure)	Number of days, “Do not know”, “Unable to walk”, or “Prefer not to answer”
How many minutes did you usually spend walking on a typical DAY?	Quantity of minutes, “Do not know”, or “Prefer not to answer”
In a typical WEEK, on how many days did you do 10 minutes or more of moderate physical activities like carrying light loads, cycling at normal pace? (Do not include walking)	Number of days, “Do not know”, or “Prefer not to answer”
How many minutes did you usually spend doing moderate activities on a typical DAY?	Quantity of minutes, “Do not know”, or “Prefer not to answer”
In a typical WEEK, how many days did you do 10 minutes or more of vigorous physical activity? (These are activities that make you sweat or breathe hard such as fast cycling, aerobics, heavy lifting)	Number of days, “Do not know”, or “Prefer not to answer”
How many minutes did you usually spend doing vigorous activities on a typical DAY?	Quantity of minutes, “Do not know”, or “Prefer not to answer”
