# Supplementary material for: Accelerometer compared with questionnaire measures of physical activity in relation to body size and composition: a large cross-sectional analysis of UK Biobank
Source: BMJ Open. 2019 Jan 29;9(1):e024206. doi: 10.1136/bmjopen-2018-024206 (PMC6352868; doi:10.1136/bmjopen-2018-024206)
Supplement: Supplementary file 3 [file bmjopen-2018-024206supp003.pdf]

Supplementary Table 2. Spearman correlation between self-reported physical activity and accelerometer-measured physical activity, according to participant characteristics in UK Biobank men

	N Men (%)	Correlation	95% Confidence Interval
**Total**	35,955	0.24	0.23, 0.25
**Age group at recruitment (years)**			
<55 years	13,214 (36.8)	0.31	0.29, 0.32
55+ years	22,741 (63.3)	0.22	0.21, 0.23
**Socioeconomic status, fifths**			
Top fifth	7,584 (21.1)	0.23	0.21, 0.25
Bottom fifth	6,800 (18.9)	0.26	0.23, 0.28
**BMI (kg/m^2)**			
<25	10,590 (29.5)	0.27	0.25, 0.28
25-29.9	17,874 (49.7)	0.21	0.19, 0.22
>30	7,491 (20.8)	0.22	0.80, 0.24
**College or university degree**			
Yes	16,709 (46.5)	0.25	0.24, 0.27
No	19,246 (53.5)	0.24	0.22, 0.25
**Current employment status**			
In paid employment or self-employed	22,942 (63.8)	0.27	0.26, 0.28
Retired	11,361 (31.6)	0.24	0.22, 0.26
Other	1,652 (4.6)	0.30	0.26, 0.34
**Job involves mainly walking/standing**			
Never or rarely	9,825 (42.8)	0.29	0.27, 0.31
Sometimes	7,534 (32.9)	0.24	0.22, 0.26
Usually or Always	5,574 (24.3)	0.19	0.16, 0.21
**Job involves heavy manual work**			
Never, rarely	16,443 (71.7)	0.27	0.26, 0.29
Sometimes	4,160 (18.1)	0.17	0.14, 0.19
Usually or Always	2,335 (10.2)	0.12	0.08, 0.16
**Alcohol intake frequency**			
Weekly or more	29,421 (81.8)	0.23	0.22, 0.24
Less than weekly	6,530 (18.2)	0.28	0.26, 0.30
**Smoking status**			
Never	18,928 (52.6)	0.26	0.24, 0.27
Ever	16,964 (47.2)	0.22	0.21, 0.24
**Long-standing illness or disability**			
No	25,129 (69.9)	0.23	0.22, 0.24
Yes	10,825 (30.1)	0.25	0.23, 0.27
