# Supplementary material for: Accelerometer compared with questionnaire measures of physical activity in relation to body size and composition: a large cross-sectional analysis of UK Biobank
Source: BMJ Open. 2019 Jan 29;9(1):e024206. doi: 10.1136/bmjopen-2018-024206 (PMC6352868; doi:10.1136/bmjopen-2018-024206)
Supplement: Supplementary file 4 [file bmjopen-2018-024206supp004.pdf]

Supplementary Table 3. Spearman correlation between self-reported physical activity and accelerometer-measured physical activity, according to participant characteristics in UK Biobank women

	N Women (%)	Correlation	95% Confidence Interval
**Total**	42,992	0.22	0.21, 0.23
**Age group at recruitment (years)**			
<55 years	18,973 (44.1)	0.26	0.25, 0.28
55+ years	24,019 (55.9)	0.20	0.19, 0.22
**Socioeconomic status, fifths**			
Top fifth	8,401 (19.5)	0.22	0.30, 0.24
Bottom fifth	8,744 (20.3)	0.22	0.30, 0.24
**BMI (kg/m^2)**			
<25	20,255 (47.1)	0.21	0.20, 0.23
25-29.9	15,146 (35.2)	0.18	0.17, 0.20
>30	7,591 (17.7)	0.15	0.13, 0.17
**College or university degree**			
Yes	19,214 (44.7)	0.22	0.21, 0.24
No	23,778 (55.3)	0.22	0.20, 0.23
**Current employment status**			
In paid employment or self-employed	26,693 (62.1)	0.24	0.23, 0.25
Retired	12,710 (29.6)	0.22	0.20, 0.24
Other	3,589 (8.4)	0.30	0.27, 0.33
**Job involves mainly walking/standing**			
Never or rarely	12,191 (45.7)	0.25	0.23, 0.27
Sometimes	7,839 (29.4)	0.21	0.19, 0.23
Usually or Always	6,648 (24.9)	0.18	0.16, 0.20
**Job involves heavy manual work**			
Never, rarely	20,762 (77.8)	0.24	0.22, 0.25
Sometimes	4,353 (16.3)	0.17	0.14, 0.20
Usually or Always	1,567 (5.9)	0.13	0.08, 0.18
**Alcohol intake frequency**			
Weekly or more	29,829 (69.4)	0.22	0.21, 0.23
Less than weekly	13,152 (30.6)	0.21	0.20, 0.23
**Smoking status**			
Never	25,998 (60.5)	0.21	0.20, 0.22
Ever	16,936 (39.4)	0.23	0.22, 0.25
**Long-standing illness or disability**			
No	32,307 (75.2)	0.21	0.20, 0.22
Yes	10,685 (24.9)	0.23	0.21, 0.24
