# Supplementary material for: Accelerometer compared with questionnaire measures of physical activity in relation to body size and composition: a large cross-sectional analysis of UK Biobank
Source: BMJ Open. 2019 Jan 29;9(1):e024206. doi: 10.1136/bmjopen-2018-024206 (PMC6352868; doi:10.1136/bmjopen-2018-024206)
Supplement: Supplementary file 5 [file bmjopen-2018-024206supp005.pdf]

Supplementary Table 4. Association of self-reported and accelerometer-measured physical activity with measures of adiposity

	N	BMI (kg/m^2)	95% CI	Body fat percent	95% CI	Waist circumference (cm)	95% CI
**Men**							
**Questionnaire**							
Bottom tenth (least active)	3,643	0.00 (reference)		0.00 (reference)		0.00 (reference)	
2nd tenth	3,742	-0.69	-0.87, -0.52	-0.99	-1.23, -0.74	-1.97	-2.45, -1.50
3rd tenth	3,410	-0.91	-1.09, -0.73	-1.42	-1.68, -1.17	-2.89	-3.38, -2.41
4th tenth	3,677	-1.22	-1.40, -1.05	-1.97	-2.22, -1.72	-3.81	-4.29, -3.34
5th tenth	3,522	-1.32	-1.50, -1.14	-2.14	-2.39, -1.88	-4.47	-4.95, -3.99
6th tenth	3,584	-1.45	-1.63, -1.27	-2.50	-2.75, -2.24	-4.79	-5.27, -4.31
7th tenth	3,596	-1.59	-1.76, -1.41	-2.88	-3.13, -2.63	-5.41	-5.89, -4.93
8th tenth	3,591	-1.63	-1.81, -1.45	-3.09	-3.34, -2.83	-5.92	-6.40, -5.44
9th tenth	3,595	-1.71	-1.89, 1.53	-3.21	-3.46, -2.95	-6.33	-6.81, -5.85
Top tenth (most active)	3,595	-1.77	-1.95, -1.59	-3.56	-3.81, -3.31	-6.42	-6.90, -5.93
**Accelerometer**							
Bottom tenth (least active)	3,598	0.00 (reference)		0.00 (reference)		0.00 (reference)	
2nd tenth	3,604	-0.77	-0.95, -0.60	-0.99	-1.24, -0.75	-2.23	-2.70, -1.76
3rd tenth	3,592	-1.33	-1.50, -1.15	-1.83	-2.07, -1.58	-3.69	-4.16, -3.22
4th tenth	3,610	-1.60	-1.77, -1.42	-2.20	-2.45, -1.95	-4.59	-5.06, -4.12
5th tenth	3,597	-1.88	-2.06, -1.71	-2.69	-2.94, -2.44	-5.43	-5.90, -4.96
6th tenth	3,585	-2.07	-2.24, -1.89	-3.04	-3.28, -2.79	-6.00	-6.47, -5.53
7th tenth	3,589	-2.32	-2.49, -2.14	-3.52	-3.77, -3.27	-7.01	-7.49, -6.54
8th tenth	3,594	-2.57	-2.75, -2.39	-3.84	-4.09, -3.59	-7.69	-8.17, -7.22
9th tenth	3,591	-3.02	-3.20, -2.84	-4.75	-5.00, -4.50	-9.01	-9.48, -8.53
Top tenth (most active)	3,595	-3.61	-3.79, -3.43	-5.98	-6.24, -5.73	-11.23	-11.72, -10.75

**Women**							
**Questionnaire**							
Bottom tenth (least active)	4,433	0.00 (reference)		0.00 (reference)		0.00 (reference)	
2nd tenth	4,187	-0.81	-1.00, -0.61	-1.05	-1.33, -0.78	-1.77	-2.25, -1.29
3rd tenth	4,278	-1.37	-1.57, -1.18	-1.89	-2.16, -1.61	-3.33	-3.81, -2.86
4th tenth	4,318	-1.44	-1.64, -1.25	-2.16	-2.43, -1.88	-3.74	-4.22, -3.27
5th tenth	4,296	-1.60	-1.79, -1.40	-2.44	-2.72, -2.17	-3.92	-4.39, -3.44
6th tenth	4,308	-1.86	-2.05, -1.66	-2.89	-3.16, -2.61	-4.71	-5.18, -4.23
7th tenth	4,276	-1.98	-2.18, -1.79	-3.18	-3.45, -2.90	-5.02	-5.50, -4.55
8th tenth	4,300	-2.20	-2.39, -2.00	-3.57	-3.85, -3.30	-5.73	-6.20, -5.25
9th tenth	4,305	-2.28	-2.48, -2.09	-3.86	-4.14, -3.59	-5.91	-6.39, -5.44
Top tenth (most active)	4,291	-2.51	-2.70, -2.31	-4.25	-4.52, -3.97	-6.39	-6.87, -5.91
**Accelerometer**							
Bottom tenth (least active)	4,314	0.00 (reference)		0.00 (reference)		0.00 (reference)	
2nd tenth	4,292	-1.14	-1.33, -0.95	-1.43	-1.70, -1.16	-3.02	-3.49, -2.56
3rd tenth	4,293	-1.84	-2.03, -1.65	-2.35	-2.62, -2.09	-4.42	-4.89, -3.95
4th tenth	4,307	-2.31	-2.50, -2.12	-2.90	-3.17-2.63	-5.43	-5.90, -4.97
5th tenth	4,312	-2.60	-2.79, -2.41	-3.52	-3.79, -3.25	-6.27	-6.74, -5.80
6th tenth	4,286	-2.92	-3.11, -2.72	-4.05	-4.32, -3.79	-7.18	-7.65, -6.71
7th tenth	4,292	-3.22	-3.42, -3.04	-4.63	-4.90, -4.36	-7.88	-8.35, -7.41
8th tenth	4,301	-3.60	-3.80, -3.41	-5.34	-5.61, -5.07	-8.80	-9.27, -8.33
9th tenth	4,305	-4.17	-4.36, -3.98	-6.37	-6.64, -6.10	-10.12	-10.59, -9.64
Top tenth (most active)	4,290	-4.80	-4.99, -4.60	-8.06	-8.33, -7.78	-11.92	-12.39, -11.44

Analyses are adjusted for age, socioeconomic status, alcohol intake, smoking status, educational qualifications, and employment status. Analyses are further adjusted for parity and hormone replacement therapy use in women.
