# Supplementary material for: Accelerometer compared with questionnaire measures of physical activity in relation to body size and composition: a large cross-sectional analysis of UK Biobank
Source: BMJ Open. 2019 Jan 29;9(1):e024206. doi: 10.1136/bmjopen-2018-024206 (PMC6352868; doi:10.1136/bmjopen-2018-024206)
Supplement: Supplementary file 6 [file bmjopen-2018-024206supp006.pdf]

Supplementary Table 5. Association of accelerometer-measured physical activity with body fat percent, measured by bioelectrical impedance and dual energy X-ray absorptiometry (DXA) in UK Biobank

Accelerometer-measured physical activity	N	Mean body fat percent	95% CI	Coefficient	95% CI
**Men**		**Measured by impedance**			
Bottom fifth (least active)	237	27.4	26.7, 28.0	0.00 (reference group)	
2nd fifth	231	26.0	25.3, 26.6	-1.29	-2.21, -0.38
3rd fifth	229	24.7	24.0, 25.3	-2.63	-3.55, -1.71
4th fifth	253	23.8	23.1, 24.4	-3.46	-4.36, -2.56
Top fifth (most active)	235	21.8	21.1, 22.4	-5.51	-6.45, -4.58
**Men**		**Measured by DXA**			
Bottom fifth (least active)	237	34.1	33.3, 34.8	0.00 (reference group)	
2nd fifth	231	31.6	30.9, 32.4	-2.38	-3.45, -1.31
3rd fifth	229	30.0	29.2, 30.7	-4.12	-5.20, -3.05
4th fifth	253	28.7	28.0, 29.4	-5.48	-6.54, -4.42
Top fifth (most active)	235	26.1	25.4, 26.9	-8.18	-9.27, -7.08
**Women**		**Measured by impedance**			
Bottom fifth (least active)	270	38.4	37.6, 39.1	0.00 (reference group)	
2nd fifth	244	36.6	35.9, 37.4	-1.46	-2.55, -0.37
3rd fifth	265	36.1	35.4, 36.9	-1.93	-3.01, -0.85
4th fifth	228	34.5	33.7, 35.3	-3.43	-4.55, -2.31
Top fifth (most active)	265	30.8	30.1, 31.6	-6.97	-8.07, -5.87
**Women**		**Measured by DXA**			
Bottom fifth (least active)	270	42.5	41.7, 43.3	0.00 (reference group)	
2nd fifth	244	40.4	39.6, 41.2	-1.87	-3.04, -0.71
3rd fifth	265	39.8	39.0, 40.6	-2.55	-3.70, -1.40
4th fifth	228	37.1	36.2, 37.9	-4.98	-6.17, -3.78
Top fifth (most active)	265	33.3	32.5, 34.1	-8.83	-10.0, -7.65

Analyses are adjusted for age, socioeconomic status, alcohol intake, smoking status, educational qualifications, and employment status. Analyses are further adjusted for parity and hormone replacement therapy use in women.
