# Supplementary material for: Accelerometer compared with questionnaire measures of physical activity in relation to body size and composition: a large cross-sectional analysis of UK Biobank
Source: BMJ Open. 2019 Jan 29;9(1):e024206. doi: 10.1136/bmjopen-2018-024206 (PMC6352868; doi:10.1136/bmjopen-2018-024206)
Supplement: Author's manuscript [file bmjopen-2018-024206.draft_revisions.pdf]

BMJ Open is committed to open peer review. As part of this commitment we make the peer review history of every article we publish publicly available.

When an article is published we post the peer reviewers' comments and the authors' responses online. We also post the versions of the paper that were used during peer review. These are the versions that the peer review comments apply to.

The versions of the paper that follow are the versions that were submitted during the peer review process. They are not the versions of record or the final published versions. They should not be cited or distributed as the published version of this manuscript.

BMJ Open is an open access journal and the full, final, typeset and author-corrected version of record of the manuscript is available on our site with no access controls, subscription charges or pay-per-view fees (http://bmjopen.bmj.com).

If you have any questions on BMJ Open's open peer review process please email info.bmjopen@bmj.com

Accelerometer compared with questionnaire measures of physical activity in relation to body size and composition: a large cross-sectional analysis of UK Biobank

Journal:	*BMJ Open*
Manuscript ID	bmjopen-2018-024206
Article Type:	Research
Date Submitted by the Author:	15-May-2018
Complete List of Authors:	Guo, Wenji; University of Oxford, Nuffield Department of Population Health<br>Key, Timothy; University of Oxford, Nuffield Department of Population Health<br>Reeves, Gillian; University of Oxford, Nuffield Department of Population Health
Keywords:	EPIDEMIOLOGY, adiposity, physical activity, accelerometer, activity monitor

SCHOLARONE™
Manuscripts

For peer review only

Accelerometer compared with questionnaire measures of physical activity in relation to body size and composition: a large cross-sectional analysis of UK Biobank

Wenji Guo, Gillian K Reeves, Timothy J Key

Cancer Epidemiology Unit, Nuffield Department of Population Health, University of Oxford, Oxford, UK (WG, GKR, TJK)

Corresponding author:

Wenji Guo

Cancer Epidemiology Unit

Nuffield Department of Population Health

University of Oxford

Richard Doll Building

Roosevelt Drive

Oxford OX3 7LF, UK

Tel +44 1865 289635

wenji.guo@ndph.ox.ac.uk

wguo8@jhmi.edu

Abbreviations: body mass index (BMI), confidence interval (CI), dual-energy X-ray absorptiometry (DXA), International Physical Activity Questionnaire (IPAQ), metabolic equivalents (METs), standard deviation (SD)

For peer review only

Abstract

Objectives: Previous studies of the association between physical activity and adiposity are largely based on self-reported physical activity and body mass index (BMI) from questionnaires, which are prone to inaccurate and biased reporting. We assessed the associations of accelerometer-measured compared to questionnaire-measured physical activity with BMI, waist circumference and body fat percent measured by bioelectrical impedance and dual-energy X-ray absorptiometry (DXA).

Design: Cross-sectional analysis of UK Biobank participants

Setting: UK Biobank assessment centers

Participants: 78,947 UK Biobank participants (35,955 men and 42,992 women) aged 40-70 at recruitment, who had physical activity measured by both questionnaire and accelerometer.

Main outcome measures: BMI, waist circumference and body fat percent measured by bioelectrical impedance and DXA

Results: Correlation between accelerometer and questionnaire measures of physical activity was low overall and even lower in participants with higher BMI and in older participants. Greater physical activity was associated with lower adiposity. Women in the top tenth of accelerometer-measured physical activity had a 4.8 (95% CI: 4.6, 5.0) kg/m^2 lower BMI, 8.1% (95% CI: 7.8, 8.3) lower body fat percent, and 11.9 (95% CI 11.4, 12.4) cm lower waist circumference while women in the top tenth of questionnaire-measured physical activity had a 2.5 (95% CI: 2.3, 2.7) kg/m^2 lower BMI, 4.3% (95% CI: 4.0, 4.5) lower body fat percent, and 6.4 (95% CI: 5.9, 6.9) cm lower waist circumference, compared to women in the bottom tenths. The patterns were similar in men and also similar with body fat percent measured by DXA compared to impedance.

For peer review only - http://bmjopen.bmj.com/site/about/guidelines.xhtml

For peer review only

Conclusion: Our findings of approximately twofold stronger associations between physical activity and adiposity with objectively-measured compared to self-reported physical activity demonstrate substantial measurement error in self-reported physical activity, especially among participants with higher BMI and among older participants, and further emphasizes the need to incorporate objective measures in future studies.

For peer review only

Strengths of limitations of this study:

- Most large studies of physical activity are based on self-reported data from questionnaires, which are prone to inaccurate and potentially biased reporting.
- This study is by far the largest study to compare associations between physical activity objectively measured by accelerometer and self-reported physical activity in relation to various measures of adiposity, including body fat percent assessed by bioelectrical impedance and dual energy x-ray absorptiometry.
- Due to the cross-sectional nature of this study, we cannot assess to what extent physical activity is causally related to adiposity.

For peer review only - http://bmjopen.bmj.com/site/about/guidelines.xhtml

INTRODUCTION

The prevalence of overweight and obesity is high worldwide and is associated with increased risk of various conditions including heart disease, stroke, hypertension, diabetes, and some cancers (1, 2). Although physical activity is generally accepted to be important for prevention of weight gain, achievement of modest weight loss, and prevention of weight regain after weight loss (3), randomized controlled trials have shown inconsistent results, perhaps partly due to limited duration of interventions and difficulty in long-term adherence to exercise regimens (4), and previous large-scale observational studies are mostly based on self-reported physical activity from questionnaires, which are prone to both inaccurate reporting and reporting bias (5).

Prior studies have demonstrated low to moderate correlation between self-reported and objective accelerometer measures of physical activity (6, 7). Self-reported and accelerometer-measured physical activity capture different aspects of physical activity with limitations unique to each (7). However, research methods utilizing more objective measures of physical activity, along with more detailed measures of body fat, are needed to reduce measurement error and more accurately characterize the association between physical activity and adiposity.

We examined the association between physical activity and adiposity, with accelerometer-measured compared to self-reported physical activity in nearly 80,000 participants. These associations were assessed using various measures of adiposity, including BMI, waist circumference, and body fat percent measured by both bioelectrical impedance and dual-energy X-ray absorptiometry (DXA). We also explored how the associations vary by age.

For peer review only - http://bmjopen.bmj.com/site/about/guidelines.xhtml

METHODS

Data source

Data were obtained from UK Biobank. Details of UK Biobank design, rationale, and survey methods have been described elsewhere (8). Information on data available and access procedures are described on the study website (http://www.ukbiobank.ac.uk/). UK Biobank has approval from the National Information Governance Board for Health & Social Care in England and Wales, the North West Multi-centre Research Ethics Committee, and the Community Health Index Advisory Group in Scotland. Written informed consent was provided by all participants.

Study participants

The complete UK Biobank dataset includes 502,617 UK adults (229,164 men and 273,453 women) between 40 to 70 years of age at recruitment during 2006 to 2010. During the baseline assessment center visit, participants completed a touchscreen questionnaire which included questions on socio-demographics, lifestyle, health and medical history, and sex-specific factors. The present study was restricted to participants with available accelerometer data (n=103,705). Participants were excluded if they did not have at least 72 hours of data and also data in each one-hour period of the 24-hour cycle across multiple days (n=6,995). Participants were also excluded if they had insufficient data for calibration (n=4). Participants who had missing data on any of the physical activity variables used in our analyses were excluded (n=15,999). Participants who reported physical activity greater than an average of 16 hours per day (n=620) were also excluded as recommended by the International Physical Activity Questionnaire (IPAQ) scoring guidelines, which can be accessed at file:///H:/Downloads/GuidelinesforDataProcessingandAnalysisoftheInternationalPhysicalActivityQuestionnaireIPAQShortandLongForms.pdf. Finally, participants with missing data on

For peer review only

BMI (n=146), body fat percent (n=988), and waist circumference (n=6) were excluded. The analyses included 35,955 men and 42,992 women (**Supplementary Figure 1**).

Self-reported physical activity

Physical activity questions from the baseline questionnaire captured the frequency and duration of three intensities of activity (walking, moderate, and vigorous). Participants were asked how many days per week they typically engaged in each category of activity. For each category in which an answer of one or more days was given, the participant was subsequently asked the number of minutes on average spent on the activity per day. Questions were adapted from the IPAQ, a validated survey instrument (9), and are listed in **Supplementary Table 1**. Metabolic equivalents (METs) were used to quantify physical activity; 1 MET is expended by sitting quietly for 1 hour, and the MET value reflects the ratio of energy expended per kilogram of body weight per hour to that expended when sitting quietly (10). The number of minutes per day engaged in each level of activity was multiplied by the respective MET score for the corresponding level of activity (3.3 for walking, 4.0 for moderate physical activity, and 8.0 for vigorous physical activity). MET minutes per day were converted to MET hours per week. The total amount of METs was calculated by summing total METs from the walking, moderate, and vigorous activity levels. Following IPAQ scoring guidelines, physical activity of less than 10 minutes per day for any category was recoded to 0.

Accelerometer-measured physical activity

A total of 236,519 participants, all of whom had provided a valid email address, were invited to participate in a seven day accelerometer study between February 2013 and December 2015 (on average, approximately 5.5 years after recruitment). Starting in June 2013, participants were sent wrist-worn triaxial accelerometers (Axivity AX3, Newcastle

For peer review only - http://bmjopen.bmj.com/site/about/guidelines.xhtml

For peer review only

upon Tyne, UK) that were programmed to capture three-dimensional acceleration data at 100 Hz with a dynamic range of ±8 g. Participants were also given instructions to wear the accelerometer on their dominant wrist continuously for seven days and then to send the device to the coordinating center using the provided prepaid envelope. Further details on data collection, processing, and analysis can be found elsewhere (11).

Anthropometry and body composition

At the UK Biobank baseline interview, trained staff measured standing height using the Seca 202 device (Seca, Hamburg, Germany). BMI was calculated by dividing weight (kg) by the square of standing height (m^2). The Wessex non-stretchable sprung tape measure (Wessex, United Kingdom) was used to measure waist circumference. The Tanita BC-418MA body composition analyzer (Tanita, Tokyo, Japan) was used to measure body fat percent using bioelectrical impedance. DXA was used to measure fat percent on a subset of participants beginning in 2014 using the GE-Lunar iDXA (GE Healthcare, Chicago, USA).

Statistical analyses

Baseline characteristics were summarized by physical activity (least active fifth, most active fifth, and overall) separately for men and women. Since self-reported physical activity was not normally distributed, Spearman's correlation coefficients were used to measure the strength of correlations between self-reported and accelerometer-measured physical activity in the overall population and in subgroups based on sociodemographic characteristics.

Self-reported and accelerometer-measured physical activity were categorized into tenths and the median value within each category of physical activity is shown in the figures. The associations of physical activity and adiposity measures were examined using multivariable linear regression, separately in men and women. Analyses comparing the association of

For peer review only - http://bmjopen.bmj.com/site/about/guidelines.xhtml

accelerometer-measured physical activity with body fat percent, measured by bioelectrical impedance and DXA were restricted to participants with both measures. Likelihood ratio tests were used to assess whether the associations between physical activity and adiposity were modified by age (<55 years or 55+ years), separately for self-reported and accelerometer-measured physical activity.

Covariates were determined a priori and were 5-year age at recruitment categories, socioeconomic status as indicated by fifths of Townsend deprivation index (12), educational qualifications, employment status, smoking status (never, previous, current), and alcohol intake frequency. Analyses in women were additionally adjusted for parity (nulliparous, 1, 2, 3, 4 or more births) and hormone replacement therapy use (never, previous, current). As a covariate, educational qualification was grouped into the following categories: vocational qualifications, national exams at age 16 (O levels, GCSEs, CSEs, or equivalent), optional national exams at ages 17-18 years (A levels, AS levels, or equivalent), and college or university degree. Employment status was categorized as paid or self-employed, retired, looking after home and/or family, unemployed, doing unpaid or voluntary work, unable to work due to sickness or disability, and student. Alcohol intake was categorized as never, special occasions only, 1-3 times a month, 1-2 times a week, 3-4 times a week, and daily or almost daily.

Missing data were grouped in a separate unknown category for each covariate. There were less than 1% missing data for all covariates except for educational qualifications (7.4% missing data). To assess the impact of missing values, a sensitivity analysis restricted to participants with known values for all covariates was conducted. We also conducted sensitivity analyses to assess the impact of excluding participants who reported long-term illness, disability or infirmity, participants who reported fair or poor health rather than excellent or good health, and participants whose jobs usually or always required heavy

For peer review only

For peer review only - http://bmjopen.bmj.com/site/about/guidelines.xhtml

manual work. Analyses were conducted using STATA, version 15.0 (Stata Corp LP, College Station, TX).

Patient and public involvement

This study did not involve patients and the public.

RESULTS

Characteristics of the study population by least active and most active fifth of accelerometer-measured physical activity are shown in **Table 1**. Mean accelerometer-measured physical activity was 27.6 (standard deviation [SD] 8.7) milli-gravity in men and 28.7 (SD 8.0) milli-gravity in women. The most active participants were on average younger and had lower values for all body size and composition variables. They were more likely to have a college or university degree, be employed rather than retired, have an active job, and consume alcohol at least weekly. The least active participants were more likely to be ever smokers and were also more likely to have a long-standing illness or disability. The correlation between questionnaire and accelerometer-measured physical activity was 0.24 (95% confidence interval [CI]: 0.23, 0.25) in men (**Supplementary Table 2**) and 0.22 (95% CI: 0.21, 0.23) in women (**Supplementary Table 3**). The correlations were comparatively higher in participants who were younger and in participants who had lower BMI. The correlations were lower among participants who reported that their job usually or always involved heavy manual work and/or mainly walking or standing.

The inverse associations between physical activity and all measures of adiposity were linear and approximately twofold larger in models that used accelerometer-measured rather than self-reported physical activity. Since there was heterogeneity in the associations between both self-reported and accelerometer-measured physical activity and adiposity by sex

For peer review only - http://bmjopen.bmj.com/site/about/guidelines.xhtml

(P<0.001), separate analyses were performed in men and women. The mean differences in BMI and body fat percent were greater in women compared to men. Comparing the top to bottom tenth of accelerometer-measured physical activity, the difference in BMI was 4.8 (95% CI: 4.6, 5.0) kg/m^2 in women and 3.6 (95% CI: 3.4, 3.8) kg/m^2 in men (**Figure 1, Supplementary Table 4**).

Women in the top tenth of accelerometer-measured physical activity had an 8.1% (95% CI: 7.8, 8.3) lower body fat percent while women in the top tenth of self-reported physical activity had a 4.3% (95% CI: 4.0, 4.5) lower body fat percent, compared to those in the bottom tenth of physical activity. Men in the top tenth of accelerometer-measured physical activity had a 6.0% (95% CI: 5.7, 6.2) lower body fat percent while men in the top tenth of self-reported physical activity had a 3.6% (95% CI: 3.3, 3.8) lower body fat percent, compared to those in the bottom tenth (**Figure 1, Supplementary Table 4**).

Associations between physical activity and waist circumference were of similar magnitude in men and women, with an approximately twofold larger inverse association between waist circumference and physical activity when measured by accelerometer rather than questionnaire (**Figure 1, Supplementary Table 4**).

The results of sensitivity analyses excluding participants who had any missing values, reported a long-term illness or disability, reported a health rating worse than "good", or whose jobs usually or always required heavy manual work did not materially differ from the main findings.

Figure 2 and Supplementary Table 5 show the associations between accelerometer-measured physical activity and bioelectrical impedance-measured body fat percent at baseline (2006-2010) compared to body fat percent measured by DXA starting in May 2014. Body fat percent by impedance at baseline was lower than body fat percent by DXA, measured on

For peer review only - http://bmjopen.bmj.com/site/about/guidelines.xhtml

average seven years later. For both measures, there was a linear dose-response association between physical activity and body fat percent in both men and women. The inverse associations were stronger when body fat percent was measured by DXA. Compared to the least active women, the most active women had an 8.8% (95% CI: 7.7, 10.0) lower DXA-measured body fat percent and a 7.0% (95% CI: 5.9, 8.1) lower impedance-measured body fat percent (**Figure 2 and Supplementary Table 5**).

Associations between physical activity and measures of adiposity by age group are shown in **Figure 3** for men and **Figure 4** for women. For a given level of accelerometer-measured physical activity, the older participants (over age 55) had a slightly lower BMI but a higher body fat percent compared to their younger counterparts. For women, there was heterogeneity by age in the association between self-reported physical activity and body fat percent (*P*=0.03) but there was no heterogeneity by age when physical activity was measured by accelerometer (*P*=0.27).

DISCUSSION

In this large cross-sectional study of nearly 80,000 participants, we found that associations between physical activity and BMI, body fat percent, and waist circumference were stronger when physical activity was measured by accelerometer compared to questionnaire self-reports. Body fat percent measured by DXA at follow-up showed a slightly stronger association with physical activity compared to body fat percent measured by bioelectrical impedance at baseline, but the overall pattern of association was similar. The correlation between accelerometer-measured and self-reported physical activity was lower in participants with higher BMI and in older participants.

Our analyses based on accelerometer-measured physical activity suggest an approximately linear inverse association between physical activity and adiposity, with the most active

For peer review only - http://bmjopen.bmj.com/site/about/guidelines.xhtml

For peer review only

participants having the lowest BMI, body fat percent, and waist circumference. In contrast, the analyses in the same participants based on self-reported physical activity suggest a comparatively small further benefit of physical activity greater than 50 MET-hours a week on adiposity.

We have previously suggested that the steeper relationship between physical activity and lower adiposity within the lower range of physical activity could be due to either a comparatively larger benefit of physical activity for those who are relatively inactive or measurement error from over-reporting of high physical activity (13). The present analyses demonstrating an approximately linear dose-response relationship between accelerometer-measured physical activity and adiposity supports the latter explanation and further suggests that over-reporting of total physical activity contributed to the low overall correlation between self-reported and accelerometer-measured physical activity. Although wrist accelerometer-measured physical activity has limitations, such as measuring movement of only one part of the body and the inability to reliably capture activities such as cycling (7), it has the major advantage of eliminating both inaccurate reporting that leads to random error as well as reporting bias that may vary by sociodemographic characteristics.

Measurement error in the self-reported data results in misclassification of individuals by physical activity status. We used the IPAQ short form data processing rules since the UK Biobank questionnaire did not comprehensively cover domain-specific activities, but it is still likely that lower intensity activities were underreported and reported less accurately (14). In contrast, the accelerometers were worn for 24 hours a day, over 7 days. Therefore, the lower correlation between self-reported and accelerometer-measured physical activity in older participants and the heterogeneity by age seen only with the self-reported data may be explained by the observation that, in older adults, a greater proportion of physical activity is of lower intensity (15).

For peer review only - http://bmjopen.bmj.com/site/about/guidelines.xhtml

Individuals with higher body fat percent may report moderate and strenuous physical activity less accurately than leaner individuals, based on comparisons between self-reported physical activity and energy expenditure estimated from whole-room indirect calorimeter (16). In agreement with some previous studies, we found that the correlation between physical activity measured by questionnaire and accelerometer-measured physical activity was greater for those with lower BMI (7). This suggests that measurement error of self-reported physical activity may be greater in overweight and obese BMI groups.

We, like several prior studies, found stronger associations between accelerometer-measured physical activity and all measures of adiposity in women compared to men (17-19). This may partly be due to the fact that, in the present study, men were on average objectively less physically active than women. Differences in fat metabolism may also play a role, with a greater proportion of energy derived from lipolysis during exercise in women compared to men (20, 21).

To our knowledge, the present study is the largest to date comparing accelerometer-measured and self-reported physical activity in relation to direct measures of body fat, although our results are consistent with prior, smaller studies that suggest a stronger association between adiposity and accelerometer-measured compared to self-reported physical activity (17, 19, 22-24). A major strength of this study is the availability of both accelerometer-measured physical activity and body fat by impedance in nearly 80,000 participants, together with data on body fat assessed by DXA in over 2,400 participants. Additionally, the accelerometers used in this study were waterproof (11), overcoming a limitation of prior studies where the devices had to be removed for water-based activities (19).

For peer review only - http://bmjopen.bmj.com/site/about/guidelines.xhtml

While self-reported physical activity was available at baseline in these data, accelerometer-measured physical activity was assessed only 3-5 years after end of recruitment, which raises the question of whether higher adiposity at baseline predicts lower physical activity levels (25) rather than physical activity determining adiposity. However, our analysis of accelerometer-measured physical activity in relation to DXA-measured body fat percent, which was assessed within the same time frame as accelerometer-measured physical activity, showed similar results to the main analysis based on body fat percent assessed by impedance at baseline. Other limitations include the lack of data on total energy intake for the whole cohort. Due to the cross-sectional nature of this study, we cannot assess to what extent physical activity is causally related to adiposity. Highly active individuals may also be more likely to maintain appropriate target energy intake, for example. Although the UK Biobank cohort may not be representative of the sampling population, results of associations between exposures and health outcomes may be generalizable and would not necessarily require the study population to be representative if the biological basis of the exposure-disease relationship is shared.

In conclusion, our findings based on objective accelerometer data indicate a stronger relationship between physical activity and adiposity than previously thought. Comparison of results with physical activity measured by questionnaire and accelerometer suggest substantial measurement error in self-reported physical activity, emphasizing the need to incorporate objective measures of physical activity in future studies.

Funding: This work was supported by Cancer Research UK, grant number C570/A16491. WG is supported by the Clarendon Fund.

Disclosures: The authors have declared no conflicts of interest.

For peer review only - http://bmjopen.bmj.com/site/about/guidelines.xhtml

Contributors: WG, TJK, and GKR were responsible for study concept, design of the study, interpretation of the data, and manuscript writing. WG had primary responsibility for statistical analysis and final content. All authors reviewed and approved the final manuscript.

References

1. Must A, Spadano J, Coakley EH, et al. The disease burden associated with overweight and obesity. *JAMA* 1999;282(16):1523-9.
2. Afshin A, Forouzanfar MH, Reitsma MB, et al. Health effects of overweight and obesity in 195 countries over 25 years. *N Engl J Med* 2017;377(1):13-27.
3. Donnelly JE, Blair SN, Jakicic JM, et al. American College of Sports Medicine position stand. Appropriate physical activity intervention strategies for weight loss and prevention of weight regain for adults. *Med Sci Sports Exerc* 2009;41(2):459-71.
4. Catenacci VA, Wyatt HR. The role of physical activity in producing and maintaining weight loss. *Nat Clin Pract End Met* 2007;3(7):518-29.
5. Ainsworth BE, Caspersen CJ, Matthews CE, et al. Recommendations to improve the accuracy of estimates of physical activity derived from self report. *J Phys Act Health* 2012;9 Suppl 1:S76-84.
6. Koolhaas CM, van Rooij FJ, Cepeda M, et al. Physical activity derived from questionnaires and wrist-worn accelerometers: comparability and the role of demographic, lifestyle, and health factors among a population-based sample of older adults. *Clin Epidemiol* 2018;10:1-16.
7. Skender S, Ose J, Chang-Claude J, et al. Accelerometry and physical activity questionnaires - a systematic review. *BMC Public Health* 2016;16:515.
8. UK Biobank. Protocol for a large-scale prospective epidemiological resource. 2010. (http://www.ukbiobank.ac.uk/wp-content/uploads/2011/11/UK-Biobank-Protocol.pdf). (Accessed 21 March 2014).
9. Craig CL, Marshall AL, Sjostrom M, et al. International physical activity questionnaire: 12-country reliability and validity. *Medicine & Science in Sports & Exercise* 2003;35(8):1381-95.
10. Ainsworth BE, Haskell WL, Herrmann SD, et al. 2011 Compendium of physical activities: a second update of codes and MET values. *Medicine & Science in Sports & Exercise* 2011;43(8):1575-81.
11. Doherty A, Jackson D, Hammerla N, et al. Large scale population assessment of physical activity using wrist worn accelerometers: the UK Biobank Study. *PLoS One* 2017;12(2):e0169649.
12. Morris R, Carstairs V. Which deprivation? A comparison of selected deprivation indexes. *J Public Health Med* 1991;13(4):318-26.
13. Guo W, Bradbury KE, Reeves GK, et al. Physical activity in relation to body size and composition in women in UK Biobank. *Annals of Epidemiology* 2015;25(6):406-13 e6.
14. Sallis JF, Saelens BE. Assessment of physical activity by self-report: status, limitations, and future directions. *Res Q Exerc Sport* 2000;71.
15. Westerterp KR. Physical activity as determinant of daily energy expenditure. *Physiol Behav* 2008;93(4-5):1039-43.
16. Buchowski MS, Townsend KM, Chen KY, et al. Energy expenditure determined by self-reported physical activity is related to body fatness. *Obes Res* 1999;7.

For peer review only - http://bmjopen.bmj.com/site/about/guidelines.xhtml

17. Sabia S, Cogranne P, van Hees VT, et al. Physical activity and adiposity markers at older ages: accelerometer vs questionnaire data. *J Am Med Dir Assoc* 2015;16(5):438.e7-13.
18. Bann D, Kuh D, Wills AK, et al. Physical activity across adulthood in relation to fat and lean body mass in early old age: findings from the Medical Research Council National Survey of Health and Development, 1946-2010. *Am J Epidemiol* 2014;179(10):1197-207.
19. Wanner M, Richard A, Martin B, et al. Associations between self-reported and objectively measured physical activity, sedentary behavior and overweight/obesity in NHANES 2003-2006. *Int J Obes (Lond)* 2017;41(1):186-93.
20. Blaak E. Gender differences in fat metabolism. *Curr Opin Clin Nutr Metab Care* 2001;4(6):499-502.
21. Hedrington MS, Davis SN. Sexual dimorphism in glucose and lipid metabolism during fasting, hypoglycemia, and exercise. *Front Endocrinol (Lausanne)* 2015;6:61.
22. Foong YC, Aitken D, Winzenberg T, et al. The association between physical activity and reduced body fat lessens with age - results from a cross-sectional study in community-dwelling older adults. *Exp Gerontol* 2014;55:107-12.
23. Harris TJ, Owen CG, Victor CR, et al. A comparison of questionnaire, accelerometer, and pedometer: measures in older people. *Med Sci Sports Exerc* 2009;41(7):1392-402.
24. Atienza AA, Moser RP, Perna F, et al. Self-reported and objectively measured activity related to biomarkers using NHANES. *Med Sci Sports Exerc* 2011;43(5):815-21.
25. Kim Y, White T, Wijndaele K, et al. Adiposity and grip strength as long-term predictors of objectively measured physical activity in 93,015 adults: the UK Biobank study. *Int J Obes (Lond)* 2017;41(9):1361-8.

For peer review only

For peer review only - http://bmjopen.bmj.com/site/about/guidelines.xhtml

Figure legends

Figure 1. Association of self-reported and accelerometer-measured physical activity with adiposity variables in UK Biobank

Association of A) accelerometer-measured and B) self-reported physical activity with BMI

Association of C) accelerometer-measured and D) self-reported physical activity with body fat percent

Association of E) accelerometer-measured and F) self-reported physical activity with waist circumference

Physical activity was grouped into tenths, separately in men and women.

Adjusted geometric means (from linear regression models) for BMI, body fat percent, and waist circumference are plotted against the median value within each tenth of self-reported or accelerometer-measured physical activity. Adjusted geometric means are represented by squares for men and triangles for women.

These analyses are stratified by age at recruitment, region of recruitment, and socioeconomic status, and are adjusted for educational qualifications, employment status, smoking status, and alcohol intake frequency. Analyses in women are additionally adjusted for parity and hormone replacement therapy use.

The figure shows point estimates and 95% confidence intervals.

Abbreviations: BMI, body mass index; MET, metabolic equivalent

Figure 2. Association of accelerometer-measured physical activity with body fat percent measured by impedance and DXA in UK Biobank A) men and B) women

Physical activity was grouped into fifths, separately in men and women.

For peer review only - http://bmjopen.bmj.com/site/about/guidelines.xhtml
For peer review only

Adjusted geometric means (from linear regression models) for body fat percent are plotted against the median value within each fifth of accelerometer-measured physical activity. Adjusted geometric means are represented by diamonds for body fat percent measured by impedance and circles for body fat percent measured by DXA.

These analyses are restricted to participants with measures of body fat percent by both impedance and DXA. Analyses are stratified by age at recruitment, region of recruitment, and socioeconomic status, and are adjusted for educational qualifications, employment status, smoking status, and alcohol intake frequency. Analyses in women are additionally adjusted for parity and hormone replacement therapy use.

The figure shows point estimates and 95% confidence intervals.

Abbreviations: DXA, dual-energy X-ray absorptiometry

Figure 3. Association of self-reported and accelerometer-measured physical activity with adiposity variables by age group in UK Biobank men

Association of physical activity measured by A) accelerometer and B) self-reported questionnaire with BMI.

Association of physical activity measured by C) accelerometer and D) self-reported questionnaire with body fat percent.

Physical activity was grouped into tenths.

Adjusted geometric means (from linear regression models) for BMI and body fat percent are plotted against the median value within each tenth of self-reported or accelerometer-

For peer review only - http://bmjopen.bmj.com/site/about/guidelines.xhtml

measured physical activity. Adjusted geometric means are represented by diamonds for those under age 55 and squares for those ages 55 or older.

These analyses are stratified by age at recruitment, region of recruitment, and socioeconomic status, and are adjusted for educational qualifications, employment status, smoking status, and alcohol intake frequency.

The figure shows point estimates and 95% confidence intervals.

Abbreviations: BMI, body mass index; MET, metabolic equivalent

Figure 4. Association of self-reported and accelerometer-measured physical activity with adiposity variables by age group in UK Biobank women

Association of physical activity measured by A) accelerometer and B) self-reported questionnaire with BMI.

Association of physical activity measured by C) accelerometer and D) self-reported questionnaire with body fat percent.

Physical activity was grouped into tenths.

Adjusted geometric means (from linear regression models) for BMI and body fat percent are plotted against the median value within each tenth of self-reported or accelerometer-measured physical activity. Adjusted geometric means are represented by diamonds for those under age 55 and squares for those ages 55 or older.

These analyses are stratified by age at recruitment, region of recruitment, and socioeconomic status, and are adjusted for educational qualifications, employment status, smoking status, and alcohol intake frequency, parity, and hormone replacement therapy use.

For peer review only - http://bmjopen.bmj.com/site/about/guidelines.xhtml

The figure shows point estimates and 95% confidence intervals.

Abbreviations: BMI, body mass index; MET, metabolic equivalent

Supplementary Figure 1. Flowchart illustrating the application of exclusion criteria for the current study in UK Biobank

For peer review only

For peer review only - http://bmjopen.bmj.com/site/about/guidelines.xhtml

Table 1. Characteristics of the UK Biobank study population, according to fifths of accelerometer-measured physical activity.

	Least active men <20.8 milli-gravity	Most active men ≥33.7 milli-gravity	All men		Least active women <22.2 milli-gravity	Most active women ≥34.6 milli-gravity	All women
**Number of participants**	7,202	7,186	35,955		8,606	8,595	42,992
**Age at recruitment (years), mean (SD)**	59.7 (7.0)	53.4 (7.7)	56.7 (7.9)		58.0 (7.4)	52.6 (7.4)	55.3 (7.7)
**Lowest fifth of socioeconomic status**	1,520 (21.1%)	1,351 (18.8%)	6,800 (18.9%)		1,897 (22.0%)	1,699 (19.8%)	8,744 (20.3%)
**Accelerometer-measured physical activity (milli-gravity), mean (SD)**	17.5 (2.6)	40.5 (7.8)	27.6 (8.7)		18.9 (2.7)	40.6 (6.3)	28.7 (8.0)
**Self-reported physical activity (MET-hours/week), median (IQR)**	20.7 (9.0, 42.6)	44.2 (23.7, 80.9)	29.9 (14.2, 58.1)		21.3 (9.9, 41.7)	40.2 (21.8, 73.2)	29.3 (14.4, 55.3)
**Height (cm), mean (SD)**	176.3 (6.8)	176.4 (6.6)	176.5 (6.6)		163.2 (6.3)	163.7 (6.1)	163.5 (6.2)
**Weight (kg), mean (SD)**	89.4 (15.4)	80.8 (11.4)	84.9 (13.5)		75.5 (15.6)	65.0 (10.3)	69.9 (13.2)
**BMI (kg/m^2), mean (SD)**	28.8 (4.6)	25.9 (3.3)	27.2 (4.0)		28.3 (5.7)	24.3 (3.7)	26.2 (4.8)
**Body fat percent (%), mean (SD)**	27.0 (5.6)	21.7 (5.4)	24.4 (5.7)		38.7 (6.6)	31.7 (6.4)	35.3 (6.8)
**Waist circumference (cm), mean (SD)**	100.1 (11.7)	90.9 (9.3)	95.4 (10.8)		87.9 (13.3)	77.6 (9.5)	82.4 (11.7)
**College or university degree**	3,018 (41.9%)	3,365 (46.8%)	16,709 (46.5%)		3,586 (41.7%)	4,060 (47.2%)	19,214 (44.7%)
**Current employment status**							
Paid employment/self-employed	3,608 (50.1%)	5,420 (75.4%)	22,942 (63.8%)		4,401 (51.1%)	6,101 (71.0%)	26,693 (62.1%)
Retired	3,107 (43.1%)	1,451 (20.2%)	11,361 (31.6%)		3,517 (40.9%)	1,591 (18.5%)	12,710 (29.6%)
Other	487 (6.8%)	315 (4.4%)	1,652 (4.6%)		688 (8.0%)	903 (10.5%)	3,589 (8.3%)
**Job involves mainly walking/standing [a]**	707 (19.6%)	1,742 (32.1%)	5,574 (24.3%)		893 (20.3%)	1,926 (31.6%)	6,648 (24.9%)
**Job involves heavy manual work [b]**	272 (7.5%)	912 (16.8%)	2,335 (10.2%)		170 (3.9%)	576 (9.4%)	1,567 (5.9%)
**Weekly or more frequent alcohol intake**	5,545 (77.0%)	5,989 (83.3%)	29,421 (81.8%)		5,295 (61.5%)	6,292 (73.2%)	29,829 (69.4%)
**Ever smoker**	3,801 (52.8%)	3,126 (43.5%)	16,964 (47.2%)		3,583 (41.6%)	3,212 (37.4%)	16,936 (39.4%)
**Long-standing illness or disability**	3,089 (42.9%)	1,543 (21.5%)	10,825 (30.1%)		3,145 (36.5%)	1,449 (16.9%)	1,0685 (24.9%)

[a] Participants who reported their work "usually" or "always" involved walking or standing for most of the time

[b] Participants who reported their work "usually" or "always" involved heavy manual or physical work for most of the time

For peer review only

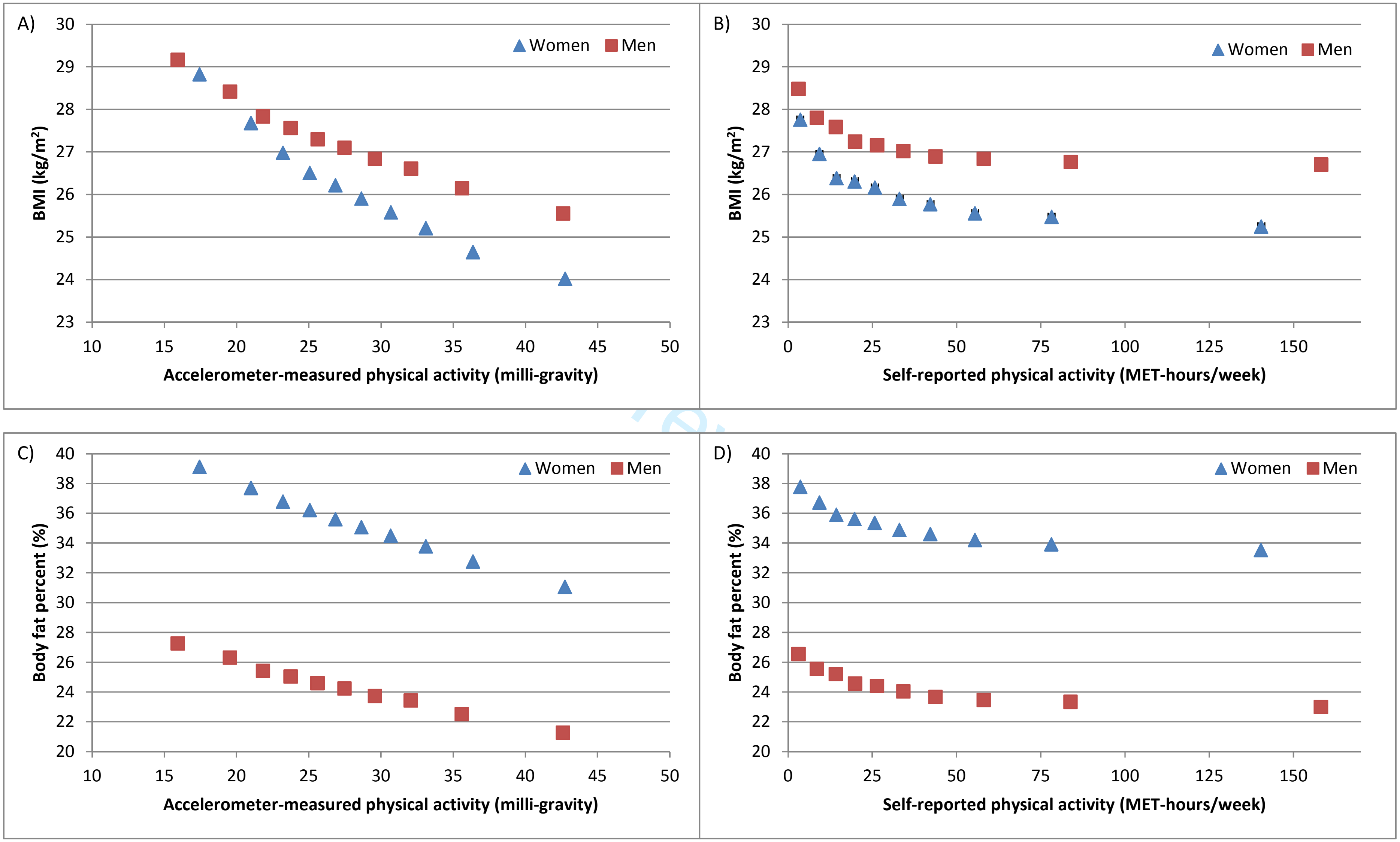
A)
BMI (kg/m²)
Women
Men
30
29
28
27
26
25
24
23
10
15
20
25
30
35
40
45
50
Accelerometer-measured physical activity (milli-gravity)
B)
BMI (kg/m²)
Women
Men
0
25
50
75
100
125
150
Self-reported physical activity (MET-hours/week)
C)
Body fat percent (%)
Women
Men
40
38
36
34
32
30
28
26
24
22
20
Accelerometer-measured physical activity (milli-gravity)
D)
Body fat percent (%)
Women
Men
Self-reported physical activity (MET-hours/week)

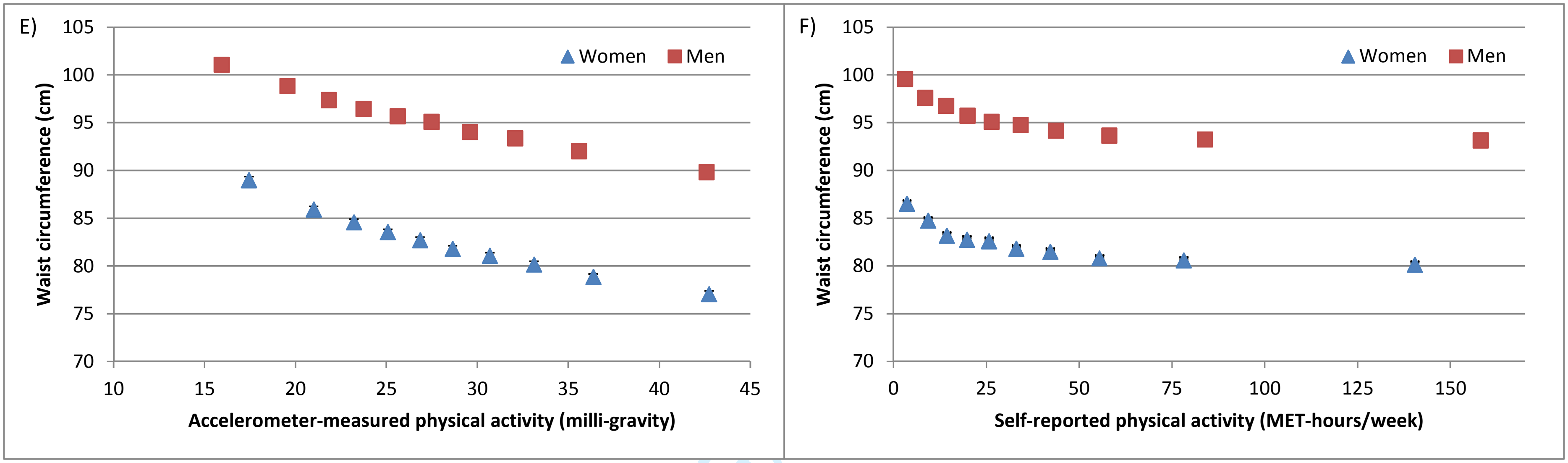

For peer review only - http://bmjopen.bmj.com/site/about/guidelines.xhtml

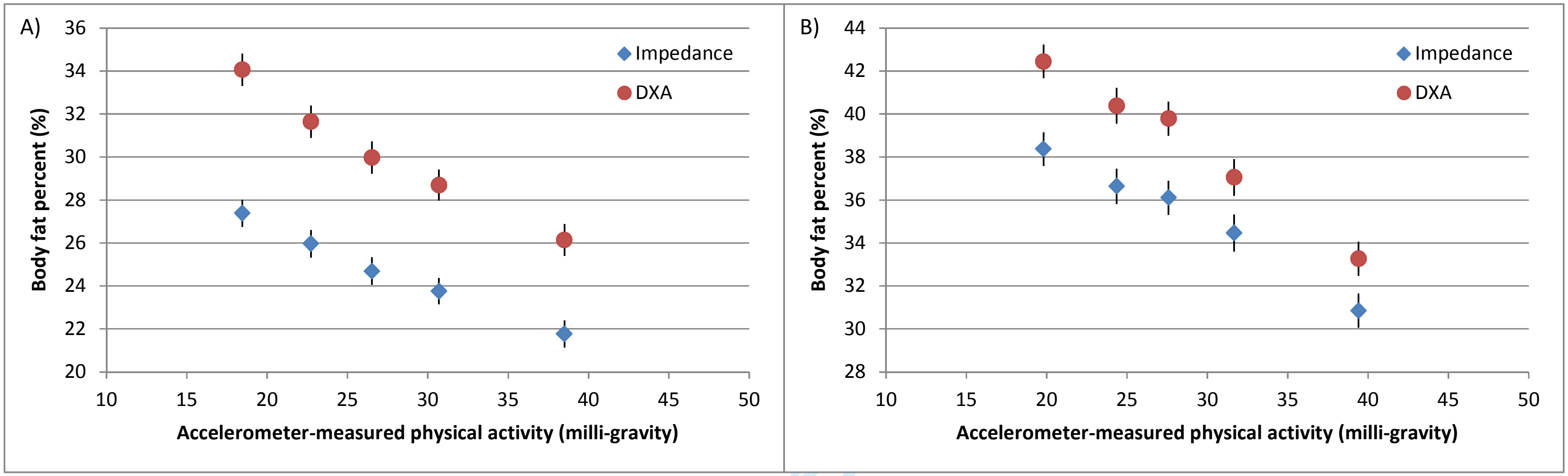

For peer review only - http://bmjopen.bmj.com/site/about/guidelines.xhtml

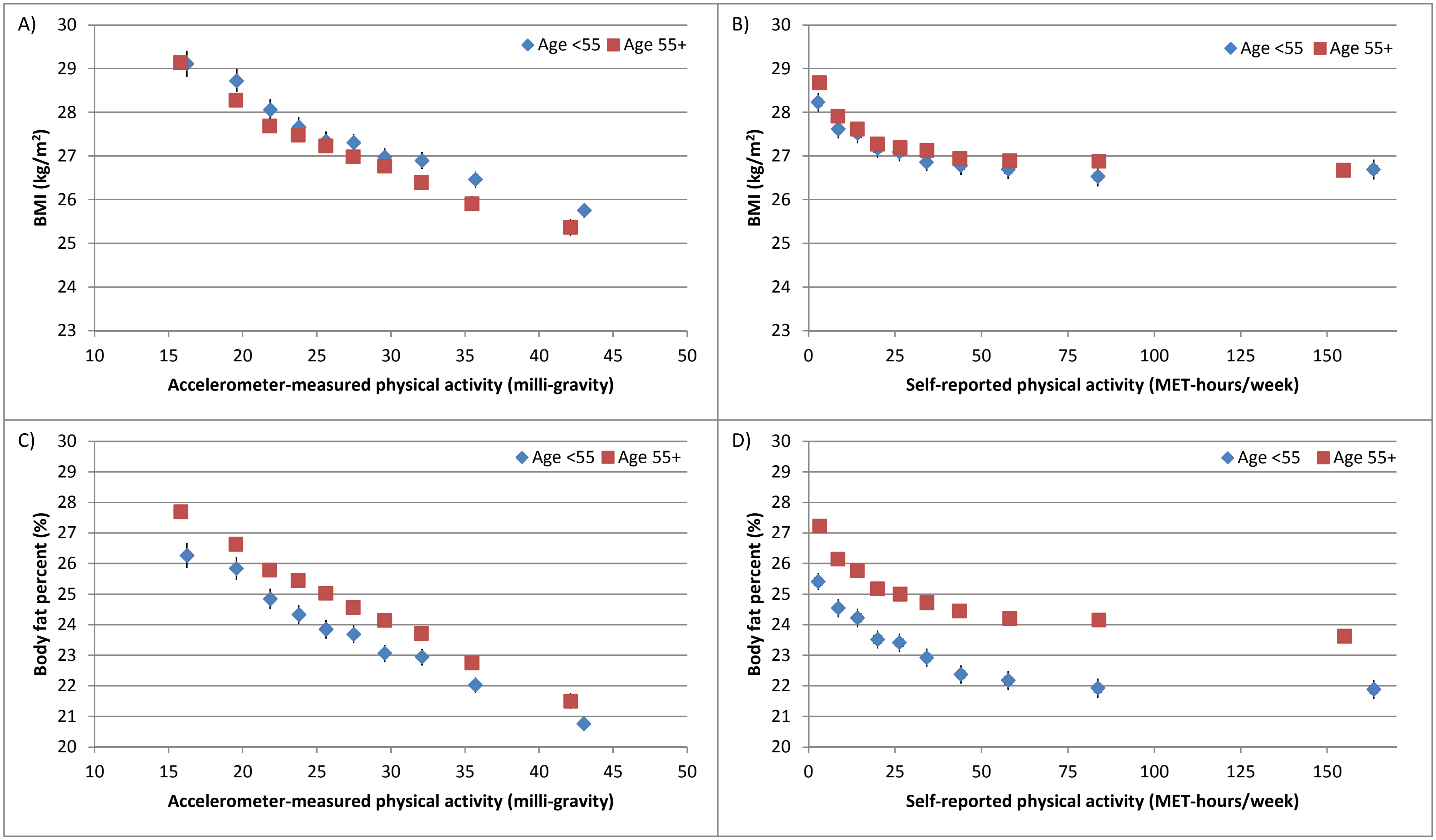
A)
BMI (kg/m²)
Age <55
Age 55+
Accelerometer-measured physical activity (milli-gravity)
B)
BMI (kg/m²)
Age <55
Age 55+
Self-reported physical activity (MET-hours/week)
C)
Body fat percent (%)
Age <55
Age 55+
Accelerometer-measured physical activity (milli-gravity)
D)
Body fat percent (%)
Age <55
Age 55+
Self-reported physical activity (MET-hours/week)

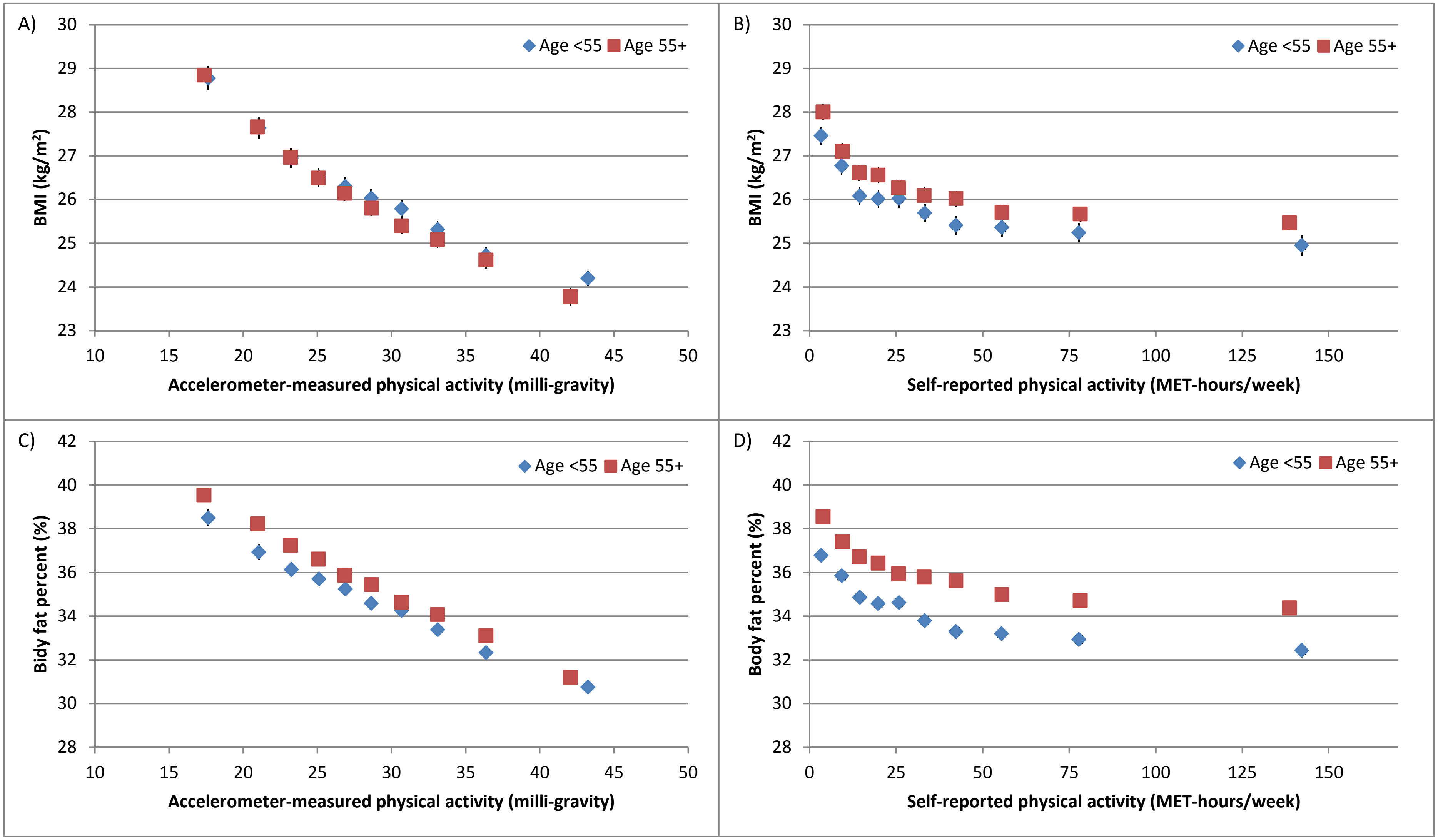
A)
Age <55
Age 55+
BMI (kg/m²)
Accelerometer-measured physical activity (milli-gravity)
B)
Age <55
Age 55+
BMI (kg/m²)
Self-reported physical activity (MET-hours/week)
C)
Age <55
Age 55+
Bidy fat percent (%)
Accelerometer-measured physical activity (milli-gravity)
D)
Age <55
Age 55+
Body fat percent (%)
Self-reported physical activity (MET-hours/week)

Supplementary Table 1. Physical activity questions from the UK Biobank baseline questionnaire

Question	Potential Responses
In a typical WEEK, on how many days did you walk for at least 10 minutes at a time? (Include walking that you do at work, travelling to and from work, and for sport or leisure)	Number of days, “Do not know”, “Unable to walk”, or “Prefer not to answer”
How many minutes did you usually spend walking on a typical DAY?	Quantity of minutes, “Do not know”, or “Prefer not to answer”
In a typical WEEK, on how many days did you do 10 minutes or more of moderate physical activities like carrying light loads, cycling at normal pace? (Do not include walking)	Number of days, “Do not know”, or “Prefer not to answer”
How many minutes did you usually spend doing moderate activities on a typical DAY?	Quantity of minutes, “Do not know”, or “Prefer not to answer”
In a typical WEEK, how many days did you do 10 minutes or more of vigorous physical activity? (These are activities that make you sweat or breathe hard such as fast cycling, aerobics, heavy lifting)	Number of days, “Do not know”, or “Prefer not to answer”
How many minutes did you usually spend doing vigorous activities on a typical DAY?	Quantity of minutes, “Do not know”, or “Prefer not to answer”

For peer review only

Supplementary Table 2. Spearman correlation between self-reported physical activity and accelerometer-measured physical activity, according to participant characteristics in UK Biobank men

	N Men (%)	Correlation	95% Confidence Interval
**Total**	35,955	0.24	0.23, 0.25
**Age group at recruitment (years)**			
<55 years	13,214 (36.8)	0.31	0.29, 0.32
55+ years	22,741 (63.3)	0.22	0.21, 0.23
**Socioeconomic status, fifths**			
Top fifth	7,584 (21.1)	0.23	0.21, 0.25
Bottom fifth	6,800 (18.9)	0.26	0.23, 0.28
**BMI (kg/m^2)**			
<25	10,590 (29.5)	0.27	0.25, 0.28
25-29.9	17,874 (49.7)	0.21	0.19, 0.22
>30	7,491 (20.8)	0.22	0.80, 0.24
**College or university degree**			
Yes	16,709 (46.5)	0.25	0.24, 0.27
No	19,246 (53.5)	0.24	0.22, 0.25
**Current employment status**			
In paid employment or self-employed	22,942 (63.8)	0.27	0.26, 0.28
Retired	11,361 (31.6)	0.24	0.22, 0.26
Other	1,652 (4.6)	0.30	0.26, 0.34
**Job involves mainly walking/standing**			
Never or rarely	9,825 (42.8)	0.29	0.27, 0.31
Sometimes	7,534 (32.9)	0.24	0.22, 0.26
Usually or Always	5,574 (24.3)	0.19	0.16, 0.21
**Job involves heavy manual work**			
Never, rarely	16,443 (71.7)	0.27	0.26, 0.29
Sometimes	4,160 (18.1)	0.17	0.14, 0.19
Usually or Always	2,335 (10.2)	0.12	0.08, 0.16
**Alcohol intake frequency**			
Weekly or more	29,421 (81.8)	0.23	0.22, 0.24
Less than weekly	6,530 (18.2)	0.28	0.26, 0.30
**Smoking status**			
Never	18,928 (52.6)	0.26	0.24, 0.27
Ever	16,964 (47.2)	0.22	0.21, 0.24
**Long-standing illness or disability**			
No	25,129 (69.9)	0.23	0.22, 0.24
Yes	10,825 (30.1)	0.25	0.23, 0.27

For peer review only

Supplementary Table 3. Spearman correlation between self-reported physical activity and accelerometer-measured physical activity, according to participant characteristics in UK Biobank women

	N Women (%)	Correlation	95% Confidence Interval
**Total**	42,992	0.22	0.21, 0.23
**Age group at recruitment (years)**			
<55 years	18,973 (44.1)	0.26	0.25, 0.28
55+ years	24,019 (55.9)	0.20	0.19, 0.22
**Socioeconomic status, fifths**			
Top fifth	8,401 (19.5)	0.22	0.30, 0.24
Bottom fifth	8,744 (20.3)	0.22	0.30, 0.24
**BMI (kg/m^2)**			
<25	20,255 (47.1)	0.21	0.20, 0.23
25-29.9	15,146 (35.2)	0.18	0.17, 0.20
>30	7,591 (17.7)	0.15	0.13, 0.17
**College or university degree**			
Yes	19,214 (44.7)	0.22	0.21, 0.24
No	23,778 (55.3)	0.22	0.20, 0.23
**Current employment status**			
In paid employment or self-employed	26,693 (62.1)	0.24	0.23, 0.25
Retired	12,710 (29.6)	0.22	0.20, 0.24
Other	3,589 (8.4)	0.30	0.27, 0.33
**Job involves mainly walking/standing**			
Never or rarely	12,191 (45.7)	0.25	0.23, 0.27
Sometimes	7,839 (29.4)	0.21	0.19, 0.23
Usually or Always	6,648 (24.9)	0.18	0.16, 0.20
**Job involves heavy manual work**			
Never, rarely	20,762 (77.8)	0.24	0.22, 0.25
Sometimes	4,353 (16.3)	0.17	0.14, 0.20
Usually or Always	1,567 (5.9)	0.13	0.08, 0.18
**Alcohol intake frequency**			
Weekly or more	29,829 (69.4)	0.22	0.21, 0.23
Less than weekly	13,152 (30.6)	0.21	0.20, 0.23
**Smoking status**			
Never	25,998 (60.5)	0.21	0.20, 0.22
Ever	16,936 (39.4)	0.23	0.22, 0.25
**Long-standing illness or disability**			
No	32,307 (75.2)	0.21	0.20, 0.22
Yes	10,685 (24.9)	0.23	0.21, 0.24

For peer review only

Supplementary Table 4. Association of self-reported and accelerometer-measured physical activity with measures of adiposity

	N	BMI (kg/m^2)	95% CI	Body fat percent	95% CI	Waist circumference (cm)	95% CI
**Men**							
**Questionnaire**							
Bottom tenth (least active)	3,643	0.00 (reference)		0.00 (reference)		0.00 (reference)	
2nd tenth	3,742	-0.69	-0.87, -0.52	-0.99	-1.23, -0.74	-1.97	-2.45, -1.50
3rd tenth	3,410	-0.91	-1.09, -0.73	-1.42	-1.68, -1.17	-2.89	-3.38, -2.41
4th tenth	3,677	-1.22	-1.40, -1.05	-1.97	-2.22, -1.72	-3.81	-4.29, -3.34
5th tenth	3,522	-1.32	-1.50, -1.14	-2.14	-2.39, -1.88	-4.47	-4.95, -3.99
6th tenth	3,584	-1.45	-1.63, -1.27	-2.50	-2.75, -2.24	-4.79	-5.27, -4.31
7th tenth	3,596	-1.59	-1.76, -1.41	-2.88	-3.13, -2.63	-5.41	-5.89, -4.93
8th tenth	3,591	-1.63	-1.81, -1.45	-3.09	-3.34, -2.83	-5.92	-6.40, -5.44
9th tenth	3,595	-1.71	-1.89, 1.53	-3.21	-3.46, -2.95	-6.33	-6.81, -5.85
Top tenth (most active)	3,595	-1.77	-1.95, -1.59	-3.56	-3.81, -3.31	-6.42	-6.90, -5.93
**Accelerometer**							
Bottom tenth (least active)	3,598	0.00 (reference)		0.00 (reference)		0.00 (reference)	
2nd tenth	3,604	-0.77	-0.95, -0.60	-0.99	-1.24, -0.75	-2.23	-2.70, -1.76
3rd tenth	3,592	-1.33	-1.50, -1.15	-1.83	-2.07, -1.58	-3.69	-4.16, -3.22
4th tenth	3,610	-1.60	-1.77, -1.42	-2.20	-2.45, -1.95	-4.59	-5.06, -4.12
5th tenth	3,597	-1.88	-2.06, -1.71	-2.69	-2.94, -2.44	-5.43	-5.90, -4.96
6th tenth	3,585	-2.07	-2.24, -1.89	-3.04	-3.28, -2.79	-6.00	-6.47, -5.53
7th tenth	3,589	-2.32	-2.49, -2.14	-3.52	-3.77, -3.27	-7.01	-7.49, -6.54
8th tenth	3,594	-2.57	-2.75, -2.39	-3.84	-4.09, -3.59	-7.69	-8.17, -7.22
9th tenth	3,591	-3.02	-3.20, -2.84	-4.75	-5.00, -4.50	-9.01	-9.48, -8.53
Top tenth (most active)	3,595	-3.61	-3.79, -3.43	-5.98	-6.24, -5.73	-11.23	-11.72, -10.75

For peer review only

**Women**							
**Questionnaire**							
Bottom tenth (least active)	4,433	0.00 (reference)		0.00 (reference)		0.00 (reference)	
2nd tenth	4,187	-0.81	-1.00, -0.61	-1.05	-1.33, -0.78	-1.77	-2.25, -1.29
3rd tenth	4,278	-1.37	-1.57, -1.18	-1.89	-2.16, -1.61	-3.33	-3.81, -2.86
4th tenth	4,318	-1.44	-1.64, -1.25	-2.16	-2.43, -1.88	-3.74	-4.22, -3.27
5th tenth	4,296	-1.60	-1.79, -1.40	-2.44	-2.72, -2.17	-3.92	-4.39, -3.44
6th tenth	4,308	-1.86	-2.05, -1.66	-2.89	-3.16, -2.61	-4.71	-5.18, -4.23
7th tenth	4,276	-1.98	-2.18, -1.79	-3.18	-3.45, -2.90	-5.02	-5.50, -4.55
8th tenth	4,300	-2.20	-2.39, -2.00	-3.57	-3.85, -3.30	-5.73	-6.20, -5.25
9th tenth	4,305	-2.28	-2.48, -2.09	-3.86	-4.14, -3.59	-5.91	-6.39, -5.44
Top tenth (most active)	4,291	-2.51	-2.70, -2.31	-4.25	-4.52, -3.97	-6.39	-6.87, -5.91
**Accelerometer**							
Bottom tenth (least active)	4,314	0.00 (reference)		0.00 (reference)		0.00 (reference)	
2nd tenth	4,292	-1.14	-1.33, -0.95	-1.43	-1.70, -1.16	-3.02	-3.49, -2.56
3rd tenth	4,293	-1.84	-2.03, -1.65	-2.35	-2.62, -2.09	-4.42	-4.89, -3.95
4th tenth	4,307	-2.31	-2.50, -2.12	-2.90	-3.17-2.63	-5.43	-5.90, -4.97
5th tenth	4,312	-2.60	-2.79, -2.41	-3.52	-3.79, -3.25	-6.27	-6.74, -5.80
6th tenth	4,286	-2.92	-3.11, -2.72	-4.05	-4.32, -3.79	-7.18	-7.65, -6.71
7th tenth	4,292	-3.22	-3.42, -3.04	-4.63	-4.90, -4.36	-7.88	-8.35, -7.41
8th tenth	4,301	-3.60	-3.80, -3.41	-5.34	-5.61, -5.07	-8.80	-9.27, -8.33
9th tenth	4,305	-4.17	-4.36, -3.98	-6.37	-6.64,-6.10	-10.12	-10.59, -9.64
Top tenth (most active)	4,290	-4.80	-4.99, -4.60	-8.06	-8.33, -7.78	-11.92	-12.39, -11.44

Analyses are adjusted for age, socioeconomic status, alcohol intake, smoking status, educational qualifications, and employment status. Analyses are further adjusted for parity and hormone replacement therapy use in women.

For peer review only

Supplementary Table 5. Association of accelerometer-measured physical activity with body fat percent, measured by bioelectrical impedance and dual energy X-ray absorptiometry (DXA) in UK Biobank

Accelerometer-measured physical activity	N	Mean body fat percent	95% CI	Coefficient	95% CI
**Men**		**Measured by impedance**			
Bottom fifth (least active)	237	27.4	26.7, 28.0	0.00 (reference group)	
2nd fifth	231	26.0	25.3, 26.6	-1.29	-2.21, -0.38
3rd fifth	229	24.7	24.0, 25.3	-2.63	-3.55, -1.71
4th fifth	253	23.8	23.1, 24.4	-3.46	-4.36, -2.56
Top fifth (most active)	235	21.8	21.1, 22.4	-5.51	-6.45, -4.58
**Men**		**Measured by DXA**			
Bottom fifth (least active)	237	34.1	33.3, 34.8	0.00 (reference group)	
2nd fifth	231	31.6	30.9, 32.4	-2.38	-3.45, -1.31
3rd fifth	229	30.0	29.2, 30.7	-4.12	-5.20, -3.05
4th fifth	253	28.7	28.0, 29.4	-5.48	-6.54, -4.42
Top fifth (most active)	235	26.1	25.4, 26.9	-8.18	-9.27, -7.08
**Women**		**Measured by impedance**			
Bottom fifth (least active)	270	38.4	37.6, 39.1	0.00 (reference group)	
2nd fifth	244	36.6	35.9, 37.4	-1.46	-2.55, -0.37
3rd fifth	265	36.1	35.4, 36.9	-1.93	-3.01, -0.85
4th fifth	228	34.5	33.7, 35.3	-3.43	-4.55, -2.31
Top fifth (most active)	265	30.8	30.1, 31.6	-6.97	-8.07, -5.87
**Women**		**Measured by DXA**			
Bottom fifth (least active)	270	42.5	41.7, 43.3	0.00 (reference group)	
2nd fifth	244	40.4	39.6, 41.2	-1.87	-3.04, -0.71
3rd fifth	265	39.8	39.0, 40.6	-2.55	-3.70, -1.40
4th fifth	228	37.1	36.2, 37.9	-4.98	-6.17, -3.78
Top fifth (most active)	265	33.3	32.5, 34.1	-8.83	-10.0, -7.65

Analyses are adjusted for age, socioeconomic status, alcohol intake, smoking status, educational qualifications, and employment status. Analyses are further adjusted for parity and hormone replacement therapy use in women.

For peer review only

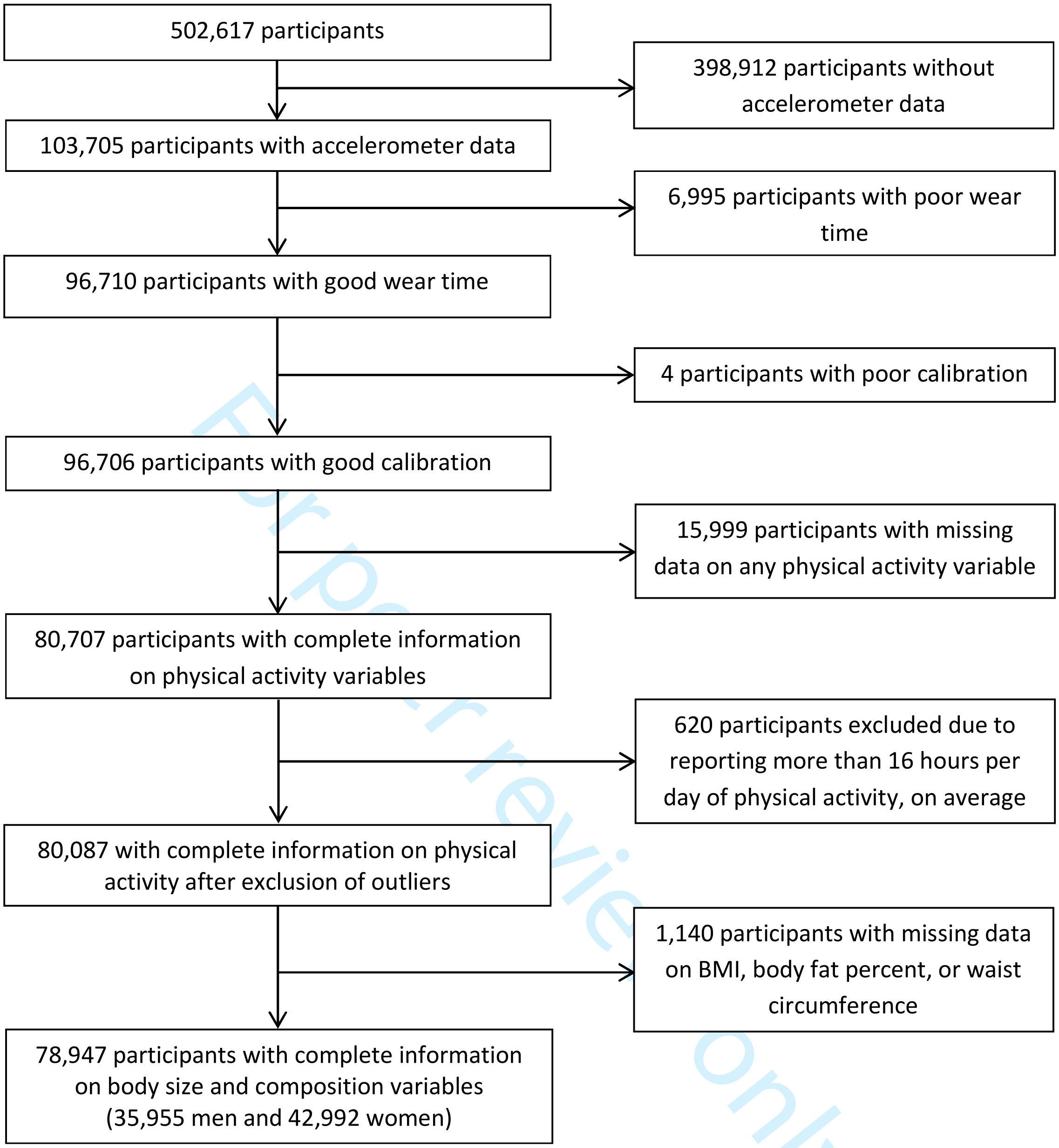

For peer review only

STROBE Statement—Checklist of items that should be included in reports of ***cross-sectional studies***

	Item No	Recommendation
**Title and abstract**	1	☑ (*a*) Indicate the study's design with a commonly used term in the title or the abstract
		☑ (*b*) Provide in the abstract an informative and balanced summary of what was done and what was found
**Introduction**		
Background/rationale	2	☑ Explain the scientific background and rationale for the investigation being reported
Objectives	3	☑ State specific objectives, including any prespecified hypotheses
**Methods**		
Study design	4	☑ Present key elements of study design early in the paper
Setting	5	☑ Describe the setting, locations, and relevant dates, including periods of recruitment, exposure, follow-up, and data collection
Participants	6	☑ (*a*) Give the eligibility criteria, and the sources and methods of selection of participants
Variables	7	☑ Clearly define all outcomes, exposures, predictors, potential confounders, and effect modifiers. Give diagnostic criteria, if applicable
Data sources/ measurement	8*	☑ For each variable of interest, give sources of data and details of methods of assessment (measurement). Describe comparability of assessment methods if there is more than one group
Bias	9	☑ Describe any efforts to address potential sources of bias
Study size	10	☑ Explain how the study size was arrived at
Quantitative variables	11	☑ Explain how quantitative variables were handled in the analyses. If applicable, describe which groupings were chosen and why
Statistical methods	12	☑ (*a*) Describe all statistical methods, including those used to control for confounding
		☑ (*b*) Describe any methods used to examine subgroups and interactions
		☑ (*c*) Explain how missing data were addressed
		N/A (*d*) If applicable, describe analytical methods taking account of sampling strategy
		☑ (*e*) Describe any sensitivity analyses
**Results**		
Participants	13*	☑ (a) Report numbers of individuals at each stage of study—eg numbers potentially eligible, examined for eligibility, confirmed eligible, included in the study, completing follow-up, and analysed
		☑ (b) Give reasons for non-participation at each stage
		☑ (c) Consider use of a flow diagram
Descriptive data	14*	☑ (a) Give characteristics of study participants (eg demographic, clinical, social) and information on exposures and potential confounders
		☑ (b) Indicate number of participants with missing data for each variable of interest
Outcome data	15*	☑ Report numbers of outcome events or summary measures
Main results	16	☑ (*a*) Give unadjusted estimates and, if applicable, confounder-adjusted estimates and their precision (eg, 95% confidence interval). Make clear which confounders were adjusted for and why they were included
		☑ (*b*) Report category boundaries when continuous variables were categorized

For peer review only - http://bmjopen.bmj.com/site/about/guidelines.xhtml

		N/A (*c*) If relevant, consider translating estimates of relative risk into absolute risk for a meaningful time period
Other analyses	17	☑ Report other analyses done—eg analyses of subgroups and interactions, and sensitivity analyses
**Discussion**		
Key results	18	☑ Summarise key results with reference to study objectives
Limitations	19	☑ Discuss limitations of the study, taking into account sources of potential bias or imprecision. Discuss both direction and magnitude of any potential bias
Interpretation	20	☑ Give a cautious overall interpretation of results considering objectives, limitations, multiplicity of analyses, results from similar studies, and other relevant evidence
Generalisability	21	☑ Discuss the generalisability (external validity) of the study results
**Other information**		
Funding	22	☑ Give the source of funding and the role of the funders for the present study and, if applicable, for the original study on which the present article is based

*Give information separately for exposed and unexposed groups.

Note: An Explanation and Elaboration article discusses each checklist item and gives methodological background and published examples of transparent reporting. The STROBE checklist is best used in conjunction with this article (freely available on the Web sites of PLoS Medicine at http://www.plosmedicine.org/, Annals of Internal Medicine at http://www.annals.org/, and Epidemiology at http://www.epidem.com/). Information on the STROBE Initiative is available at www.strobe-statement.org.

For peer review only

For peer review only - http://bmjopen.bmj.com/site/about/guidelines.xhtml

BMJ Open

Accelerometer compared with questionnaire measures of physical activity in relation to body size and composition: a large cross-sectional analysis of UK Biobank

Journal:	*BMJ Open*
Manuscript ID	bmjopen-2018-024206.R1
Article Type:	Research
Date Submitted by the Author:	23-Aug-2018
Complete List of Authors:	Guo, Wenji; University of Oxford, Nuffield Department of Population Health<br>Key, Timothy; University of Oxford, Nuffield Department of Population Health<br>Reeves, Gillian; University of Oxford, Nuffield Department of Population Health
**Primary Subject Heading**:	Epidemiology
Secondary Subject Heading:	Public health
Keywords:	EPIDEMIOLOGY, adiposity, physical activity, accelerometer, activity monitor

SCHOLARONE™
Manuscripts

Accelerometer compared with questionnaire measures of physical activity in relation to body size and composition: a large cross-sectional analysis of UK Biobank

Wenji Guo, Timothy J Key, Gillian K Reeves

Cancer Epidemiology Unit, Nuffield Department of Population Health, University of Oxford, Oxford, UK (WG, TJK, GKR)

Corresponding author:

Wenji Guo

Cancer Epidemiology Unit

Nuffield Department of Population Health

University of Oxford

Richard Doll Building

Roosevelt Drive

Oxford OX3 7LF, UK

Tel +44 1865 289635

wenji.guo@ndph.ox.ac.uk

wguo8@jhmi.edu

Abbreviations: body mass index (BMI), confidence interval (CI), dual-energy X-ray absorptiometry (DXA), International Physical Activity Questionnaire (IPAQ), metabolic equivalents (METs), standard deviation (SD)

For peer review only

Abstract

Objectives: Previous studies of the association between physical activity and adiposity are largely based on physical activity and body mass index (BMI) from questionnaires, which are prone to inaccurate and biased reporting. We assessed the associations of accelerometer-measured compared to questionnaire-measured physical activity with BMI, waist circumference and body fat percent measured by bioelectrical impedance and dual-energy X-ray absorptiometry (DXA).

Design: Cross-sectional analysis of UK Biobank participants

Setting: UK Biobank assessment centers

Participants: 78,947 UK Biobank participants (35,955 men and 42,992 women) aged 40-70 at recruitment, who had physical activity measured by both questionnaire and accelerometer.

Main outcome measures: BMI, waist circumference and body fat percent measured by bioelectrical impedance

Results: Correlation between accelerometer and questionnaire measures of physical activity, recorded approximately 5.5 years apart, was low overall and even lower in participants with higher BMI and in older participants. Greater physical activity was associated with lower adiposity. Women in the top tenth of accelerometer-measured physical activity had a 4.8 (95% CI: 4.6, 5.0) kg/m^2 lower BMI, 8.1% (95% CI: 7.8, 8.3) lower body fat percent, and 11.9 (95% CI 11.4, 12.4) cm lower waist circumference. Women in the top tenth of questionnaire-measured physical activity had a 2.5 (95% CI: 2.3, 2.7) kg/m^2 lower BMI, 4.3% (95% CI: 4.0, 4.5) lower body fat percent, and 6.4 (95% CI: 5.9, 6.9) cm lower waist circumference, compared to women in the bottom tenths. The patterns were similar in men and also similar with body fat percent measured by DXA compared to impedance.

For peer review only - http://bmjopen.bmj.com/site/about/guidelines.xhtml

For peer review only

Conclusion: Our findings of approximately twofold stronger associations between physical activity and adiposity with objectively-measured compared to self-reported physical activity demonstrate substantial measurement error in self-reported physical activity, especially among participants with higher BMI and among older participants, and further emphasizes the need to incorporate objective measures in future studies.

Strengths and limitations of this study:

- This study utilizes data on physical activity objectively measured by accelerometer rather than only self-reported data from questionnaires, which are prone to inaccurate and potentially biased reporting.
- This study is by far the largest study to compare associations between physical activity objectively measured by accelerometer and self-reported physical activity in relation to various measures of adiposity, including body fat percent assessed by bioelectrical impedance and dual energy x-ray absorptiometry.
- Due to the cross-sectional nature of this study, we cannot assess to what extent physical activity is causally related to adiposity.

For peer review only

INTRODUCTION

The prevalence of overweight and obesity is high worldwide and is associated with increased risk of various conditions including heart disease, stroke, hypertension, diabetes, and some cancers (1, 2). Although physical activity is generally accepted to be important for prevention of weight gain, achievement of modest weight loss, and prevention of weight regain after weight loss (3), randomized controlled trials have shown inconsistent results, perhaps partly due to limited duration of interventions and difficulty in long-term adherence to exercise regimens (4), and previous large-scale observational studies are mostly based on self-reported physical activity from questionnaires, which are prone to both inaccurate reporting and reporting bias (5).

Prior studies have demonstrated low to moderate correlation between self-reported and objective accelerometer measures of physical activity (6, 7). Self-reported and accelerometer-measured physical activity capture different aspects of physical activity with limitations unique to each (7). However, research methods utilizing more objective measures of physical activity, along with more detailed measures of body fat, are needed to reduce measurement error and more accurately characterize the association between physical activity and adiposity.

We examined the association between physical activity and adiposity, with accelerometer-measured compared to self-reported physical activity in nearly 80,000 participants. These associations were assessed using various measures of adiposity, including BMI, waist circumference, and body fat percent measured by both bioelectrical impedance and dual-energy X-ray absorptiometry (DXA). We also explored how the associations vary by age.

For peer review only - http://bmjopen.bmj.com/site/about/guidelines.xhtml

METHODS

Data source

Data were obtained from UK Biobank. Details of UK Biobank design, rationale, and survey methods have been described elsewhere (8). Information on data available and access procedures are described on the study website (http://www.ukbiobank.ac.uk/). UK Biobank has approval from the National Information Governance Board for Health & Social Care in England and Wales, the North West Multi-centre Research Ethics Committee, and the Community Health Index Advisory Group in Scotland. Written informed consent was provided by all participants.

Study participants

The complete UK Biobank dataset includes 502,617 UK adults (229,164 men and 273,453 women) between 40 to 70 years of age at recruitment during 2006 to 2010. During the baseline assessment center visit, participants completed a touchscreen questionnaire which included questions on socio-demographics, lifestyle, health and medical history, and sex-specific factors. The present study was restricted to participants with available accelerometer data (n=103,705). Participants were excluded if they did not have at least 72 hours of data and also data in each one-hour period of the 24-hour cycle across multiple days (n=6,995). Participants were also excluded if they had insufficient data for calibration (n=4). Participants who had missing data on any of the physical activity variables used in our analyses were excluded (n=15,999). Participants who reported physical activity greater than an average of 16 hours per day (n=620) were also excluded as recommended by the International Physical Activity Questionnaire (IPAQ) scoring guidelines, which can be accessed at file:///H:/Downloads/GuidelinesforDataProcessingandAnalysisoftheInternationalPhysicalActivityQuestionnaireIPAQShortandLongForms.pdf. Finally, participants with missing data on

For peer review only - http://bmjopen.bmj.com/site/about/guidelines.xhtml

BMI (n=146), body fat percent (n=988), and waist circumference (n=6) were excluded. The analyses included 35,955 men and 42,992 women (**Supplementary Figure 1**).

Self-reported physical activity

Physical activity questions from the baseline questionnaire captured the frequency and duration of three intensities of activity (walking, moderate, and vigorous). Participants were asked how many days per week they typically engaged in each category of activity. For each category in which an answer of one or more days was given, the participant was subsequently asked the number of minutes on average spent on the activity per day. Questions were adapted from the IPAQ, a validated survey instrument (9), and are listed in **Supplementary Table 1**. Metabolic equivalents (METs) were used to quantify physical activity; 1 MET is expended by sitting quietly for 1 hour, and the MET value reflects the ratio of energy expended per kilogram of body weight per hour to that expended when sitting quietly (10). The number of minutes per day engaged in each level of activity was multiplied by the respective MET score for the corresponding level of activity (3.3 for walking, 4.0 for moderate physical activity, and 8.0 for vigorous physical activity) (11). MET minutes per day were converted to MET hours per week. The total amount of METs was calculated by summing total METs from the walking, moderate, and vigorous activity levels. Following IPAQ scoring guidelines, physical activity of less than 10 minutes per day for any category was recoded to 0.

Accelerometer-measured physical activity

A total of 236,519 participants, all of whom had provided a valid email address, were invited to participate in a seven day accelerometer study between February 2013 and December 2015 (on average, approximately 5.5 years after recruitment when baseline physical activity was self-reported). Starting in June 2013, participants were sent wrist-worn

For peer review only - http://bmjopen.bmj.com/site/about/guidelines.xhtml

triaxial accelerometers (Axivity AX3, Newcastle upon Tyne, UK) that were programmed to capture three-dimensional acceleration data at 100 Hz with a dynamic range of ±8 g. Participants were also given instructions to wear the accelerometer on their dominant wrist continuously for seven days and then to send the device to the coordinating center using the provided prepaid envelope. Further details on data collection, processing, and analysis can be found elsewhere (12). We used the "overall acceleration average" variable (data field 90012) in the present analyses.

Anthropometry and body composition

At the UK Biobank baseline interview, trained staff measured standing height using the Seca 202 device (Seca, Hamburg, Germany). BMI was calculated by dividing weight (kg) by the square of standing height (m^2). The Wessex non-stretchable sprung tape measure (Wessex, United Kingdom) was used to measure waist circumference at the level of the umbilicus. The Tanita BC-418MA body composition analyzer (Tanita, Tokyo, Japan) was used to measure body fat percent using bioelectrical impedance. DXA was used to measure fat percent on a subset of 2,457 participants included in the present study, beginning in 2014 using the GE-Lunar iDXA (GE Healthcare, Chicago, USA).

Statistical analyses

Baseline characteristics were summarized by physical activity (least active fifth, most active fifth, and overall) separately for men and women. Since self-reported physical activity was not normally distributed, Spearman's correlation coefficients were used to measure the strength of correlations between self-reported and accelerometer-measured physical activity in the overall population and in subgroups based on sociodemographic characteristics.

For peer review only - http://bmjopen.bmj.com/site/about/guidelines.xhtml

Self-reported and accelerometer-measured physical activity were categorized into tenths and the median value within each category of physical activity is shown in the figures. The associations of physical activity and adiposity measures were examined using multivariable linear regression, separately in men and women. Analyses comparing the association of accelerometer-measured physical activity with body fat percent, measured by bioelectrical impedance and DXA were restricted to participants with both measures. Likelihood ratio tests were used to assess whether the associations between physical activity and adiposity were modified by age (<55 years or 55+ years), separately for self-reported and accelerometer-measured physical activity.

Covariates were determined a priori and were 5-year age at recruitment categories, socioeconomic status as indicated by fifths of Townsend deprivation index (13), educational qualifications, employment status, smoking status (never, previous, current), and alcohol intake frequency. Analyses in women were additionally adjusted for parity (nulliparous, 1, 2, 3, 4 or more births) and hormone replacement therapy use (never, previous, current). As a covariate, educational qualification was grouped into the following categories: vocational qualifications, national exams at age 16 (O levels, GCSEs, CSEs, or equivalent), optional national exams at ages 17-18 years (A levels, AS levels, or equivalent), and college or university degree. Employment status was categorized as paid or self-employed, retired, looking after home and/or family, unemployed, doing unpaid or voluntary work, unable to work due to sickness or disability, and student. Alcohol intake was categorized as never, special occasions only, 1-3 times a month, 1-2 times a week, 3-4 times a week, and daily or almost daily.

Missing data were grouped in a separate unknown category for each covariate. There were less than 1% missing data for all covariates except for educational qualifications (7.4% missing data). To assess the impact of missing values, a sensitivity analysis restricted to

For peer review only
For peer review only - http://bmjopen.bmj.com/site/about/guidelines.xhtml

participants with known values for all covariates was conducted. We also conducted sensitivity analyses to assess the impact of excluding participants who reported long-term illness, disability or infirmity, participants who reported fair or poor health rather than excellent or good health, and participants whose jobs usually or always required heavy manual work. Analyses were conducted using STATA, version 15.0 (Stata Corp LP, College Station, TX).

Patient and public involvement

This study did not involve patients and the public.

RESULTS

Characteristics of the study population by least active and most active fifth of accelerometer-measured physical activity are shown in **Table 1**.

Table 1. Characteristics of the UK Biobank study population, according to fifths of accelerometer-measured physical activity.

	Least active men	Most active men	All men		Least active women	Most active women	All women
	<20.8 milli-gravity	≥33.7 milli-gravity			<22.2 milli-gravity	≥34.6 milli-gravity	
**Number of participants**	7,202	7,186	35,955		8,606	8,595	42,992
**Age at recruitment (years), mean (SD)**	59.7 (7.0)	53.4 (7.7)	56.7 (7.9)		58.0 (7.4)	52.6 (7.4)	55.3 (7.7)
**Lowest fifth of socioeconomic status**	1,520 (21.1%)	1,351 (18.8%)	6,800 (18.9%)		1,897 (22.0%)	1,699 (19.8%)	8,744 (20.3%)
**Accelerometer-measured physical activity (milli-gravity), mean (SD)**	17.5 (2.6)	40.5 (7.8)	27.6 (8.7)		18.9 (2.7)	40.6 (6.3)	28.7 (8.0)
**Self-reported physical activity (MET-hours/week), median (IQR)**	20.7 (9.0, 42.6)	44.2 (23.7, 80.9)	29.9 (14.2, 58.1)		21.3 (9.9, 41.7)	40.2 (21.8, 73.2)	29.3 (14.4, 55.3)
**Height (cm), mean (SD)**	176.3 (6.8)	176.4 (6.6)	176.5 (6.6)		163.2 (6.3)	163.7 (6.1)	163.5 (6.2)
**Weight (kg), mean (SD)**	89.4 (15.4)	80.8 (11.4)	84.9 (13.5)		75.5 (15.6)	65.0 (10.3)	69.9 (13.2)
**BMI (kg/m^2), mean (SD)**	28.8 (4.6)	25.9 (3.3)	27.2 (4.0)		28.3 (5.7)	24.3 (3.7)	26.2 (4.8)
**Body fat percent (%) [a], mean (SD)**	27.0 (5.6)	21.7 (5.4)	24.4 (5.7)		38.7 (6.6)	31.7 (6.4)	35.3 (6.8)
**Waist circumference (cm), mean (SD)**	100.1 (11.7)	90.9 (9.3)	95.4 (10.8)		87.9 (13.3)	77.6 (9.5)	82.4 (11.7)
**College or university**	3,018 (41.9%)	3,365	16,709		3,586 (41.7%)	4,060	19,214

For peer review only

For peer review only - http://bmjopen.bmj.com/site/about/guidelines.xhtml

**degree**		(46.8%)	(46.5%)			(47.2%)	(44.7%)
**Current employment status**							
Paid employment/self-employed	3,608 (50.1%)	5,420 (75.4%)	22,942 (63.8%)		4,401 (51.1%)	6,101 (71.0%)	26,693 (62.1%)
Retired	3,107 (43.1%)	1,451 (20.2%)	11,361 (31.6%)		3,517 (40.9%)	1,591 (18.5%)	12,710 (29.6%)
Other	487 (6.8%)	315 (4.4%)	1,652 (4.6%)		688 (8.0%)	903 (10.5%)	3,589 (8.3%)
**Job involves mainly walking/standing** [b]	707 (19.6%)	1,742 (32.1%)	5,574 (24.3%)		893 (20.3%)	1,926 (31.6%)	6,648 (24.9%)
**Job involves heavy manual work** [c]	272 (7.5%)	912 (16.8%)	2,335 (10.2%)		170 (3.9%)	576 (9.4%)	1,567 (5.9%)
**Weekly or more frequent alcohol intake**	5,545 (77.0%)	5,989 (83.3%)	29,421 (81.8%)		5,295 (61.5%)	6,292 (73.2%)	29,829 (69.4%)
**Ever smoker**	3,801 (52.8%)	3,126 (43.5%)	16,964 (47.2%)		3,583 (41.6%)	3,212 (37.4%)	16,936 (39.4%)
**Long-standing illness or disability**	3,089 (42.9%)	1,543 (21.5%)	10,825 (30.1%)		3,145 (36.5%)	1,449 (16.9%)	1,0685 (24.9%)

[a] Body fat percent was measured by bioelectrical impedance
[b] Participants who reported their work “usually” or “always” involved walking or standing for most of the time
[c] Participants who reported their work “usually” or “always” involved heavy manual or physical work for most of the time

For peer review only

Mean accelerometer-measured physical activity was 27.6 (standard deviation [SD] 8.7) milli-gravity in men and 28.7 (SD 8.0) milli-gravity in women. The most active participants were on average younger and had lower values for all body size and composition variables. They were more likely to have a college or university degree, be employed rather than retired, have an active job, and consume alcohol at least weekly. The least active participants were more likely to be ever smokers and were also more likely to have a long-standing illness or disability. The correlation between questionnaire and accelerometer-measured physical activity, recorded on average 5.5 years later, was 0.24 (95% confidence interval [CI]: 0.23, 0.25) in men (**Supplementary Table 2**) and 0.22 (95% CI: 0.21, 0.23) in women (**Supplementary Table 3**). The correlations were comparatively higher in participants who were younger and in participants who had lower BMI. The correlations were lower among

For peer review only - http://bmjopen.bmj.com/site/about/guidelines.xhtml

participants who reported that their job usually or always involved heavy manual work and/or mainly walking or standing.

The inverse associations between physical activity and all measures of adiposity were linear and approximately twofold larger in models that used accelerometer-measured rather than self-reported physical activity. Since there was heterogeneity in the associations between both self-reported and accelerometer-measured physical activity and adiposity by sex (P<0.001), separate analyses were performed in men and women. The mean differences in BMI and body fat percent were greater in women compared to men. Comparing the top to bottom tenth of accelerometer-measured physical activity, the difference in BMI was 4.8 (95% CI: 4.6, 5.0) kg/m^2 in women and 3.6 (95% CI: 3.4, 3.8) kg/m^2 in men (**Figure 1, Supplementary Table 4**).

Women in the top tenth of accelerometer-measured physical activity had an 8.1% (95% CI: 7.8, 8.3) lower body fat percent while women in the top tenth of self-reported physical activity had a 4.3% (95% CI: 4.0, 4.5) lower body fat percent, compared to those in the bottom tenth of physical activity. Men in the top tenth of accelerometer-measured physical activity had a 6.0% (95% CI: 5.7, 6.2) lower body fat percent while men in the top tenth of self-reported physical activity had a 3.6% (95% CI: 3.3, 3.8) lower body fat percent, compared to those in the bottom tenth (**Figure 1, Supplementary Table 4**).

Associations between physical activity and waist circumference were of similar magnitude in men and women, with an approximately twofold larger inverse association between waist circumference and physical activity when measured by accelerometer rather than questionnaire (**Figure 1, Supplementary Table 4**).

The results of sensitivity analyses excluding participants who had any missing values, reported a long-term illness or disability, reported a health rating worse than "good", or

For peer review only - http://bmjopen.bmj.com/site/about/guidelines.xhtml

For peer review only

whose jobs usually or always required heavy manual work did not materially differ from the main findings.

Figure 2 and Supplementary Table 5 show the associations between accelerometer-measured physical activity and bioelectrical impedance-measured body fat percent at baseline (2006-2010) compared to body fat percent measured by DXA starting in May 2014. Body fat percent by impedance at baseline was lower than body fat percent by DXA, measured on average seven years later. For both measures, there was a linear dose-response association between physical activity and body fat percent in both men and women. The inverse associations were stronger when body fat percent was measured by DXA. Compared to the least active women, the most active women had an 8.8% (95% CI: 7.7, 10.0) lower DXA-measured body fat percent and a 7.0% (95% CI: 5.9, 8.1) lower impedance-measured body fat percent (**Figure 2 and Supplementary Table 5**).

Associations between physical activity and measures of adiposity by age group are shown in **Figure 3** for men and **Figure 4** for women. For a given level of accelerometer-measured physical activity, the older participants (over age 55) had a slightly lower BMI but a higher body fat percent compared to their younger counterparts. For women, there was heterogeneity by age in the association between self-reported physical activity and body fat percent (P=0.03) but there was no heterogeneity by age when physical activity was measured by accelerometer (P=0.27).

DISCUSSION

In this large cross-sectional study of nearly 80,000 participants, we found that associations between physical activity and BMI, body fat percent, and waist circumference were stronger when physical activity was measured by accelerometer compared to questionnaire self-reports. Body fat percent measured by DXA at follow-up showed a slightly stronger

For peer review only - http://bmjopen.bmj.com/site/about/guidelines.xhtml

association with physical activity compared to body fat percent measured by bioelectrical impedance at baseline, but the overall pattern of association was similar. The correlation between accelerometer-measured and self-reported physical activity, recorded 5.5 years apart, was lower in participants with higher BMI and in older participants.

There was a consistent dose-response relationship between physical activity and adiposity across the different measures of adiposity, which are highly correlated (14). Our analyses based on accelerometer-measured physical activity suggest an approximately linear inverse association between physical activity and adiposity, with the most active participants having the lowest BMI, body fat percent, and waist circumference. In contrast, the analyses in the same participants based on self-reported physical activity suggest a comparatively small further benefit of physical activity greater than 50 MET-hours a week on adiposity.

We have previously suggested that the steeper relationship between physical activity and lower adiposity within the lower range of physical activity could be due to either a comparatively larger benefit of physical activity for those who are relatively inactive or measurement error from over-reporting of high physical activity (14). The present analyses demonstrating an approximately linear dose-response relationship between accelerometer-measured physical activity and adiposity supports the latter explanation and further suggests that over-reporting of total physical activity contributed to the low overall correlation between self-reported and accelerometer-measured physical activity, although the time lag between these two measurements of physical activity may have also contributed to a low overall correlation coefficient. Wrist accelerometer-measured physical activity also has limitations, such as measuring movement of only one part of the body and the inability to reliably capture activities such as cycling (7), it has the major advantage of eliminating both inaccurate reporting that leads to random error as well as reporting bias that may vary by sociodemographic characteristics.

For peer review only - http://bmjopen.bmj.com/site/about/guidelines.xhtml

Measurement error in the self-reported data results in misclassification of individuals by physical activity status. We used the IPAQ short form data processing rules since the UK Biobank questionnaire did not comprehensively cover domain-specific activities, but it is still likely that lower intensity activities were underreported and reported less accurately (15). In contrast, the accelerometers were worn for 24 hours a day, over 7 days. Therefore, the lower correlation between self-reported and accelerometer-measured physical activity in older participants (16) and the heterogeneity by age seen only with the self-reported data may be explained by the observation that, in older adults, a greater proportion of physical activity is of lower intensity (17).

Individuals with higher body fat percent may report moderate and strenuous physical activity less accurately than leaner individuals, based on comparisons between self-reported physical activity and energy expenditure estimated from whole-room indirect calorimeter (18). In agreement with some previous studies, we found that the correlation between physical activity measured by questionnaire and accelerometer-measured physical activity was greater for those with lower BMI (7). This suggests that measurement error of self-reported physical activity may be greater in overweight and obese BMI groups.

We, like several prior studies, found stronger associations between accelerometer-measured physical activity and all measures of adiposity in women compared to men (19–21). This may partly be due to the fact that, in the present study, men were on average objectively less physically active than women. Differences in fat metabolism may also play a role, with a greater proportion of energy derived from lipolysis during exercise in women compared to men (21, 22).

To our knowledge, the present study is the largest to date comparing accelerometer-measured and self-reported physical activity in relation to direct measures of body fat,

For peer review only - http://bmjopen.bmj.com/site/about/guidelines.xhtml

although our results are consistent with prior, smaller studies that suggest a stronger association between adiposity and accelerometer-measured compared to self-reported physical activity (18, 20, 23–26). This study was population-based and recruited from 22 regions throughout the UK (27). A major strength of this study is the availability of both accelerometer-measured physical activity and body fat by impedance in nearly 80,000 participants, together with data on body fat assessed by DXA in over 2,400 participants. Additionally, the accelerometers used in this study were waterproof (12), overcoming a limitation of prior studies where the devices had to be removed for water-based activities (21).

While self-reported physical activity was available at baseline in these data, accelerometer-measured physical activity was assessed only 3-5 years after end of recruitment, which raises the question of whether higher adiposity at baseline predicts lower physical activity levels (28) rather than physical activity determining adiposity. However, our analysis of accelerometer-measured physical activity in relation to DXA-measured body fat percent, which was assessed within the same time frame as accelerometer-measured physical activity, showed similar results to the main analysis based on body fat percent assessed by impedance at baseline. The accelerometer-measured physical activity variable available in UK Biobank at the time of these analyses cannot be directly compared to MET hours of self-reported physical activity. However, Willetts et al. have recently developed physical activity phenotypes using a machine learning model with reference behaviors provided by data from a subset of participants who wore a camera along with the accelerometer (29). Once these variables are made publicly available in UK Biobank, research using these metrics will facilitate the translation of study results into public health messages.

Other limitations include the lack of data on total energy intake for the whole cohort. Although accelerometer-determined physical activity is positively associated with percent of

For peer review only - http://bmjopen.bmj.com/site/about/guidelines.xhtml

For peer review only

lean muscle mass (30), we did not consider this as a confounder in these analyses since we utilized data on direct measures of body fat percent. Since accelerometer-measured time spent in sedentary activity was not available, we did not conduct analyses on sedentary activity. Due to the cross-sectional nature of this study, we cannot assess to what extent physical activity is causally related to adiposity. Highly active individuals may also be more likely to maintain appropriate target dietary energy intake, for example. Although the UK Biobank cohort is not representative of the general population in the UK, results of associations between exposures and health outcomes may be generalizable and would not necessarily require the study population to be representative if the biological basis of the exposure-disease relationship is shared.

In conclusion, our findings based on objective accelerometer data indicate a stronger relationship between physical activity and adiposity than previously thought. Comparison of estimates of physical activity measured by questionnaire and by accelerometer suggest substantial measurement error in self-reported physical activity, emphasizing the need to incorporate objective measures of physical activity in future studies.

Funding: This work was supported by Cancer Research UK, grant number C570/A16491. WG is supported by the Clarendon Fund.

Disclosures: The authors have declared no conflicts of interest.

Contributors: WG, TJK, and GKR were responsible for study concept, design of the study, interpretation of the data, and manuscript writing. WG had primary responsibility for statistical analysis and final content. All authors reviewed and approved the final manuscript.

Dating sharing statement: No additional data are available.

For peer review only - http://bmjopen.bmj.com/site/about/guidelines.xhtml

REFERENCES

1. Must A, Spadano J, Coakley EH, et al. The disease burden associated with overweight and obesity. *JAMA*. 1999;282:1523–9.

2. Afshin A, Forouzanfar MH, Reitsma MB, et al. Health effects of overweight and obesity in 195 countries over 25 years. *N Engl J Med*. 2017;377:13–27.

3. Donnelly JE, Blair SN, Jakicic JM, et al. American College of Sports Medicine position stand. Appropriate physical activity intervention strategies for weight loss and prevention of weight regain for adults. *Med Sci Sport Exerc*. 2009;41:459–71.

4. Catenacci VA, Wyatt HR. The role of physical activity in producing and maintaining weight loss. *Nat Clin Pr End Met*. 2007;3:518–29.

5. Ainsworth BE, Caspersen CJ, Matthews CE, et al. Recommendations to improve the accuracy of estimates of physical activity derived from self report. *J Phys Act Heal*. 2012;9 Suppl 1:S76-84.

6. Koolhaas CM, van Rooij FJ, Cepeda M, et al. Physical activity derived from questionnaires and wrist-worn accelerometers: comparability and the role of demographic, lifestyle, and health factors among a population-based sample of older adults. *Clin Epidemiol*. 2018;10:1–16.

7. Skender S, Ose J, Chang-Claude J, *et al.* Accelerometry and physical activity questionnaires - a systematic review. *BMC Public Health*. 2016;16:515.

8. UK Biobank: Protocol for a large-scale prospective epidemiological resource. 2010. Available from: http://www.ukbiobank.ac.uk/wp-content/uploads/2011/11/UK-Biobank-Protocol.pdf

9. Craig CL, Marshall AL, Sjostrom M, et al. International physical activity questionnaire: 12-country reliability and validity. *Med Sci Sport Exerc*. 2003;35:1381–95.

For peer review only

10. Ainsworth BE, Haskell WL, Herrmann SD, et al. 2011 Compendium of physical activities: a second update of codes and MET values. *Med Sci Sport Exerc.* 2011;43:1575–81.

11. IPAQ Research Committee. Guidelines for data processing and analysis of the International Physical Activity Questionnaire (IPAQ) - short and long forms. 2005 [cited 2018 May 10]. Available from: https://sites.google.com/site/theipaq/scoring-protocol

12. Doherty A, Jackson D, Hammerla N, Plotz T, Olivier P, Granat MH, et al. Large scale population assessment of physical activity using wrist worn accelerometers: the UK Biobank Study. *PLoS One*. 2017;12:e0169649.

13. Morris R, Carstairs V. Which deprivation? A comparison of selected deprivation indexes. *J Public Heal Med*. 1991;13:318–26.

14. Guo W, Bradbury KE, Reeves GK, Key TJ. Physical activity in relation to body size and composition in women in UK Biobank. *Ann Epidemiol*. 2015;25:406–413 e6.

15. Sallis JF, Saelens BE. Assessment of physical activity by self-report: status, limitations, and future directions. *Res Q Exerc Sport*. 2000;71.

16. Dyrstad SM, Hansen BH, Holme IM, Anderssen SA. Comparison of self-reported versus accelerometer-measured physical activity. *Med Sci Sports Exerc*. 2014 Jan;46(1):99–106.

17. Westerterp KR. Physical activity as determinant of daily energy expenditure. *Physiol Behav*. 2008;93:1039–43.

18. Buchowski MS, Townsend KM, Chen KY, et al. Energy expenditure determined by self-reported physical activity is related to body fatness. *Obes Res*. 1999;7.

19. Sabia S, Cogranne P, van Hees VT, et al. Physical activity and adiposity markers at older ages: accelerometer vs questionnaire data. *J Am Med Dir Assoc*. 2015;16:438.e7-

For peer review only - http://bmjopen.bmj.com/site/about/guidelines.xhtml

13.

20. Bann D, Kuh D, Wills AK, et al. Physical activity across adulthood in relation to fat and lean body mass in early old age: findings from the Medical Research Council National Survey of Health and Development, 1946-2010. *Am J Epidemiol*. 2014;179:1197–207.

21. Wanner M, Richard A, Martin B, et al. Associations between self-reported and objectively measured physical activity, sedentary behavior and overweight/obesity in NHANES 2003-2006. *Int J Obes*. 2017;41:186–93.

22. Blaak E. Gender differences in fat metabolism. *Curr Opin Clin Nutr Metab Care*. 2001;4:499–502.

23. Hedrington MS, Davis SN. Sexual dimorphism in glucose and lipid metabolism during fasting, hypoglycemia, and exercise. *Front Endocrinol*. 2015;6:61.

24. Foong YC, Aitken D, Winzenberg T, Otahal P, Scott D, Jones G. The association between physical activity and reduced body fat lessens with age - results from a cross-sectional study in community-dwelling older adults. *Exp Gerontol*. 2014;55:107–12.

25. Harris TJ, Owen CG, Victor CR, et al. A comparison of questionnaire, accelerometer, and pedometer: measures in older people. *Med Sci Sport Exerc*. 2009;41:1392–402.

26. Atienza AA, Moser RP, Perna F, et al. Self-reported and objectively measured activity related to biomarkers using NHANES. *Med Sci Sport Exerc*. 2011;43:815–21.

27. Sudlow C, Gallacher J, Allen N, et al. UK Biobank: An Open Access Resource for Identifying the Causes of a Wide Range of Complex Diseases of Middle and Old Age. *PLoS Med*. 2015 Mar;12(3):e1001779.

28. Kim Y, White T, Wijndaele K, et al. Adiposity and grip strength as long-term predictors of objectively measured physical activity in 93 015 adults: the UK Biobank study. *Int J Obes*. 2017;41:1361–8.

For peer review only - http://bmjopen.bmj.com/site/about/guidelines.xhtml

29. Willetts M, Hollowell S, Aslett L, et al. Statistical machine learning of sleep and physical activity phenotypes from sensor data in 96,220 UK Biobank participants. *Sci Rep*. 2018;8(1):7961.

30. Foong YC, Chherawala N, Aitken D, et al. Accelerometer-determined physical activity, muscle mass, and leg strength in community-dwelling older adults. *J Cachexia Sarcopenia Muscle*. 2016 Jun;7(3):275–83.

For peer review only

Figure legends

Figure 1. Association of self-reported and accelerometer-measured physical activity with adiposity variables in UK Biobank

Association of A) accelerometer-measured and B) self-reported physical activity with BMI

Association of C) accelerometer-measured and D) self-reported physical activity with body fat percent

Association of E) accelerometer-measured and F) self-reported physical activity with waist circumference

Physical activity was grouped into tenths, separately in men and women.

Adjusted geometric means (from linear regression models) for BMI, body fat percent, and waist circumference are plotted against the median value within each tenth of self-reported or accelerometer-measured physical activity. Adjusted geometric means are represented by squares for men and triangles for women.

These analyses are stratified by age at recruitment, region of recruitment, and socioeconomic status, and are adjusted for educational qualifications, employment status, smoking status, and alcohol intake frequency. Analyses in women are additionally adjusted for parity and hormone replacement therapy use.

The figure shows point estimates and 95% confidence intervals.

Abbreviations: BMI, body mass index; MET, metabolic equivalent

Figure 2. Association of accelerometer-measured physical activity with body fat percent measured by impedance and DXA in UK Biobank A) men (n=1,185) and B) women (n=1,272)

For peer review only

For peer review only - http://bmjopen.bmj.com/site/about/guidelines.xhtml

Physical activity was grouped into fifths, separately in men and women.

Adjusted geometric means (from linear regression models) for body fat percent are plotted against the median value within each fifth of accelerometer-measured physical activity. Adjusted geometric means are represented by diamonds for body fat percent measured by impedance and circles for body fat percent measured by DXA.

These analyses are restricted to participants with measures of body fat percent by both impedance and DXA. Analyses are stratified by age at recruitment, region of recruitment, and socioeconomic status, and are adjusted for educational qualifications, employment status, smoking status, and alcohol intake frequency. Analyses in women are additionally adjusted for parity and hormone replacement therapy use.

The figure shows point estimates and 95% confidence intervals.

Abbreviations: DXA, dual-energy X-ray absorptiometry

Figure 3. Association of self-reported and accelerometer-measured physical activity with adiposity variables by age group in UK Biobank men

Association of physical activity measured by A) accelerometer and B) self-reported questionnaire with BMI.

Association of physical activity measured by C) accelerometer and D) self-reported questionnaire with body fat percent.

Physical activity was grouped into tenths.

Adjusted geometric means (from linear regression models) for BMI and body fat percent are plotted against the median value within each tenth of self-reported or accelerometer-

For peer review only - http://bmjopen.bmj.com/site/about/guidelines.xhtml

measured physical activity. Adjusted geometric means are represented by diamonds for those under age 55 and squares for those ages 55 or older.

These analyses are stratified by age at recruitment, region of recruitment, and socioeconomic status, and are adjusted for educational qualifications, employment status, smoking status, and alcohol intake frequency.

The figure shows point estimates and 95% confidence intervals.

Abbreviations: BMI, body mass index; MET, metabolic equivalent

Figure 4. Association of self-reported and accelerometer-measured physical activity with adiposity variables by age group in UK Biobank women

Association of physical activity measured by A) accelerometer and B) self-reported questionnaire with BMI.

Association of physical activity measured by C) accelerometer and D) self-reported questionnaire with body fat percent.

Physical activity was grouped into tenths.

Adjusted geometric means (from linear regression models) for BMI and body fat percent are plotted against the median value within each tenth of self-reported or accelerometer-measured physical activity. Adjusted geometric means are represented by diamonds for those under age 55 and squares for those ages 55 or older.

These analyses are stratified by age at recruitment, region of recruitment, and socioeconomic status, and are adjusted for educational qualifications, employment status, smoking status, and alcohol intake frequency, parity, and hormone replacement therapy use.

For peer review only - http://bmjopen.bmj.com/site/about/guidelines.xhtml

The figure shows point estimates and 95% confidence intervals.

Abbreviations: BMI, body mass index; MET, metabolic equivalent

Supplementary Figure 1. Flowchart illustrating the application of exclusion criteria for the current study in UK Biobank

For peer review only

For peer review only - http://bmjopen.bmj.com/site/about/guidelines.xhtml

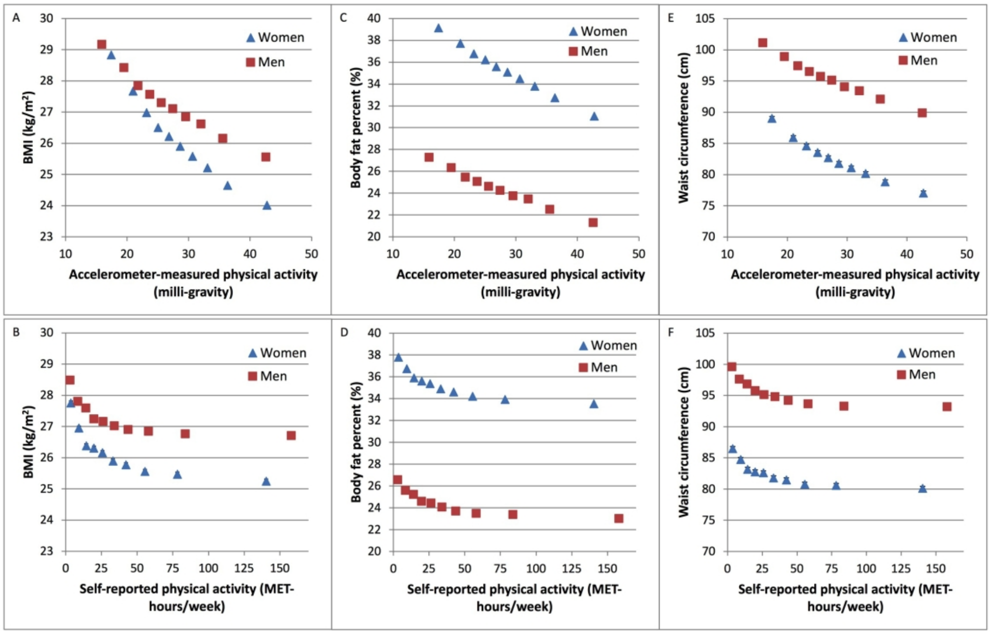

Figure 1. Association of self-reported and accelerometer-measured physical activity with adiposity variables in UK BiobankAssociation of A) accelerometer-measured and B) self-reported physical activity with BMIAssociation of C) accelerometer-measured and D) self-reported physical activity with body fat percentAssociation of E) accelerometer-measured and F) self-reported physical activity with waist circumferencePhysical activity was grouped into tenths, separately in men and women.Adjusted geometric means (from linear regression models) for BMI, body fat percent, and waist circumference are plotted against the median value within each tenth of self-reported or accelerometer-measured physical activity. Adjusted geometric means are represented by squares for men and triangles for women. These analyses are stratified by age at recruitment, region of recruitment, and socioeconomic status, and are adjusted for educational qualifications, employment status, smoking status, and alcohol intake frequency. Analyses in women are additionally adjusted for parity and hormone replacement therapy use. The figure shows point estimates and 95% confidence intervals.Abbreviations: BMI, body mass index; MET, metabolic equivalent

196x126mm (300 x 300 DPI)

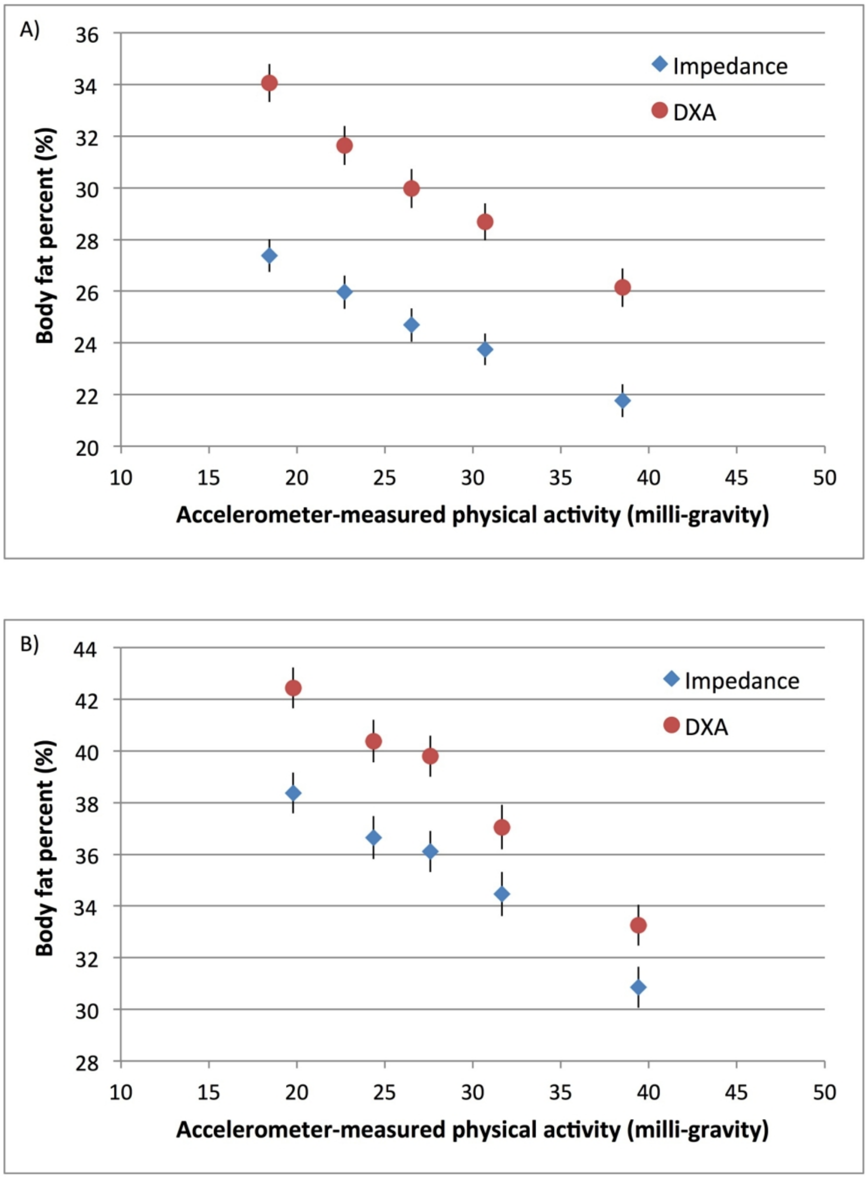

Figure 2. Association of accelerometer-measured physical activity with body fat percent measured by impedance and DXA in UK Biobank A) men (n=1,185) and B) women (n=1,272)Physical activity was grouped into fifths, separately in men and women.Adjusted geometric means (from linear regression models) for body fat percent are plotted against the median value within each fifth of accelerometer-measured physical activity. Adjusted geometric means are represented by diamonds for body fat percent measured by impedance and circles for body fat percent measured by DXA.These analyses are restricted to participants with measures of body fat percent by both impedance and DXA. Analyses are stratified by age at recruitment, region of recruitment, and socioeconomic status, and are adjusted for educational qualifications, employment status, smoking status, and alcohol intake frequency. Analyses in women are additionally adjusted for parity and hormone replacement therapy use. The figure shows point estimates and 95% confidence intervals.Abbreviations: DXA, dual-energy X-ray absorptiometry

150x198mm (300 x 300 DPI)

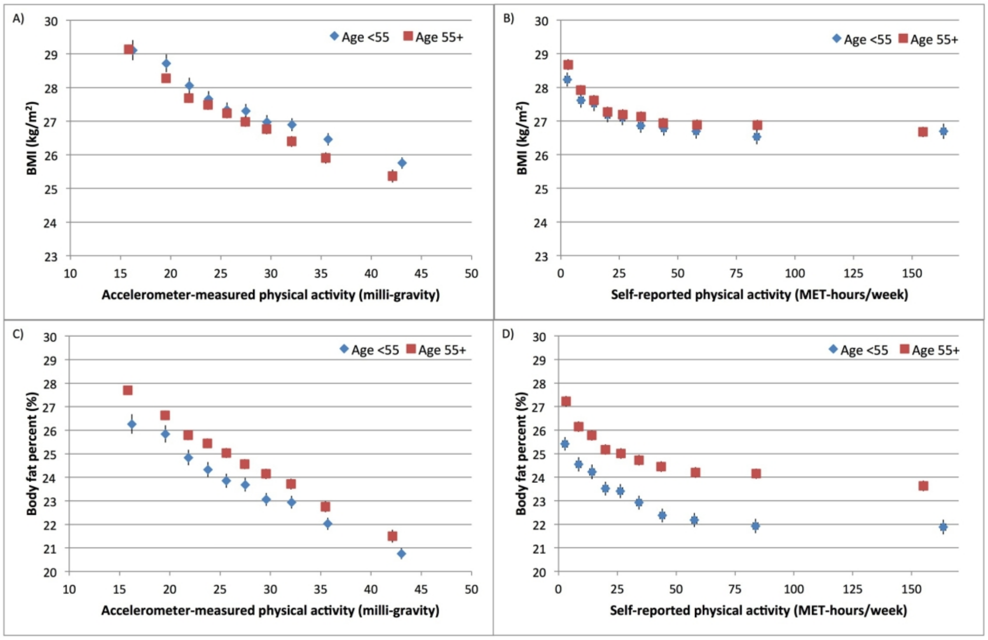

Figure 3. Association of self-reported and accelerometer-measured physical activity with adiposity variables by age group in UK Biobank menAssociation of physical activity measured by A) accelerometer and B) self-reported questionnaire with BMI.Association of physical activity measured by C) accelerometer and D) self-reported questionnaire with body fat percent.Physical activity was grouped into tenths.Adjusted geometric means (from linear regression models) for BMI and body fat percent are plotted against the median value within each tenth of self-reported or accelerometer-measured physical activity. Adjusted geometric means are represented by diamonds for those under age 55 and squares for those ages 55 or older. These analyses are stratified by age at recruitment, region of recruitment, and socioeconomic status, and are adjusted for educational qualifications, employment status, smoking status, and alcohol intake frequency. The figure shows point estimates and 95% confidence intervals.Abbreviations: BMI, body mass index; MET, metabolic equivalent

196x128mm (300 x 300 DPI)

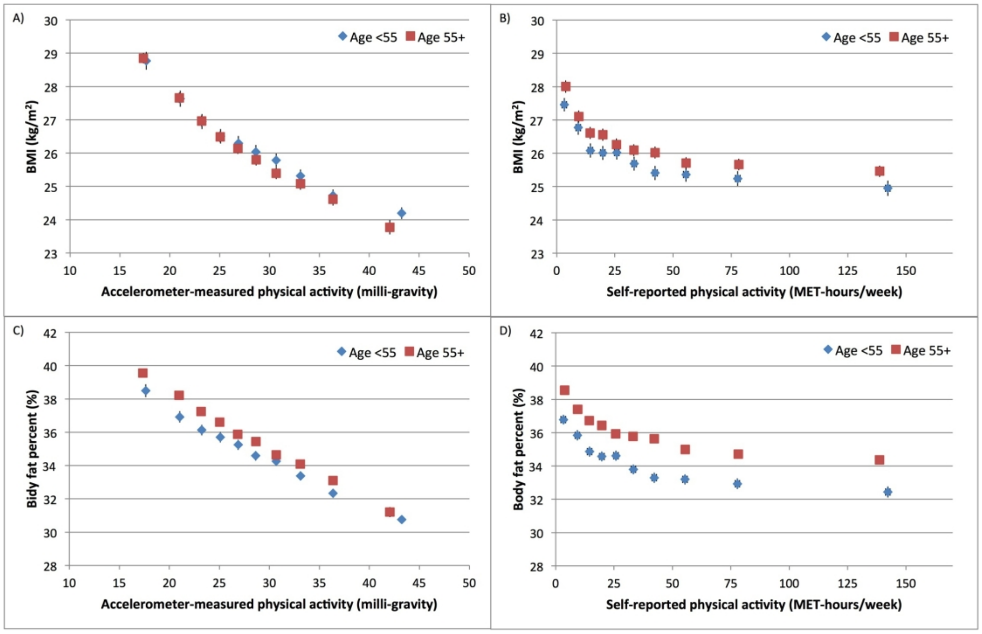

Figure 4. Association of self-reported and accelerometer-measured physical activity with adiposity variables by age group in UK Biobank womenAssociation of physical activity measured by A) accelerometer and B) self-reported questionnaire with BMI.Association of physical activity measured by C) accelerometer and D) self-reported questionnaire with body fat percent.Physical activity was grouped into tenths.Adjusted geometric means (from linear regression models) for BMI and body fat percent are plotted against the median value within each tenth of self-reported or accelerometer-measured physical activity. Adjusted geometric means are represented by diamonds for those under age 55 and squares for those ages 55 or older.These analyses are stratified by age at recruitment, region of recruitment, and socioeconomic status, and are adjusted for educational qualifications, employment status, smoking status, and alcohol intake frequency, parity, and hormone replacement therapy use. The figure shows point estimates and 95% confidence intervals.Abbreviations: BMI, body mass index; MET, metabolic equivalent

195x126mm (300 x 300 DPI)

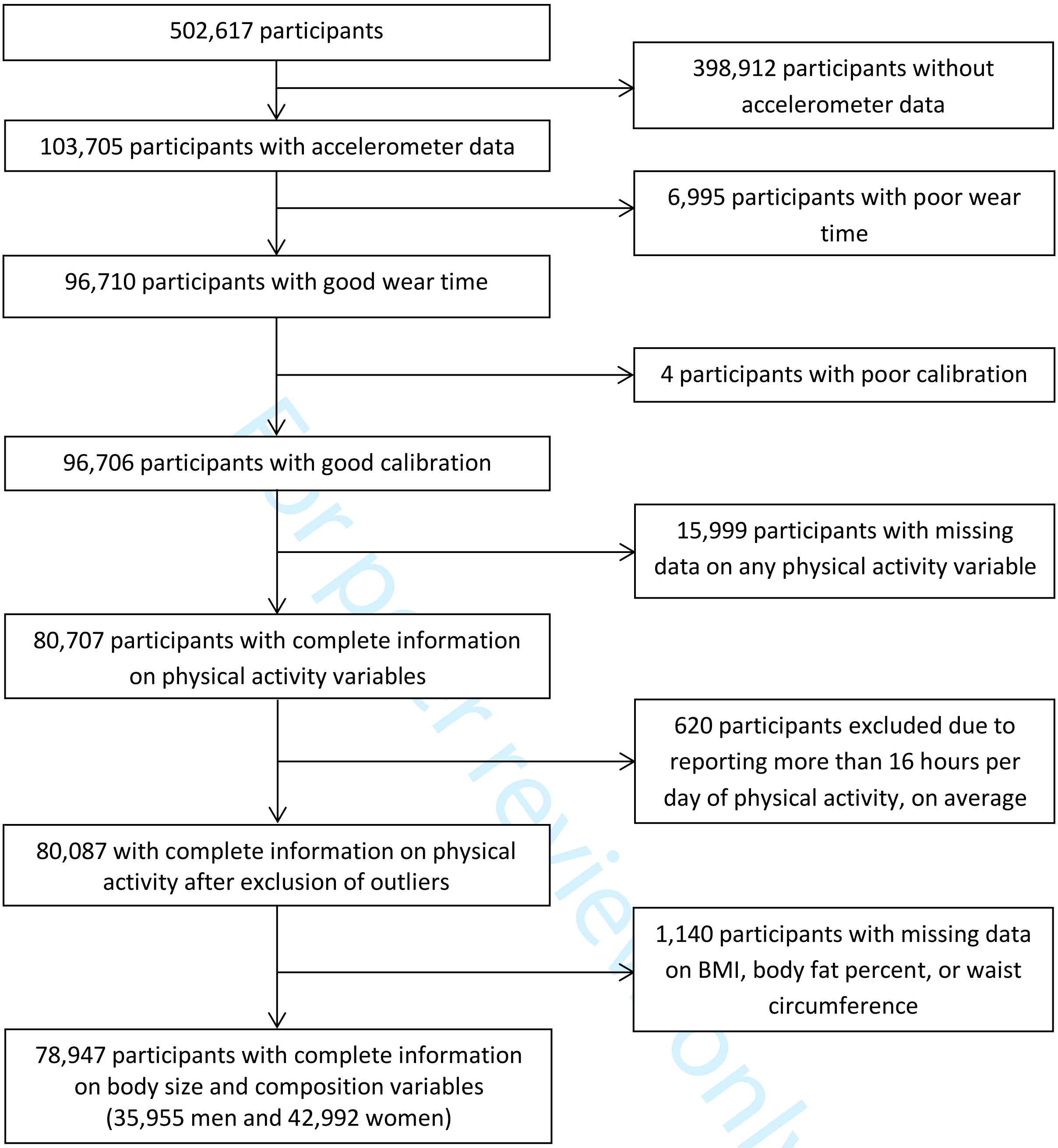

For peer review only

Supplementary Table 1. Physical activity questions from the UK Biobank baseline questionnaire

Question	Potential Responses
In a typical WEEK, on how many days did you walk for at least 10 minutes at a time? (Include walking that you do at work, travelling to and from work, and for sport or leisure)	Number of days, “Do not know”, “Unable to walk”, or “Prefer not to answer”
How many minutes did you usually spend walking on a typical DAY?	Quantity of minutes, “Do not know”, or “Prefer not to answer”
In a typical WEEK, on how many days did you do 10 minutes or more of moderate physical activities like carrying light loads, cycling at normal pace? (Do not include walking)	Number of days, “Do not know”, or “Prefer not to answer”
How many minutes did you usually spend doing moderate activities on a typical DAY?	Quantity of minutes, “Do not know”, or “Prefer not to answer”
In a typical WEEK, how many days did you do 10 minutes or more of vigorous physical activity? (These are activities that make you sweat or breathe hard such as fast cycling, aerobics, heavy lifting)	Number of days, “Do not know”, or “Prefer not to answer”
How many minutes did you usually spend doing vigorous activities on a typical DAY?	Quantity of minutes, “Do not know”, or “Prefer not to answer”

For peer review only

Supplementary Table 2. Spearman correlation between self-reported physical activity and accelerometer-measured physical activity, according to participant characteristics in UK Biobank men

	N Men (%)	Correlation	95% Confidence Interval
**Total**	35,955	0.24	0.23, 0.25
**Age group at recruitment (years)**			
<55 years	13,214 (36.8)	0.31	0.29, 0.32
55+ years	22,741 (63.3)	0.22	0.21, 0.23
**Socioeconomic status, fifths**			
Top fifth	7,584 (21.1)	0.23	0.21, 0.25
Bottom fifth	6,800 (18.9)	0.26	0.23, 0.28
**BMI (kg/m^2)**			
<25	10,590 (29.5)	0.27	0.25, 0.28
25-29.9	17,874 (49.7)	0.21	0.19, 0.22
>30	7,491 (20.8)	0.22	0.80, 0.24
**College or university degree**			
Yes	16,709 (46.5)	0.25	0.24, 0.27
No	19,246 (53.5)	0.24	0.22, 0.25
**Current employment status**			
In paid employment or self-employed	22,942 (63.8)	0.27	0.26, 0.28
Retired	11,361 (31.6)	0.24	0.22, 0.26
Other	1,652 (4.6)	0.30	0.26, 0.34
**Job involves mainly walking/standing**			
Never or rarely	9,825 (42.8)	0.29	0.27, 0.31
Sometimes	7,534 (32.9)	0.24	0.22, 0.26
Usually or Always	5,574 (24.3)	0.19	0.16, 0.21
**Job involves heavy manual work**			
Never, rarely	16,443 (71.7)	0.27	0.26, 0.29
Sometimes	4,160 (18.1)	0.17	0.14, 0.19
Usually or Always	2,335 (10.2)	0.12	0.08, 0.16
**Alcohol intake frequency**			
Weekly or more	29,421 (81.8)	0.23	0.22, 0.24
Less than weekly	6,530 (18.2)	0.28	0.26, 0.30
**Smoking status**			
Never	18,928 (52.6)	0.26	0.24, 0.27
Ever	16,964 (47.2)	0.22	0.21, 0.24
**Long-standing illness or disability**			
No	25,129 (69.9)	0.23	0.22, 0.24
Yes	10,825 (30.1)	0.25	0.23, 0.27

For peer review only

Supplementary Table 3. Spearman correlation between self-reported physical activity and accelerometer-measured physical activity, according to participant characteristics in UK Biobank women

	N Women (%)	Correlation	95% Confidence Interval
**Total**	42,992	0.22	0.21, 0.23
**Age group at recruitment (years)**			
<55 years	18,973 (44.1)	0.26	0.25, 0.28
55+ years	24,019 (55.9)	0.20	0.19, 0.22
**Socioeconomic status, fifths**			
Top fifth	8,401 (19.5)	0.22	0.30, 0.24
Bottom fifth	8,744 (20.3)	0.22	0.30, 0.24
**BMI (kg/m^2)**			
<25	20,255 (47.1)	0.21	0.20, 0.23
25-29.9	15,146 (35.2)	0.18	0.17, 0.20
>30	7,591 (17.7)	0.15	0.13, 0.17
**College or university degree**			
Yes	19,214 (44.7)	0.22	0.21, 0.24
No	23,778 (55.3)	0.22	0.20, 0.23
**Current employment status**			
In paid employment or self-employed	26,693 (62.1)	0.24	0.23, 0.25
Retired	12,710 (29.6)	0.22	0.20, 0.24
Other	3,589 (8.4)	0.30	0.27, 0.33
**Job involves mainly walking/standing**			
Never or rarely	12,191 (45.7)	0.25	0.23, 0.27
Sometimes	7,839 (29.4)	0.21	0.19, 0.23
Usually or Always	6,648 (24.9)	0.18	0.16, 0.20
**Job involves heavy manual work**			
Never, rarely	20,762 (77.8)	0.24	0.22, 0.25
Sometimes	4,353 (16.3)	0.17	0.14, 0.20
Usually or Always	1,567 (5.9)	0.13	0.08, 0.18
**Alcohol intake frequency**			
Weekly or more	29,829 (69.4)	0.22	0.21, 0.23
Less than weekly	13,152 (30.6)	0.21	0.20, 0.23
**Smoking status**			
Never	25,998 (60.5)	0.21	0.20, 0.22
Ever	16,936 (39.4)	0.23	0.22, 0.25
**Long-standing illness or disability**			
No	32,307 (75.2)	0.21	0.20, 0.22
Yes	10,685 (24.9)	0.23	0.21, 0.24

For peer review only

Supplementary Table 4. Association of self-reported and accelerometer-measured physical activity with measures of adiposity

	N	BMI (kg/m^2)	95% CI	Body fat percent	95% CI	Waist circumference (cm)	95% CI
**Men**							
**Questionnaire**							
Bottom tenth (least active)	3,643	0.00 (reference)		0.00 (reference)		0.00 (reference)	
2nd tenth	3,742	-0.69	-0.87, -0.52	-0.99	-1.23, -0.74	-1.97	-2.45, -1.50
3rd tenth	3,410	-0.91	-1.09, -0.73	-1.42	-1.68, -1.17	-2.89	-3.38, -2.41
4th tenth	3,677	-1.22	-1.40, -1.05	-1.97	-2.22, -1.72	-3.81	-4.29, -3.34
5th tenth	3,522	-1.32	-1.50, -1.14	-2.14	-2.39, -1.88	-4.47	-4.95, -3.99
6th tenth	3,584	-1.45	-1.63, -1.27	-2.50	-2.75, -2.24	-4.79	-5.27, -4.31
7th tenth	3,596	-1.59	-1.76, -1.41	-2.88	-3.13, -2.63	-5.41	-5.89, -4.93
8th tenth	3,591	-1.63	-1.81, -1.45	-3.09	-3.34, -2.83	-5.92	-6.40, -5.44
9th tenth	3,595	-1.71	-1.89, 1.53	-3.21	-3.46, -2.95	-6.33	-6.81, -5.85
Top tenth (most active)	3,595	-1.77	-1.95, -1.59	-3.56	-3.81, -3.31	-6.42	-6.90, -5.93
**Accelerometer**							
Bottom tenth (least active)	3,598	0.00 (reference)		0.00 (reference)		0.00 (reference)	
2nd tenth	3,604	-0.77	-0.95, -0.60	-0.99	-1.24, -0.75	-2.23	-2.70, -1.76
3rd tenth	3,592	-1.33	-1.50, -1.15	-1.83	-2.07, -1.58	-3.69	-4.16, -3.22
4th tenth	3,610	-1.60	-1.77, -1.42	-2.20	-2.45, -1.95	-4.59	-5.06, -4.12
5th tenth	3,597	-1.88	-2.06, -1.71	-2.69	-2.94, -2.44	-5.43	-5.90, -4.96
6th tenth	3,585	-2.07	-2.24, -1.89	-3.04	-3.28, -2.79	-6.00	-6.47, -5.53
7th tenth	3,589	-2.32	-2.49, -2.14	-3.52	-3.77, -3.27	-7.01	-7.49, -6.54
8th tenth	3,594	-2.57	-2.75, -2.39	-3.84	-4.09, -3.59	-7.69	-8.17, -7.22
9th tenth	3,591	-3.02	-3.20, -2.84	-4.75	-5.00, -4.50	-9.01	-9.48, -8.53
Top tenth (most active)	3,595	-3.61	-3.79, -3.43	-5.98	-6.24, -5.73	-11.23	-11.72, -10.75

For peer review only

**Women**							
**Questionnaire**							
Bottom tenth (least active)	4,433	0.00 (reference)		0.00 (reference)		0.00 (reference)	
2nd tenth	4,187	-0.81	-1.00, -0.61	-1.05	-1.33, -0.78	-1.77	-2.25, -1.29
3rd tenth	4,278	-1.37	-1.57, -1.18	-1.89	-2.16, -1.61	-3.33	-3.81, -2.86
4th tenth	4,318	-1.44	-1.64, -1.25	-2.16	-2.43, -1.88	-3.74	-4.22, -3.27
5th tenth	4,296	-1.60	-1.79, -1.40	-2.44	-2.72, -2.17	-3.92	-4.39, -3.44
6th tenth	4,308	-1.86	-2.05, -1.66	-2.89	-3.16, -2.61	-4.71	-5.18, -4.23
7th tenth	4,276	-1.98	-2.18, -1.79	-3.18	-3.45, -2.90	-5.02	-5.50, -4.55
8th tenth	4,300	-2.20	-2.39, -2.00	-3.57	-3.85, -3.30	-5.73	-6.20, -5.25
9th tenth	4,305	-2.28	-2.48, -2.09	-3.86	-4.14, -3.59	-5.91	-6.39, -5.44
Top tenth (most active)	4,291	-2.51	-2.70, -2.31	-4.25	-4.52, -3.97	-6.39	-6.87, -5.91
**Accelerometer**							
Bottom tenth (least active)	4,314	0.00 (reference)		0.00 (reference)		0.00 (reference)	
2nd tenth	4,292	-1.14	-1.33, -0.95	-1.43	-1.70, -1.16	-3.02	-3.49, -2.56
3rd tenth	4,293	-1.84	-2.03, -1.65	-2.35	-2.62, -2.09	-4.42	-4.89, -3.95
4th tenth	4,307	-2.31	-2.50, -2.12	-2.90	-3.17-2.63	-5.43	-5.90, -4.97
5th tenth	4,312	-2.60	-2.79, -2.41	-3.52	-3.79, -3.25	-6.27	-6.74, -5.80
6th tenth	4,286	-2.92	-3.11, -2.72	-4.05	-4.32, -3.79	-7.18	-7.65, -6.71
7th tenth	4,292	-3.22	-3.42, -3.04	-4.63	-4.90, -4.36	-7.88	-8.35, -7.41
8th tenth	4,301	-3.60	-3.80, -3.41	-5.34	-5.61, -5.07	-8.80	-9.27, -8.33
9th tenth	4,305	-4.17	-4.36, -3.98	-6.37	-6.64, -6.10	-10.12	-10.59, -9.64
Top tenth (most active)	4,290	-4.80	-4.99, -4.60	-8.06	-8.33, -7.78	-11.92	-12.39, -11.44

Analyses are adjusted for age, socioeconomic status, alcohol intake, smoking status, educational qualifications, and employment status. Analyses are further adjusted for parity and hormone replacement therapy use in women.

For peer review only

Supplementary Table 5. Association of accelerometer-measured physical activity with body fat percent, measured by bioelectrical impedance and dual energy X-ray absorptiometry (DXA) in UK Biobank

Accelerometer-measured physical activity	N	Mean body fat percent	95% CI	Coefficient	95% CI
**Men**		**Measured by impedance**			
Bottom fifth (least active)	237	27.4	26.7, 28.0	0.00 (reference group)	
2nd fifth	231	26.0	25.3, 26.6	-1.29	-2.21, -0.38
3rd fifth	229	24.7	24.0, 25.3	-2.63	-3.55, -1.71
4th fifth	253	23.8	23.1, 24.4	-3.46	-4.36, -2.56
Top fifth (most active)	235	21.8	21.1, 22.4	-5.51	-6.45, -4.58
**Men**		**Measured by DXA**			
Bottom fifth (least active)	237	34.1	33.3, 34.8	0.00 (reference group)	
2nd fifth	231	31.6	30.9, 32.4	-2.38	-3.45, -1.31
3rd fifth	229	30.0	29.2, 30.7	-4.12	-5.20, -3.05
4th fifth	253	28.7	28.0, 29.4	-5.48	-6.54, -4.42
Top fifth (most active)	235	26.1	25.4, 26.9	-8.18	-9.27, -7.08
**Women**		**Measured by impedance**			
Bottom fifth (least active)	270	38.4	37.6, 39.1	0.00 (reference group)	
2nd fifth	244	36.6	35.9, 37.4	-1.46	-2.55, -0.37
3rd fifth	265	36.1	35.4, 36.9	-1.93	-3.01, -0.85
4th fifth	228	34.5	33.7, 35.3	-3.43	-4.55, -2.31
Top fifth (most active)	265	30.8	30.1, 31.6	-6.97	-8.07, -5.87
**Women**		**Measured by DXA**			
Bottom fifth (least active)	270	42.5	41.7, 43.3	0.00 (reference group)	
2nd fifth	244	40.4	39.6, 41.2	-1.87	-3.04, -0.71
3rd fifth	265	39.8	39.0, 40.6	-2.55	-3.70, -1.40
4th fifth	228	37.1	36.2, 37.9	-4.98	-6.17, -3.78
Top fifth (most active)	265	33.3	32.5, 34.1	-8.83	-10.0, -7.65

Analyses are adjusted for age, socioeconomic status, alcohol intake, smoking status, educational qualifications, and employment status. Analyses are further adjusted for parity and hormone replacement therapy use in women.

For peer review only

STROBE Statement—Checklist of items that should be included in reports of ***cross-sectional studies***

	Item No	Recommendation
**Title and abstract**	1	☑ (*a*) Indicate the study's design with a commonly used term in the title or the abstract pages 1-2
		☑ (*b*) Provide in the abstract an informative and balanced summary of what was done and what was found pages 2-3
**Introduction**		
Background/rationale	2	☑ Explain the scientific background and rationale for the investigation being reported page 4
Objectives	3	☑ State specific objectives, including any prespecified hypotheses page 4
**Methods**		
Study design	4	☑ Present key elements of study design early in the paper pages 5-9
Setting	5	☑ Describe the setting, locations, and relevant dates, including periods of recruitment, exposure, follow-up, and data collection pages 5-7
Participants	6	☑ (*a*) Give the eligibility criteria, and the sources and methods of selection of participants pages 5-6
Variables	7	☑ Clearly define all outcomes, exposures, predictors, potential confounders, and effect modifiers. Give diagnostic criteria, if applicable pages 6-8
Data sources/ measurement	8*	☑ For each variable of interest, give sources of data and details of methods of assessment (measurement). Describe comparability of assessment methods if there is more than one group pages 6-7
Bias	9	☑ Describe any efforts to address potential sources of bias page 4
Study size	10	☑ Explain how the study size was arrived at pages 5-6
Quantitative variables	11	☑ Explain how quantitative variables were handled in the analyses. If applicable, describe which groupings were chosen and why pages 6-8
Statistical methods	12	☑ (*a*) Describe all statistical methods, including those used to control for confounding pages 7-9
		☑ (*b*) Describe any methods used to examine subgroups and interactions pages 8-9
		☑ (*c*) Explain how missing data were addressed pages 8-9
		N/A (*d*) If applicable, describe analytical methods taking account of sampling strategy
		☑ (*e*) Describe any sensitivity analyses pages 8-9
**Results**		
Participants	13*	☑ (a) Report numbers of individuals at each stage of study—eg numbers potentially eligible, examined for eligibility, confirmed eligible, included in the study, completing follow-up, and analysed page 9 and Supplementary Figure 1
		☑ (b) Give reasons for non-participation at each stage Supplementary Figure 1
		☑ (c) Consider use of a flow diagram Supplementary Figure 1
Descriptive data	14*	☑ (a) Give characteristics of study participants (eg demographic, clinical, social) and information on exposures and potential confounders pages 9-10
		☑ (b) Indicate number of participants with missing data for each variable of interest pages 5-6, Supplementary Figure 1
Outcome data	15*	☑ Report numbers of outcome events or summary measures pages 10-12, figures
Main results	16	☑ (*a*) Give unadjusted estimates and, if applicable, confounder-adjusted estimates and their precision (eg, 95% confidence interval). Make clear which confounders were adjusted for and why they were included page 8, figures, supplementary tables,

For peer review only

		22-24 (figure legends)
		☑ (*b*) Report category boundaries when continuous variables were categorized page 9
		N/A (*c*) If relevant, consider translating estimates of relative risk into absolute risk for a meaningful time period
Other analyses	17	☑ Report other analyses done—eg analyses of subgroups and interactions, and sensitivity analyses pages 11-12
**Discussion**		
Key results	18	☑ Summarise key results with reference to study objectives pages 12-13
Limitations	19	☑ Discuss limitations of the study, taking into account sources of potential bias or imprecision. Discuss both direction and magnitude of any potential bias pages 15-16
Interpretation	20	☑ Give a cautious overall interpretation of results considering objectives, limitations, multiplicity of analyses, results from similar studies, and other relevant evidence pages 13-16
Generalisability	21	☑ Discuss the generalisability (external validity) of the study results page 16
**Other information**		
Funding	22	☑ Give the source of funding and the role of the funders for the present study and, if applicable, for the original study on which the present article is based page 16

*Give information separately for exposed and unexposed groups.

Note: An Explanation and Elaboration article discusses each checklist item and gives methodological background and published examples of transparent reporting. The STROBE checklist is best used in conjunction with this article (freely available on the Web sites of PLoS Medicine at http://www.plosmedicine.org/, Annals of Internal Medicine at http://www.annals.org/, and Epidemiology at http://www.epidem.com/). Information on the STROBE Initiative is available at www.strobe-statement.org.

For peer review only

For peer review only - http://bmjopen.bmj.com/site/about/guidelines.xhtml

BMJ Open

Accelerometer compared with questionnaire measures of physical activity in relation to body size and composition: a large cross-sectional analysis of UK Biobank

Journal:	*BMJ Open*
Manuscript ID	bmjopen-2018-024206.R2
Article Type:	Research
Date Submitted by the Author:	23-Nov-2018
Complete List of Authors:	Guo, Wenji; University of Oxford, Nuffield Department of Population Health<br>Key, Timothy; University of Oxford, Nuffield Department of Population Health<br>Reeves, Gillian; University of Oxford, Nuffield Department of Population Health
**Primary Subject Heading**:	Epidemiology
Secondary Subject Heading:	Public health
Keywords:	EPIDEMIOLOGY, adiposity, physical activity, accelerometer, activity monitor

SCHOLARONE™
Manuscripts

Accelerometer compared with questionnaire measures of physical activity in relation to body size and composition: a large cross-sectional analysis of UK Biobank

Wenji Guo, Timothy J Key, Gillian K Reeves

Cancer Epidemiology Unit, Nuffield Department of Population Health, University of Oxford, Oxford, UK (WG, TJK, GKR)

Corresponding author:

Wenji Guo

Cancer Epidemiology Unit

Nuffield Department of Population Health

University of Oxford

Richard Doll Building

Roosevelt Drive

Oxford OX3 7LF, UK

Tel +44 1865 289635

wenji.guo@ndph.ox.ac.uk

wguo8@jhmi.edu

For peer review only

Abbreviations: body mass index (BMI), confidence interval (CI), dual-energy X-ray absorptiometry (DXA), International Physical Activity Questionnaire (IPAQ), metabolic equivalents (METs), standard deviation (SD)

For peer review only - http://bmjopen.bmj.com/site/about/guidelines.xhtml

Abstract

Objectives: Previous studies of the association between physical activity and adiposity are largely based on physical activity and body mass index (BMI) from questionnaires, which are prone to inaccurate and biased reporting. We assessed the associations of accelerometer-measured and questionnaire-measured physical activity with BMI, waist circumference and body fat percent measured by bioelectrical impedance and dual-energy X-ray absorptiometry (DXA).

Design: Cross-sectional analysis of UK Biobank participants

Setting: UK Biobank assessment centers

Participants: 78,947 UK Biobank participants (35,955 men and 42,992 women) aged 40-70 at recruitment, who had physical activity measured by both questionnaire and accelerometer.

Main outcome measures: BMI, waist circumference and body fat percent measured by bioelectrical impedance

Results: Greater physical activity was associated with lower adiposity. Women in the top tenth of accelerometer-measured physical activity had a 4.8 (95% CI: 4.6, 5.0) kg/m^2 lower BMI, 8.1% (95% CI: 7.8, 8.3) lower body fat percent, and 11.9 (95% CI 11.4, 12.4) cm lower waist circumference. Women in the top tenth of questionnaire-measured physical activity had a 2.5 (95% CI: 2.3, 2.7) kg/m^2 lower BMI, 4.3% (95% CI: 4.0, 4.5) lower body fat percent, and 6.4 (95% CI: 5.9, 6.9) cm lower waist circumference, compared to women in the bottom tenths. The patterns were similar in men and also similar with body fat percent measured by DXA compared to impedance.

For peer review only

For peer review only - http://bmjopen.bmj.com/site/about/guidelines.xhtml

Conclusion: Our findings of approximately twofold stronger associations between physical activity and adiposity with objectively-measured than with self-reported physical activity emphasize the need to incorporate objective measures in future studies.

Strengths and limitations of this study:

- This study utilizes data on physical activity objectively measured by accelerometer rather than only self-reported data from questionnaires, which may be prone to inaccurate and potentially biased reporting.
- This study is by far the largest study to examine associations of objectively measured physical activity and self-reported physical activity with various measures of adiposity, including body fat percent assessed by bioelectrical impedance and dual energy x-ray absorptiometry.
- Due to the cross-sectional nature of this study, we cannot assess to what extent physical activity is causally related to adiposity.

For peer review only

INTRODUCTION

The prevalence of overweight and obesity is high worldwide and is associated with increased risk of various conditions including heart disease, stroke, hypertension, diabetes, and some cancers (1, 2). Although physical activity is generally accepted to be important for prevention of weight gain, achievement of modest weight loss, and prevention of weight regain after weight loss (3), randomized controlled trials have shown inconsistent results, perhaps partly due to limited duration of interventions and difficulty in long-term adherence to exercise regimens (4), and previous large-scale observational studies are mostly based on self-reported physical activity from questionnaires, which are prone to both inaccurate reporting and reporting bias (5).

Prior studies have demonstrated low to moderate correlation between self-reported and objective accelerometer measures of physical activity (6, 7). Self-reported and accelerometer-measured physical activity capture different aspects of physical activity with limitations unique to each (7). However, research methods utilizing more objective measures of physical activity, along with more detailed measures of body fat, are needed to reduce measurement error and more accurately characterize the association between physical activity and adiposity.

We examined the association between physical activity and adiposity, with accelerometer-measured compared to self-reported physical activity in nearly 80,000 participants. These associations were assessed using various measures of adiposity, including BMI, waist circumference, and body fat percent measured by both bioelectrical impedance and dual-energy X-ray absorptiometry (DXA). We also explored how the associations vary by age.

For peer review only - http://bmjopen.bmj.com/site/about/guidelines.xhtml

METHODS

Data source

Data were obtained from UK Biobank. Details of UK Biobank design, rationale, and survey methods have been described elsewhere (8). Information on data available and access procedures are described on the study website (http://www.ukbiobank.ac.uk/). UK Biobank has approval from the National Information Governance Board for Health & Social Care in England and Wales, the North West Multi-centre Research Ethics Committee, and the Community Health Index Advisory Group in Scotland. Written informed consent was provided by all participants.

Study participants

The complete UK Biobank dataset includes 502,617 UK adults (229,164 men and 273,453 women) between 40 to 70 years of age at recruitment during 2006 to 2010. During the baseline assessment center visit, participants completed a touchscreen questionnaire which included questions on socio-demographics, lifestyle, health and medical history, and sex-specific factors. The present study was restricted to participants with available accelerometer data (n=103,705). Participants were excluded if they did not have at least 72 hours of data and also data in each one-hour period of the 24-hour cycle across multiple days (n=6,995). Participants were also excluded if they had insufficient data for calibration (n=4). Participants who had missing data on any of the physical activity variables used in our analyses were excluded (n=15,999). Participants who reported physical activity greater than an average of 16 hours per day (n=620) were also excluded as recommended by the International Physical Activity Questionnaire (IPAQ) scoring guidelines, which can be accessed at file:///H:/Downloads/GuidelinesforDataProcessingandAnalysisoftheInternationalPhysicalActivityQuestionnaireIPAQShortandLongForms.pdf. Finally, participants with missing data on

For peer review only - http://bmjopen.bmj.com/site/about/guidelines.xhtml

BMI (n=146), body fat percent (n=988), and waist circumference (n=6) were excluded. The analyses included 35,955 men and 42,992 women (**Supplementary Figure 1**).

Self-reported physical activity

Physical activity questions from the baseline questionnaire captured the frequency and duration of three intensities of activity (walking, moderate, and vigorous). Participants were asked how many days per week they typically engaged in each category of activity. For each category in which an answer of one or more days was given, the participant was subsequently asked the number of minutes on average spent on the activity per day. Questions were adapted from the IPAQ, a validated survey instrument (9), and are listed in **Supplementary Table 1**. Metabolic equivalents (METs) were used to quantify physical activity; 1 MET is expended by sitting quietly for 1 hour, and the MET value reflects the ratio of energy expended per kilogram of body weight per hour to that expended when sitting quietly (10). The number of minutes per day engaged in each level of activity was multiplied by the respective MET score for the corresponding level of activity (3.3 for walking, 4.0 for moderate physical activity, and 8.0 for vigorous physical activity) (11). MET minutes per day were converted to MET hours per week. The total amount of METs was calculated by summing total METs from the walking, moderate, and vigorous activity levels. Following IPAQ scoring guidelines, physical activity of less than 10 minutes per day for any category was recoded to 0.

Accelerometer-measured physical activity

A total of 236,519 participants, all of whom had provided a valid email address, were invited to participate in a seven day accelerometer study between February 2013 and December 2015 (on average, approximately 5.5 years after recruitment when baseline physical activity was self-reported). Starting in June 2013, participants were sent wrist-worn

For peer review only - http://bmjopen.bmj.com/site/about/guidelines.xhtml

triaxial accelerometers (Axivity AX3, Newcastle upon Tyne, UK) that were programmed to capture three-dimensional acceleration data at 100 Hz with a dynamic range of ±8 g. Participants were also given instructions to wear the accelerometer on their dominant wrist continuously for seven days and then to send the device to the coordinating center using the provided prepaid envelope. Further details on data collection, processing, and analysis can be found elsewhere (12). We used the “overall acceleration average” variable (data field 90012) in the present analyses.

For peer review only

Anthropometry and body composition

At the UK Biobank baseline interview, trained staff measured standing height using the Seca 202 device (Seca, Hamburg, Germany). BMI was calculated by dividing weight (kg) by the square of standing height (m^2). The Wessex non-stretchable sprung tape measure (Wessex, United Kingdom) was used to measure waist circumference at the level of the umbilicus. The Tanita BC-418MA body composition analyzer (Tanita, Tokyo, Japan) was used to measure body fat percent using bioelectrical impedance. DXA was used to measure fat percent on a subset of 2,457 participants included in the present study, beginning in 2014 using the GE-Lunar iDXA (GE Healthcare, Chicago, USA).

Statistical analyses

Baseline characteristics were summarized by physical activity (least active fifth, most active fifth, and overall) separately for men and women. Since self-reported physical activity was not normally distributed, Spearman’s correlation coefficients were used to measure the strength of correlations between self-reported and accelerometer-measured physical activity in the overall population and in subgroups based on sociodemographic characteristics.

For peer review only - http://bmjopen.bmj.com/site/about/guidelines.xhtml

Self-reported and accelerometer-measured physical activity were categorized into tenths and the median value within each category of physical activity is shown in the figures. The associations of physical activity and adiposity measures were examined using multivariable linear regression, separately in men and women. Analyses comparing the association of accelerometer-measured physical activity with body fat percent, measured by bioelectrical impedance and DXA were restricted to participants with both measures. Likelihood ratio tests were used to assess whether the associations between physical activity and adiposity were modified by age (<55 years or 55+ years), separately for self-reported and accelerometer-measured physical activity.

Covariates were determined a priori and were 5-year age at recruitment categories, socioeconomic status as indicated by fifths of Townsend deprivation index (13), educational qualifications, employment status, smoking status (never, previous, current), and alcohol intake frequency. Analyses in women were additionally adjusted for parity (nulliparous, 1, 2, 3, 4 or more births) and hormone replacement therapy use (never, previous, current). As a covariate, educational qualification was grouped into the following categories: vocational qualifications, national exams at age 16 (O levels, GCSEs, CSEs, or equivalent), optional national exams at ages 17-18 years (A levels, AS levels, or equivalent), and college or university degree. Employment status was categorized as paid or self-employed, retired, looking after home and/or family, unemployed, doing unpaid or voluntary work, unable to work due to sickness or disability, and student. Alcohol intake was categorized as never, special occasions only, 1-3 times a month, 1-2 times a week, 3-4 times a week, and daily or almost daily.

Missing data were grouped in a separate unknown category for each covariate. There were less than 1% missing data for all covariates except for educational qualifications (7.4% missing data). To assess the impact of missing values, a sensitivity analysis restricted to

For peer review only - http://bmjopen.bmj.com/site/about/guidelines.xhtml

participants with known values for all covariates was conducted. We also conducted sensitivity analyses to assess the impact of excluding participants who reported long-term illness, disability or infirmity, participants who reported fair or poor health rather than excellent or good health, and participants whose jobs usually or always required heavy manual work. Analyses were conducted using STATA, version 15.0 (Stata Corp LP, College Station, TX).

Patient and public involvement

This study did not involve patients and the public.

RESULTS

Characteristics of the study population by least active and most active fifth of accelerometer-measured physical activity are shown in **Table 1**.

Table 1. Characteristics of the UK Biobank study population, according to fifths of accelerometer-measured physical activity.

	Least active men	Most active men	All men		Least active women	Most active women	All women
	<20.8 milli-gravity	≥33.7 milli-gravity			<22.2 milli-gravity	≥34.6 milli-gravity	
**Number of participants**	7,202	7,186	35,955		8,606	8,595	42,992
**Age at recruitment (years), mean (SD)**	59.7 (7.0)	53.4 (7.7)	56.7 (7.9)		58.0 (7.4)	52.6 (7.4)	55.3 (7.7)
**Lowest fifth of socioeconomic status**	1,520 (21.1%)	1,351 (18.8%)	6,800 (18.9%)		1,897 (22.0%)	1,699 (19.8%)	8,744 (20.3%)
**Accelerometer-measured physical activity (milli-gravity), mean (SD)**	17.5 (2.6)	40.5 (7.8)	27.6 (8.7)		18.9 (2.7)	40.6 (6.3)	28.7 (8.0)
**Self-reported physical activity (MET-hours/week), median (IQR)**	20.7 (9.0, 42.6)	44.2 (23.7, 80.9)	29.9 (14.2, 58.1)		21.3 (9.9, 41.7)	40.2 (21.8, 73.2)	29.3 (14.4, 55.3)
**Height (cm), mean (SD)**	176.3 (6.8)	176.4 (6.6)	176.5 (6.6)		163.2 (6.3)	163.7 (6.1)	163.5 (6.2)
**Weight (kg), mean (SD)**	89.4 (15.4)	80.8 (11.4)	84.9 (13.5)		75.5 (15.6)	65.0 (10.3)	69.9 (13.2)
**BMI (kg/m²), mean (SD)**	28.8 (4.6)	25.9 (3.3)	27.2 (4.0)		28.3 (5.7)	24.3 (3.7)	26.2 (4.8)
**Body fat percent (%) [a], mean (SD)**	27.0 (5.6)	21.7 (5.4)	24.4 (5.7)		38.7 (6.6)	31.7 (6.4)	35.3 (6.8)
**Waist circumference (cm), mean (SD)**	100.1 (11.7)	90.9 (9.3)	95.4 (10.8)		87.9 (13.3)	77.6 (9.5)	82.4 (11.7)

For peer review only - http://bmjopen.bmj.com/site/about/guidelines.xhtml

**College or university degree**	3,018 (41.9%)	3,365 (46.8%)	16,709 (46.5%)		3,586 (41.7%)	4,060 (47.2%)	19,214 (44.7%)
**Current employment status**							
Paid employment/self-employed	3,608 (50.1%)	5,420 (75.4%)	22,942 (63.8%)		4,401 (51.1%)	6,101 (71.0%)	26,693 (62.1%)
Retired	3,107 (43.1%)	1,451 (20.2%)	11,361 (31.6%)		3,517 (40.9%)	1,591 (18.5%)	12,710 (29.6%)
Other	487 (6.8%)	315 (4.4%)	1,652 (4.6%)		688 (8.0%)	903 (10.5%)	3,589 (8.3%)
**Job involves mainly walking/standing** [b]	707 (19.6%)	1,742 (32.1%)	5,574 (24.3%)		893 (20.3%)	1,926 (31.6%)	6,648 (24.9%)
**Job involves heavy manual work** [c]	272 (7.5%)	912 (16.8%)	2,335 (10.2%)		170 (3.9%)	576 (9.4%)	1,567 (5.9%)
**Weekly or more frequent alcohol intake**	5,545 (77.0%)	5,989 (83.3%)	29,421 (81.8%)		5,295 (61.5%)	6,292 (73.2%)	29,829 (69.4%)
**Ever smoker**	3,801 (52.8%)	3,126 (43.5%)	16,964 (47.2%)		3,583 (41.6%)	3,212 (37.4%)	16,936 (39.4%)
**Long-standing illness or disability**	3,089 (42.9%)	1,543 (21.5%)	10,825 (30.1%)		3,145 (36.5%)	1,449 (16.9%)	1,0685 (24.9%)

[a] Body fat percent was measured by bioelectrical impedance

[b] Participants who reported their work "usually" or "always" involved walking or standing for most of the time

[c] Participants who reported their work "usually" or "always" involved heavy manual or physical work for most of the time

For peer review only

Mean accelerometer-measured physical activity was 27.6 (standard deviation [SD] 8.7) milli-gravity in men and 28.7 (SD 8.0) milli-gravity in women. The most active participants were on average younger and had lower values for all body size and composition variables. They were more likely to have a college or university degree, be employed rather than retired, have an active job, and consume alcohol at least weekly. The least active participants were more likely to be ever smokers and were also more likely to have a long-standing illness or disability. The correlation between questionnaire and accelerometer-measured physical activity, recorded on average 5.5 years later, was 0.24 (95% confidence interval [CI]: 0.23, 0.25) in men (**Supplementary Table 2**) and 0.22 (95% CI: 0.21, 0.23) in women (**Supplementary Table 3**). The correlations were comparatively higher in participants who were younger and in participants who had lower BMI. The correlations were lower among

For peer review only - http://bmjopen.bmj.com/site/about/guidelines.xhtml

participants who reported that their job usually or always involved heavy manual work and/or mainly walking or standing.

The inverse associations between physical activity and all measures of adiposity were linear and approximately twofold larger in models that used accelerometer-measured rather than self-reported physical activity. Since there was heterogeneity in the associations between both self-reported and accelerometer-measured physical activity and adiposity by sex ($P<0.001$), separate analyses were performed in men and women. The mean differences in BMI and body fat percent were greater in women compared to men. Comparing the top to bottom tenth of accelerometer-measured physical activity, the difference in BMI was 4.8 (95% CI: 4.6, 5.0) kg/m^2 in women and 3.6 (95% CI: 3.4, 3.8) kg/m^2 in men (**Figure 1, Supplementary Table 4**).

Women in the top tenth of accelerometer-measured physical activity had an 8.1% (95% CI: 7.8, 8.3) lower body fat percent while women in the top tenth of self-reported physical activity had a 4.3% (95% CI: 4.0, 4.5) lower body fat percent, compared to those in the bottom tenth of physical activity. Men in the top tenth of accelerometer-measured physical activity had a 6.0% (95% CI: 5.7, 6.2) lower body fat percent while men in the top tenth of self-reported physical activity had a 3.6% (95% CI: 3.3, 3.8) lower body fat percent, compared to those in the bottom tenth (**Figure 1, Supplementary Table 4**).

Associations between physical activity and waist circumference were of similar magnitude in men and women, with an approximately twofold larger inverse association between waist circumference and physical activity when measured by accelerometer rather than questionnaire (**Figure 1, Supplementary Table 4**).

The results of sensitivity analyses excluding participants who had any missing values, reported a long-term illness or disability, reported a health rating worse than "good", or

For peer review only - http://bmjopen.bmj.com/site/about/guidelines.xhtml

whose jobs usually or always required heavy manual work did not materially differ from the main findings.

Figure 2 and Supplementary Table 5 show the associations between accelerometer-measured physical activity and bioelectrical impedance-measured body fat percent at baseline (2006-2010) compared to body fat percent measured by DXA starting in May 2014. Body fat percent by impedance at baseline was lower than body fat percent by DXA, measured on average seven years later. For both measures, there was a linear dose-response association between physical activity and body fat percent in both men and women. The inverse associations were stronger when body fat percent was measured by DXA. Compared to the least active women, the most active women had an 8.8% (95% CI: 7.7, 10.0) lower DXA-measured body fat percent and a 7.0% (95% CI: 5.9, 8.1) lower impedance-measured body fat percent (**Figure 2 and Supplementary Table 5**).

Associations between physical activity and measures of adiposity by age group are shown in **Figure 3** for men and **Figure 4** for women. For a given level of accelerometer-measured physical activity, the older participants (over age 55) had a slightly lower BMI but a higher body fat percent compared to their younger counterparts. For women, there was heterogeneity by age in the association between self-reported physical activity and body fat percent (P=0.03) but there was no heterogeneity by age when physical activity was measured by accelerometer (P=0.27).

DISCUSSION

In this large cross-sectional study of nearly 80,000 participants, we found that associations between physical activity and BMI, body fat percent, and waist circumference were stronger when physical activity was measured by accelerometer compared to questionnaire self-reports. Body fat percent measured by DXA at follow-up showed a slightly stronger

For peer review only - http://bmjopen.bmj.com/site/about/guidelines.xhtml

association with physical activity compared to body fat percent measured by bioelectrical impedance at baseline, but the overall pattern of association was similar. The correlation between accelerometer-measured and self-reported physical activity, recorded 5.5 years apart, was lower in participants with higher BMI and in older participants.

There was a consistent dose-response relationship between physical activity and adiposity across the different measures of adiposity, which are highly correlated (14). Our analyses based on accelerometer-measured physical activity suggest an approximately linear inverse association between physical activity and adiposity, with the most active participants having the lowest BMI, body fat percent, and waist circumference. In contrast, the analyses in the same participants based on self-reported physical activity suggest a comparatively small further benefit of physical activity greater than 50 MET-hours a week on adiposity.

We have previously suggested that the steeper inverse association between physical activity and adiposity within the lower range of physical activity could be due to either a comparatively larger benefit of physical activity for those who are relatively inactive or measurement error from over-reporting of high physical activity (14). The present analyses demonstrating an approximately linear dose-response relationship between accelerometer-measured physical activity and adiposity supports the latter explanation and further suggests that over-reporting of total physical activity contributed to the low overall correlation between self-reported and accelerometer-measured physical activity, although the time lag between these two measurements of physical activity may have also contributed to a low overall correlation coefficient. Wrist accelerometer-measured physical activity also has limitations, such as measuring movement of only one part of the body and the inability to reliably capture activities such as cycling (7), it has the major advantage of eliminating both inaccurate reporting that leads to random error as well as reporting bias that may vary by sociodemographic characteristics.

For peer review only - http://bmjopen.bmj.com/site/about/guidelines.xhtml

Measurement error in the self-reported data results in misclassification of individuals by physical activity status. We used the IPAQ short form data processing rules since the UK Biobank questionnaire did not comprehensively cover domain-specific activities, but it is still likely that lower intensity activities were underreported and reported less accurately (15). In contrast, the accelerometers were worn for 24 hours a day, over 7 days. Therefore, the lower correlation between self-reported and accelerometer-measured physical activity in older participants (16) and the heterogeneity by age seen only with the self-reported data may be explained by the observation that, in older adults, a greater proportion of physical activity is of lower intensity (17).

Individuals with higher body fat percent may report moderate and strenuous physical activity less accurately than leaner individuals, based on comparisons between self-reported physical activity and energy expenditure estimated from whole-room indirect calorimeter (18). In agreement with some previous studies, we found that the correlation between physical activity measured by questionnaire and accelerometer-measured physical activity was greater for those with lower BMI (7). This suggests that measurement error of self-reported physical activity may be greater in overweight and obese BMI groups.

We, like several prior studies, found stronger associations between accelerometer-measured physical activity and all measures of adiposity in women compared to men (19–21). This may partly be due to the fact that, in the present study, men were on average objectively less physically active than women. Differences in fat metabolism may also play a role, with a greater proportion of energy derived from lipolysis during exercise in women compared to men (21, 22).

To our knowledge, the present study is the largest to date comparing accelerometer-measured and self-reported physical activity in relation to direct measures of body fat,

For peer review only - http://bmjopen.bmj.com/site/about/guidelines.xhtml

although our results are consistent with prior, smaller studies that suggest a stronger association between adiposity and accelerometer-measured compared to self-reported physical activity (18, 20, 23–26). This study was population-based and recruited from 22 regions throughout the UK (27). A major strength of this study is the availability of both accelerometer-measured physical activity and body fat by impedance in nearly 80,000 participants, together with data on body fat assessed by DXA in over 2,400 participants. Additionally, the accelerometers used in this study were waterproof (12), overcoming a limitation of prior studies where the devices had to be removed for water-based activities (21).

While self-reported physical activity was available at baseline in these data, accelerometer-measured physical activity was assessed only 3-5 years after end of recruitment, which raises the question of whether higher adiposity at baseline predicts lower physical activity levels (28) rather than physical activity determining adiposity. However, our analysis of accelerometer-measured physical activity in relation to DXA-measured body fat percent, which was assessed within the same time frame as accelerometer-measured physical activity, showed similar results to the main analysis based on body fat percent assessed by impedance at baseline. The accelerometer-measured physical activity variable available in UK Biobank at the time of these analyses cannot be directly compared to MET hours of self-reported physical activity. However, Willetts et al. have recently developed physical activity phenotypes using a machine learning model with reference behaviors provided by data from a subset of participants who wore a camera along with the accelerometer (29). Once these variables are made publicly available in UK Biobank, research using these metrics will facilitate the translation of study results into public health messages.

Other limitations include the lack of data on total energy intake for the whole cohort. Although accelerometer-determined physical activity is positively associated with percent of

For peer review only - http://bmjopen.bmj.com/site/about/guidelines.xhtml

lean muscle mass (30), we did not consider this as a confounder in these analyses since we utilized data on direct measures of body fat percent. Since accelerometer-measured time spent in sedentary activity was not available, we did not conduct analyses on sedentary activity. Due to the cross-sectional nature of this study, we cannot assess to what extent physical activity is causally related to adiposity. Highly active individuals may also be more likely to maintain appropriate target dietary energy intake, for example. Although the UK Biobank cohort is not representative of the general population in the UK, results of associations between exposures and health outcomes may be generalizable and would not necessarily require the study population to be representative if the biological basis of the exposure-disease relationship is shared.

In conclusion, our findings based on objective accelerometer data indicate a stronger relationship between physical activity and adiposity than previously thought. Comparison of estimates of physical activity measured by questionnaire and by accelerometer suggest measurement error in self-reported physical activity, emphasizing the need to incorporate objective measures of physical activity in future studies.

For peer review only

Funding: This work was supported by Cancer Research UK, grant number C570/A16491. WG is supported by the Clarendon Fund.

Disclosures: The authors have declared no conflicts of interest.

Contributors: WG, TJK, and GKR were responsible for study concept, design of the study, interpretation of the data, and manuscript writing. WG had primary responsibility for statistical analysis and final content. All authors reviewed and approved the final manuscript.

Dating sharing statement: No additional data are available.

For peer review only - http://bmjopen.bmj.com/site/about/guidelines.xhtml

REFERENCES

1. Must A, Spadano J, Coakley EH, et al. The disease burden associated with overweight and obesity. *JAMA*. 1999;282:1523–9.

2. Afshin A, Forouzanfar MH, Reitsma MB, et al. Health effects of overweight and obesity in 195 countries over 25 years. *N Engl J Med*. 2017;377:13–27.

3. Donnelly JE, Blair SN, Jakicic JM, et al. American College of Sports Medicine position stand. Appropriate physical activity intervention strategies for weight loss and prevention of weight regain for adults. *Med Sci Sport Exerc*. 2009;41:459–71.

4. Catenacci VA, Wyatt HR. The role of physical activity in producing and maintaining weight loss. *Nat Clin Pr End Met*. 2007;3:518–29.

5. Ainsworth BE, Caspersen CJ, Matthews CE, et al. Recommendations to improve the accuracy of estimates of physical activity derived from self report. *J Phys Act Heal*. 2012;9 Suppl 1:S76-84.

6. Koolhaas CM, van Rooij FJ, Cepeda M, et al. Physical activity derived from questionnaires and wrist-worn accelerometers: comparability and the role of demographic, lifestyle, and health factors among a population-based sample of older adults. *Clin Epidemiol*. 2018;10:1–16.

7. Skender S, Ose J, Chang-Claude J, *et al.* Accelerometry and physical activity questionnaires - a systematic review. *BMC Public Health*. 2016;16:515.

8. UK Biobank: Protocol for a large-scale prospective epidemiological resource. 2010. Available from: http://www.ukbiobank.ac.uk/wp-content/uploads/2011/11/UK-Biobank-Protocol.pdf

9. Craig CL, Marshall AL, Sjostrom M, et al. International physical activity questionnaire: 12-country reliability and validity. *Med Sci Sport Exerc*. 2003;35:1381–95.

For peer review only - http://bmjopen.bmj.com/site/about/guidelines.xhtml

10. Ainsworth BE, Haskell WL, Herrmann SD, et al. 2011 Compendium of physical activities: a second update of codes and MET values. *Med Sci Sport Exerc.* 2011;43:1575–81.
11. IPAQ Research Committee. Guidelines for data processing and analysis of the International Physical Activity Questionnaire (IPAQ) - short and long forms. 2005 [cited 2018 May 10]. Available from: https://sites.google.com/site/theipaq/scoring-protocol
12. Doherty A, Jackson D, Hammerla N, Plotz T, Olivier P, Granat MH, et al. Large scale population assessment of physical activity using wrist worn accelerometers: the UK Biobank Study. *PLoS One*. 2017;12:e0169649.
13. Morris R, Carstairs V. Which deprivation? A comparison of selected deprivation indexes. *J Public Heal Med*. 1991;13:318–26.
14. Guo W, Bradbury KE, Reeves GK, Key TJ. Physical activity in relation to body size and composition in women in UK Biobank. *Ann Epidemiol*. 2015;25:406–413 e6.
15. Sallis JF, Saelens BE. Assessment of physical activity by self-report: status, limitations, and future directions. *Res Q Exerc Sport*. 2000;71.
16. Dyrstad SM, Hansen BH, Holme IM, Anderssen SA. Comparison of self-reported versus accelerometer-measured physical activity. *Med Sci Sports Exerc*. 2014 Jan;46(1):99–106.
17. Westerterp KR. Physical activity as determinant of daily energy expenditure. *Physiol Behav*. 2008;93:1039–43.
18. Buchowski MS, Townsend KM, Chen KY, et al. Energy expenditure determined by self-reported physical activity is related to body fatness. *Obes Res*. 1999;7.
19. Sabia S, Cogranne P, van Hees VT, et al. Physical activity and adiposity markers at older ages: accelerometer vs questionnaire data. *J Am Med Dir Assoc*. 2015;16:438.e7-

For peer review only

13.

20. Bann D, Kuh D, Wills AK, et al. Physical activity across adulthood in relation to fat and lean body mass in early old age: findings from the Medical Research Council National Survey of Health and Development, 1946-2010. *Am J Epidemiol*. 2014;179:1197–207.

21. Wanner M, Richard A, Martin B, et al. Associations between self-reported and objectively measured physical activity, sedentary behavior and overweight/obesity in NHANES 2003-2006. *Int J Obes*. 2017;41:186–93.

22. Blaak E. Gender differences in fat metabolism. *Curr Opin Clin Nutr Metab Care*. 2001;4:499–502.

23. Hedrington MS, Davis SN. Sexual dimorphism in glucose and lipid metabolism during fasting, hypoglycemia, and exercise. *Front Endocrinol*. 2015;6:61.

24. Foong YC, Aitken D, Winzenberg T, Otahal P, Scott D, Jones G. The association between physical activity and reduced body fat lessens with age - results from a cross-sectional study in community-dwelling older adults. *Exp Gerontol*. 2014;55:107–12.

25. Harris TJ, Owen CG, Victor CR, et al. A comparison of questionnaire, accelerometer, and pedometer: measures in older people. *Med Sci Sport Exerc*. 2009;41:1392–402.

26. Atienza AA, Moser RP, Perna F, et al. Self-reported and objectively measured activity related to biomarkers using NHANES. *Med Sci Sport Exerc*. 2011;43:815–21.

27. Sudlow C, Gallacher J, Allen N, et al. UK Biobank: An Open Access Resource for Identifying the Causes of a Wide Range of Complex Diseases of Middle and Old Age. *PLoS Med*. 2015 Mar;12(3):e1001779.

28. Kim Y, White T, Wijndaele K, et al. Adiposity and grip strength as long-term predictors of objectively measured physical activity in 93 015 adults: the UK Biobank study. *Int J Obes*. 2017;41:1361–8.

For peer review only

For peer review only - http://bmjopen.bmj.com/site/about/guidelines.xhtml

29. Willetts M, Hollowell S, Aslett L, et al. Statistical machine learning of sleep and physical activity phenotypes from sensor data in 96,220 UK Biobank participants. *Sci Rep*. 2018;8(1):7961.

30. Foong YC, Chherawala N, Aitken D, et al. Accelerometer-determined physical activity, muscle mass, and leg strength in community-dwelling older adults. *J Cachexia Sarcopenia Muscle*. 2016 Jun;7(3):275–83.

For peer review only

Figure legends

Figure 1. Association of self-reported and accelerometer-measured physical activity with adiposity variables in UK Biobank

Association of A) accelerometer-measured and B) self-reported physical activity with BMI

Association of C) accelerometer-measured and D) self-reported physical activity with body fat percent

Association of E) accelerometer-measured and F) self-reported physical activity with waist circumference

Physical activity was grouped into tenths, separately in men and women.

Adjusted geometric means (from linear regression models) for BMI, body fat percent, and waist circumference are plotted against the median value within each tenth of self-reported or accelerometer-measured physical activity. Adjusted geometric means are represented by squares for men and triangles for women.

These analyses are stratified by age at recruitment, region of recruitment, and socioeconomic status, and are adjusted for educational qualifications, employment status, smoking status, and alcohol intake frequency. Analyses in women are additionally adjusted for parity and hormone replacement therapy use.

The figure shows point estimates and 95% confidence intervals.

Abbreviations: BMI, body mass index; MET, metabolic equivalent

Figure 2. Association of accelerometer-measured physical activity with body fat percent measured by impedance and DXA in UK Biobank A) men (n=1,185) and B) women (n=1,272)

For peer review only - http://bmjopen.bmj.com/site/about/guidelines.xhtml

For peer review only

Physical activity was grouped into fifths, separately in men and women.

Adjusted geometric means (from linear regression models) for body fat percent are plotted against the median value within each fifth of accelerometer-measured physical activity. Adjusted geometric means are represented by diamonds for body fat percent measured by impedance and circles for body fat percent measured by DXA.

These analyses are restricted to participants with measures of body fat percent by both impedance and DXA. Analyses are stratified by age at recruitment, region of recruitment, and socioeconomic status, and are adjusted for educational qualifications, employment status, smoking status, and alcohol intake frequency. Analyses in women are additionally adjusted for parity and hormone replacement therapy use.

The figure shows point estimates and 95% confidence intervals.

Abbreviations: DXA, dual-energy X-ray absorptiometry

Figure 3. Association of self-reported and accelerometer-measured physical activity with adiposity variables by age group in UK Biobank men

Association of physical activity measured by A) accelerometer and B) self-reported questionnaire with BMI.

Association of physical activity measured by C) accelerometer and D) self-reported questionnaire with body fat percent.

Physical activity was grouped into tenths.

Adjusted geometric means (from linear regression models) for BMI and body fat percent are plotted against the median value within each tenth of self-reported or accelerometer-

For peer review only - http://bmjopen.bmj.com/site/about/guidelines.xhtml

measured physical activity. Adjusted geometric means are represented by diamonds for those under age 55 and squares for those ages 55 or older.

These analyses are stratified by age at recruitment, region of recruitment, and socioeconomic status, and are adjusted for educational qualifications, employment status, smoking status, and alcohol intake frequency.

The figure shows point estimates and 95% confidence intervals.

Abbreviations: BMI, body mass index; MET, metabolic equivalent

Figure 4. Association of self-reported and accelerometer-measured physical activity with adiposity variables by age group in UK Biobank women

Association of physical activity measured by A) accelerometer and B) self-reported questionnaire with BMI.

Association of physical activity measured by C) accelerometer and D) self-reported questionnaire with body fat percent.

Physical activity was grouped into tenths.

Adjusted geometric means (from linear regression models) for BMI and body fat percent are plotted against the median value within each tenth of self-reported or accelerometer-measured physical activity. Adjusted geometric means are represented by diamonds for those under age 55 and squares for those ages 55 or older.

These analyses are stratified by age at recruitment, region of recruitment, and socioeconomic status, and are adjusted for educational qualifications, employment status, smoking status, and alcohol intake frequency, parity, and hormone replacement therapy use.

For peer review only - http://bmjopen.bmj.com/site/about/guidelines.xhtml

For peer review only

The figure shows point estimates and 95% confidence intervals.

Abbreviations: BMI, body mass index; MET, metabolic equivalent

Supplementary Figure 1. Flowchart illustrating the application of exclusion criteria for the current study in UK Biobank

For peer review only

For peer review only - http://bmjopen.bmj.com/site/about/guidelines.xhtml

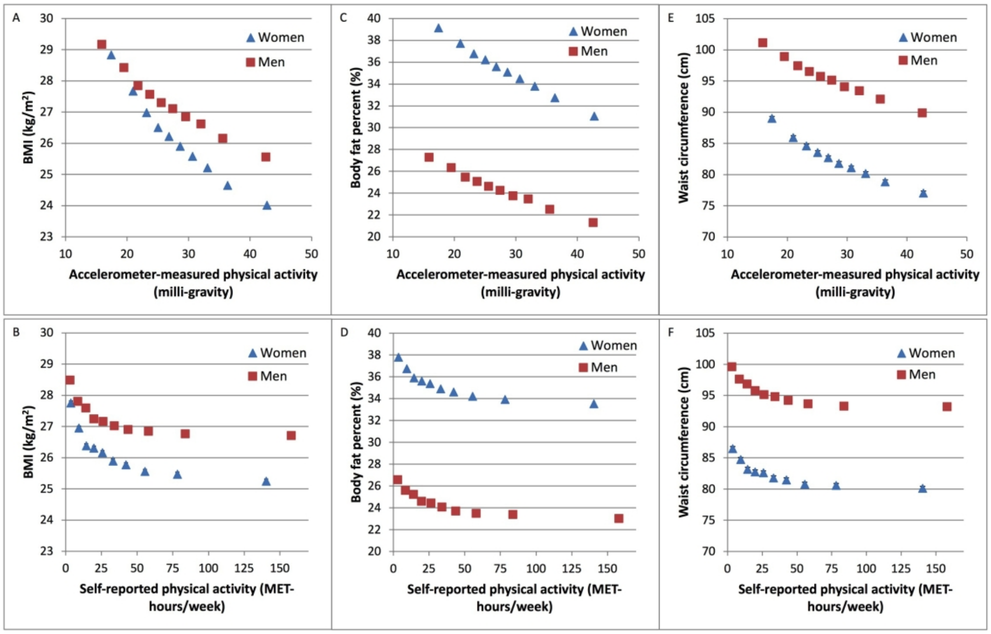

Figure 1. Association of self-reported and accelerometer-measured physical activity with adiposity variables in UK BiobankAssociation of A) accelerometer-measured and B) self-reported physical activity with BMIAssociation of C) accelerometer-measured and D) self-reported physical activity with body fat percentAssociation of E) accelerometer-measured and F) self-reported physical activity with waist circumferencePhysical activity was grouped into tenths, separately in men and women.Adjusted geometric means (from linear regression models) for BMI, body fat percent, and waist circumference are plotted against the median value within each tenth of self-reported or accelerometer-measured physical activity. Adjusted geometric means are represented by squares for men and triangles for women. These analyses are stratified by age at recruitment, region of recruitment, and socioeconomic status, and are adjusted for educational qualifications, employment status, smoking status, and alcohol intake frequency. Analyses in women are additionally adjusted for parity and hormone replacement therapy use. The figure shows point estimates and 95% confidence intervals.Abbreviations: BMI, body mass index; MET, metabolic equivalent

196x126mm (300 x 300 DPI)

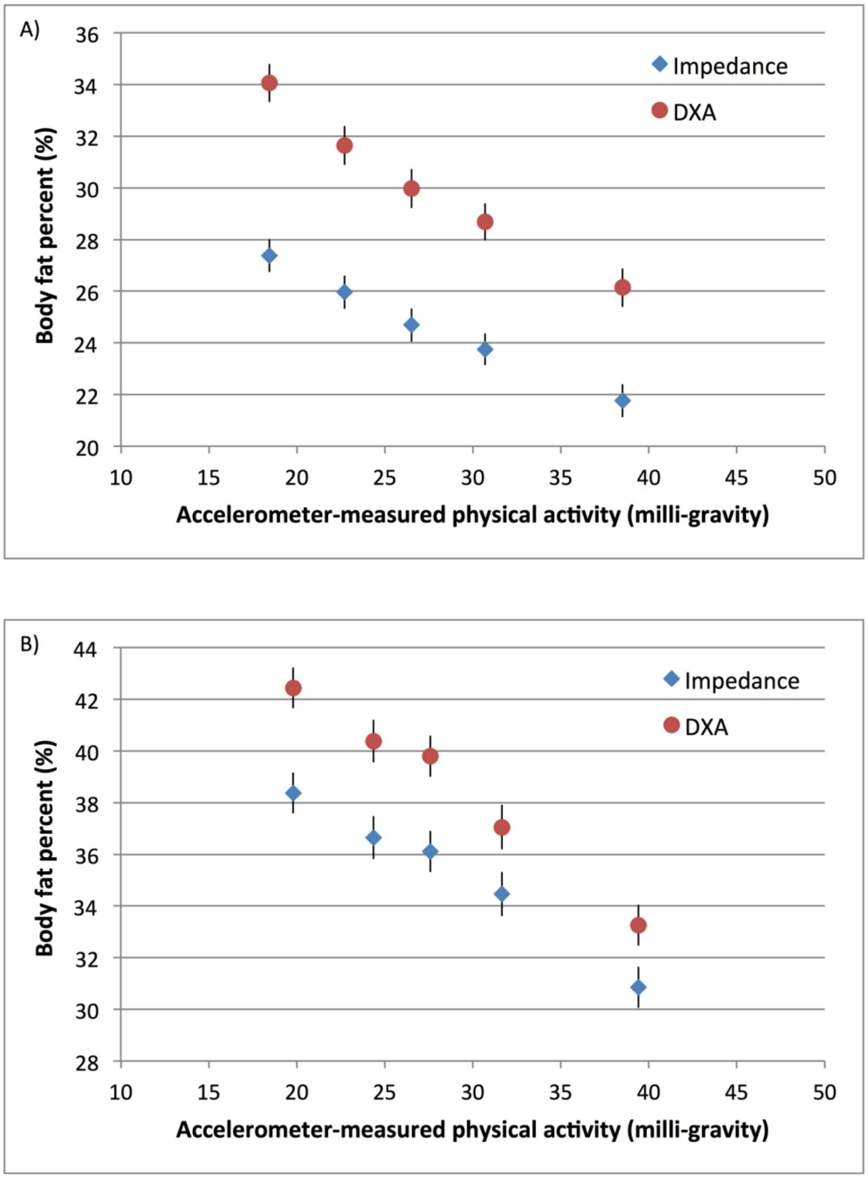

Figure 2. Association of accelerometer-measured physical activity with body fat percent measured by impedance and DXA in UK Biobank A) men (n=1,185) and B) women (n=1,272)Physical activity was grouped into fifths, separately in men and women.Adjusted geometric means (from linear regression models) for body fat percent are plotted against the median value within each fifth of accelerometer-measured physical activity. Adjusted geometric means are represented by diamonds for body fat percent measured by impedance and circles for body fat percent measured by DXA.These analyses are restricted to participants with measures of body fat percent by both impedance and DXA. Analyses are stratified by age at recruitment, region of recruitment, and socioeconomic status, and are adjusted for educational qualifications, employment status, smoking status, and alcohol intake frequency. Analyses in women are additionally adjusted for parity and hormone replacement therapy use. The figure shows point estimates and 95% confidence intervals.Abbreviations: DXA, dual-energy X-ray absorptiometry

150x198mm (300 x 300 DPI)

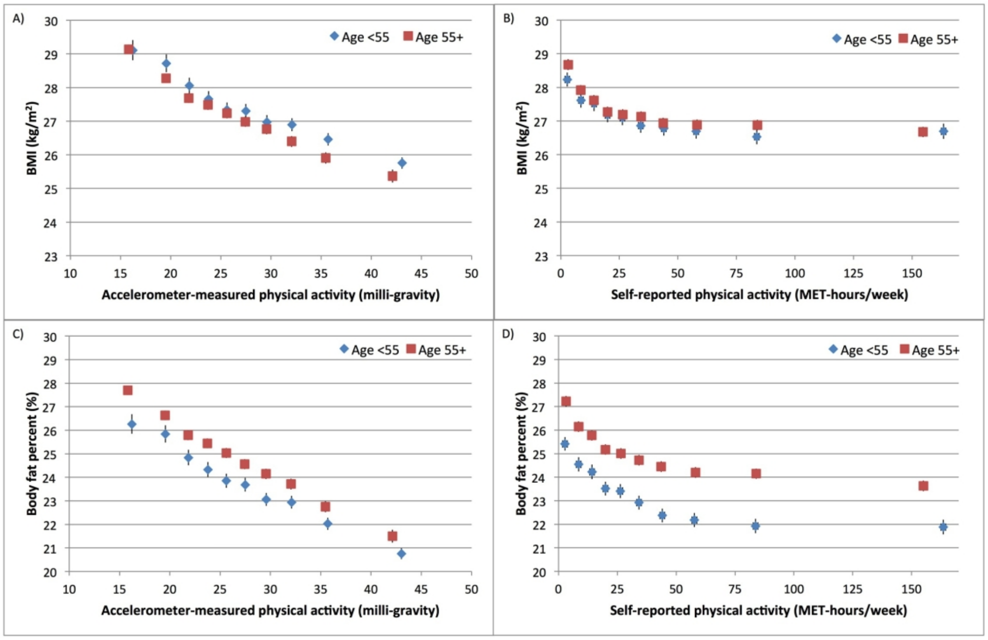

Figure 3. Association of self-reported and accelerometer-measured physical activity with adiposity variables by age group in UK Biobank menAssociation of physical activity measured by A) accelerometer and B) self-reported questionnaire with BMI.Association of physical activity measured by C) accelerometer and D) self-reported questionnaire with body fat percent.Physical activity was grouped into tenths.Adjusted geometric means (from linear regression models) for BMI and body fat percent are plotted against the median value within each tenth of self-reported or accelerometer-measured physical activity. Adjusted geometric means are represented by diamonds for those under age 55 and squares for those ages 55 or older. These analyses are stratified by age at recruitment, region of recruitment, and socioeconomic status, and are adjusted for educational qualifications, employment status, smoking status, and alcohol intake frequency. The figure shows point estimates and 95% confidence intervals.Abbreviations: BMI, body mass index; MET, metabolic equivalent

196x128mm (300 x 300 DPI)

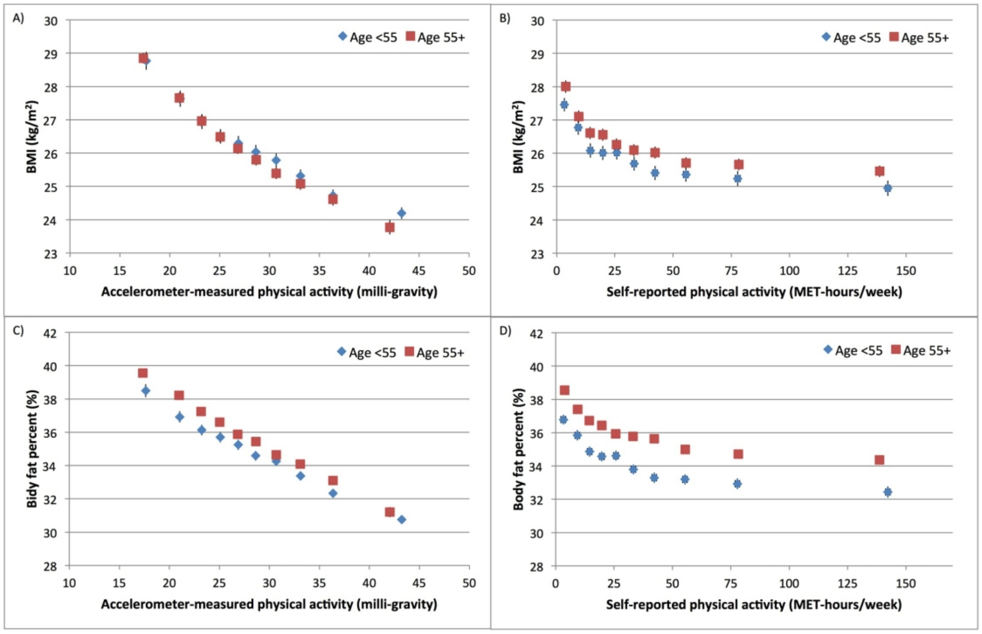

Figure 4. Association of self-reported and accelerometer-measured physical activity with adiposity variables by age group in UK Biobank womenAssociation of physical activity measured by A) accelerometer and B) self-reported questionnaire with BMI.Association of physical activity measured by C) accelerometer and D) self-reported questionnaire with body fat percent.Physical activity was grouped into tenths.Adjusted geometric means (from linear regression models) for BMI and body fat percent are plotted against the median value within each tenth of self-reported or accelerometer-measured physical activity. Adjusted geometric means are represented by diamonds for those under age 55 and squares for those ages 55 or older.These analyses are stratified by age at recruitment, region of recruitment, and socioeconomic status, and are adjusted for educational qualifications, employment status, smoking status, and alcohol intake frequency, parity, and hormone replacement therapy use. The figure shows point estimates and 95% confidence intervals.Abbreviations: BMI, body mass index; MET, metabolic equivalent

195x126mm (300 x 300 DPI)

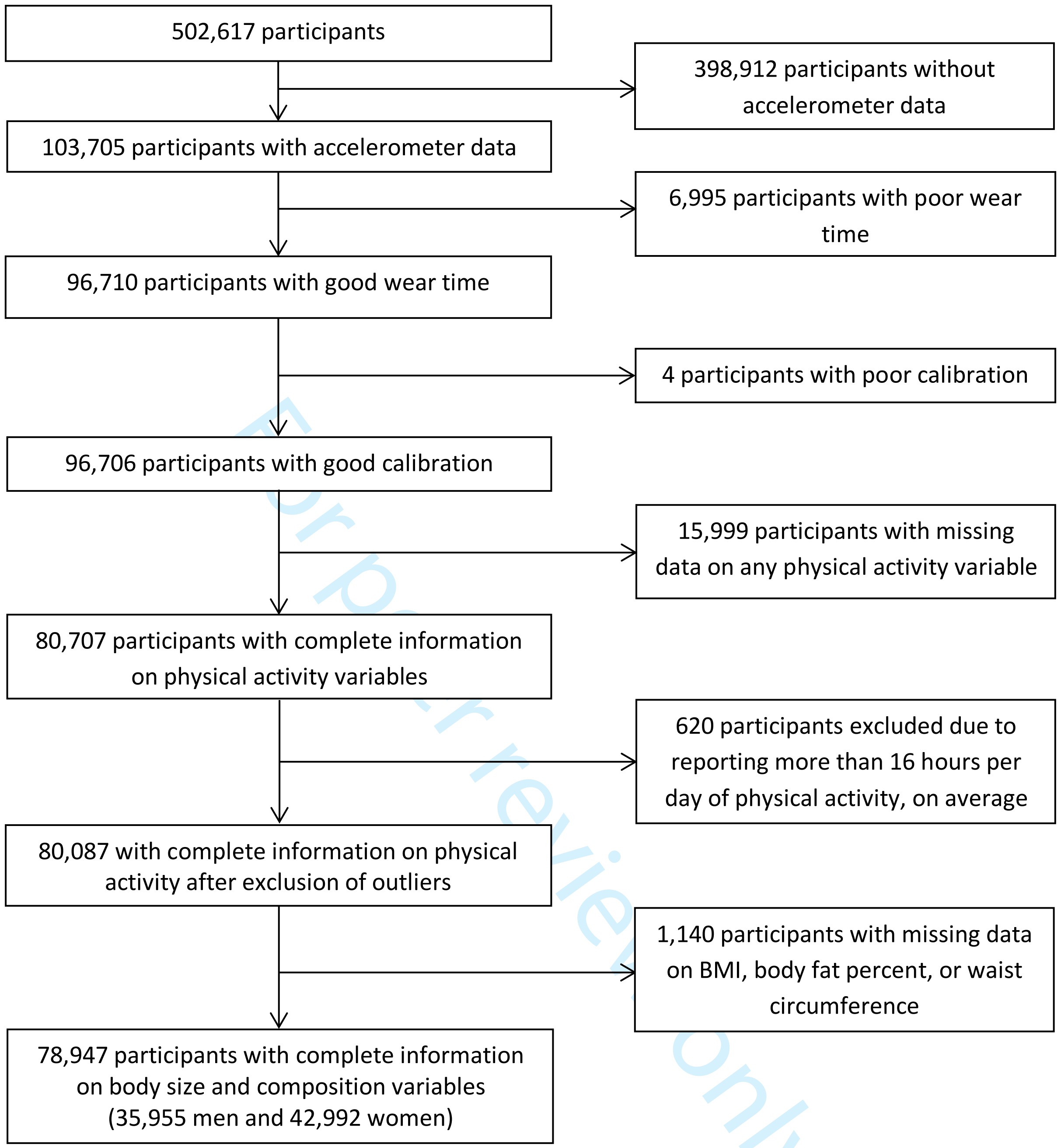
502,617 participants
398,912 participants without accelerometer data
103,705 participants with accelerometer data
6,995 participants with poor wear time
96,710 participants with good wear time
4 participants with poor calibration
96,706 participants with good calibration
15,999 participants with missing data on any physical activity variable
80,707 participants with complete information on physical activity variables
620 participants excluded due to reporting more than 16 hours per day of physical activity, on average
80,087 with complete information on physical activity after exclusion of outliers
1,140 participants with missing data on BMI, body fat percent, or waist circumference
78,947 participants with complete information on body size and composition variables (35,955 men and 42,992 women)

Supplementary Table 1. Physical activity questions from the UK Biobank baseline questionnaire

Question	Potential Responses
In a typical WEEK, on how many days did you walk for at least 10 minutes at a time? (Include walking that you do at work, travelling to and from work, and for sport or leisure)	Number of days, “Do not know”, “Unable to walk”, or “Prefer not to answer”
How many minutes did you usually spend walking on a typical DAY?	Quantity of minutes, “Do not know”, or “Prefer not to answer”
In a typical WEEK, on how many days did you do 10 minutes or more of moderate physical activities like carrying light loads, cycling at normal pace? (Do not include walking)	Number of days, “Do not know”, or “Prefer not to answer”
How many minutes did you usually spend doing moderate activities on a typical DAY?	Quantity of minutes, “Do not know”, or “Prefer not to answer”
In a typical WEEK, how many days did you do 10 minutes or more of vigorous physical activity? (These are activities that make you sweat or breathe hard such as fast cycling, aerobics, heavy lifting)	Number of days, “Do not know”, or “Prefer not to answer”
How many minutes did you usually spend doing vigorous activities on a typical DAY?	Quantity of minutes, “Do not know”, or “Prefer not to answer”

For peer review only

Supplementary Table 2. Spearman correlation between self-reported physical activity and accelerometer-measured physical activity, according to participant characteristics in UK Biobank men

	N Men (%)	Correlation	95% Confidence Interval
**Total**	35,955	0.24	0.23, 0.25
**Age group at recruitment (years)**			
<55 years	13,214 (36.8)	0.31	0.29, 0.32
55+ years	22,741 (63.3)	0.22	0.21, 0.23
**Socioeconomic status, fifths**			
Top fifth	7,584 (21.1)	0.23	0.21, 0.25
Bottom fifth	6,800 (18.9)	0.26	0.23, 0.28
**BMI (kg/m^2)**			
<25	10,590 (29.5)	0.27	0.25, 0.28
25-29.9	17,874 (49.7)	0.21	0.19, 0.22
>30	7,491 (20.8)	0.22	0.80, 0.24
**College or university degree**			
Yes	16,709 (46.5)	0.25	0.24, 0.27
No	19,246 (53.5)	0.24	0.22, 0.25
**Current employment status**			
In paid employment or self-employed	22,942 (63.8)	0.27	0.26, 0.28
Retired	11,361 (31.6)	0.24	0.22, 0.26
Other	1,652 (4.6)	0.30	0.26, 0.34
**Job involves mainly walking/standing**			
Never or rarely	9,825 (42.8)	0.29	0.27, 0.31
Sometimes	7,534 (32.9)	0.24	0.22, 0.26
Usually or Always	5,574 (24.3)	0.19	0.16, 0.21
**Job involves heavy manual work**			
Never, rarely	16,443 (71.7)	0.27	0.26, 0.29
Sometimes	4,160 (18.1)	0.17	0.14, 0.19
Usually or Always	2,335 (10.2)	0.12	0.08, 0.16
**Alcohol intake frequency**			
Weekly or more	29,421 (81.8)	0.23	0.22, 0.24
Less than weekly	6,530 (18.2)	0.28	0.26, 0.30
**Smoking status**			
Never	18,928 (52.6)	0.26	0.24, 0.27
Ever	16,964 (47.2)	0.22	0.21, 0.24
**Long-standing illness or disability**			
No	25,129 (69.9)	0.23	0.22, 0.24
Yes	10,825 (30.1)	0.25	0.23, 0.27

For peer review only

Supplementary Table 3. Spearman correlation between self-reported physical activity and accelerometer-measured physical activity, according to participant characteristics in UK Biobank women

	N Women (%)	Correlation	95% Confidence Interval
**Total**	42,992	0.22	0.21, 0.23
**Age group at recruitment (years)**			
<55 years	18,973 (44.1)	0.26	0.25, 0.28
55+ years	24,019 (55.9)	0.20	0.19, 0.22
**Socioeconomic status, fifths**			
Top fifth	8,401 (19.5)	0.22	0.30, 0.24
Bottom fifth	8,744 (20.3)	0.22	0.30, 0.24
**BMI (kg/m^2)**			
<25	20,255 (47.1)	0.21	0.20, 0.23
25-29.9	15,146 (35.2)	0.18	0.17, 0.20
>30	7,591 (17.7)	0.15	0.13, 0.17
**College or university degree**			
Yes	19,214 (44.7)	0.22	0.21, 0.24
No	23,778 (55.3)	0.22	0.20, 0.23
**Current employment status**			
In paid employment or self-employed	26,693 (62.1)	0.24	0.23, 0.25
Retired	12,710 (29.6)	0.22	0.20, 0.24
Other	3,589 (8.4)	0.30	0.27, 0.33
**Job involves mainly walking/standing**			
Never or rarely	12,191 (45.7)	0.25	0.23, 0.27
Sometimes	7,839 (29.4)	0.21	0.19, 0.23
Usually or Always	6,648 (24.9)	0.18	0.16, 0.20
**Job involves heavy manual work**			
Never, rarely	20,762 (77.8)	0.24	0.22, 0.25
Sometimes	4,353 (16.3)	0.17	0.14, 0.20
Usually or Always	1,567 (5.9)	0.13	0.08, 0.18
**Alcohol intake frequency**			
Weekly or more	29,829 (69.4)	0.22	0.21, 0.23
Less than weekly	13,152 (30.6)	0.21	0.20, 0.23
**Smoking status**			
Never	25,998 (60.5)	0.21	0.20, 0.22
Ever	16,936 (39.4)	0.23	0.22, 0.25
**Long-standing illness or disability**			
No	32,307 (75.2)	0.21	0.20, 0.22
Yes	10,685 (24.9)	0.23	0.21, 0.24

For peer review only

Supplementary Table 4. Association of self-reported and accelerometer-measured physical activity with measures of adiposity

	N	BMI (kg/m^2)	95% CI	Body fat percent	95% CI	Waist circumference (cm)	95% CI
**Men**							
**Questionnaire**							
Bottom tenth (least active)	3,643	0.00 (reference)		0.00 (reference)		0.00 (reference)	
2nd tenth	3,742	-0.69	-0.87, -0.52	-0.99	-1.23, -0.74	-1.97	-2.45, -1.50
3rd tenth	3,410	-0.91	-1.09, -0.73	-1.42	-1.68, -1.17	-2.89	-3.38, -2.41
4th tenth	3,677	-1.22	-1.40, -1.05	-1.97	-2.22, -1.72	-3.81	-4.29, -3.34
5th tenth	3,522	-1.32	-1.50, -1.14	-2.14	-2.39, -1.88	-4.47	-4.95, -3.99
6th tenth	3,584	-1.45	-1.63, -1.27	-2.50	-2.75, -2.24	-4.79	-5.27, -4.31
7th tenth	3,596	-1.59	-1.76, -1.41	-2.88	-3.13, -2.63	-5.41	-5.89, -4.93
8th tenth	3,591	-1.63	-1.81, -1.45	-3.09	-3.34, -2.83	-5.92	-6.40, -5.44
9th tenth	3,595	-1.71	-1.89, 1.53	-3.21	-3.46, -2.95	-6.33	-6.81, -5.85
Top tenth (most active)	3,595	-1.77	-1.95, -1.59	-3.56	-3.81, -3.31	-6.42	-6.90, -5.93
**Accelerometer**							
Bottom tenth (least active)	3,598	0.00 (reference)		0.00 (reference)		0.00 (reference)	
2nd tenth	3,604	-0.77	-0.95, -0.60	-0.99	-1.24, -0.75	-2.23	-2.70, -1.76
3rd tenth	3,592	-1.33	-1.50, -1.15	-1.83	-2.07, -1.58	-3.69	-4.16, -3.22
4th tenth	3,610	-1.60	-1.77, -1.42	-2.20	-2.45, -1.95	-4.59	-5.06, -4.12
5th tenth	3,597	-1.88	-2.06, -1.71	-2.69	-2.94, -2.44	-5.43	-5.90, -4.96
6th tenth	3,585	-2.07	-2.24, -1.89	-3.04	-3.28, -2.79	-6.00	-6.47, -5.53
7th tenth	3,589	-2.32	-2.49, -2.14	-3.52	-3.77, -3.27	-7.01	-7.49, -6.54
8th tenth	3,594	-2.57	-2.75, -2.39	-3.84	-4.09, -3.59	-7.69	-8.17, -7.22
9th tenth	3,591	-3.02	-3.20, -2.84	-4.75	-5.00, -4.50	-9.01	-9.48, -8.53
Top tenth (most active)	3,595	-3.61	-3.79, -3.43	-5.98	-6.24, -5.73	-11.23	-11.72, -10.75

For peer review only

**Women**							
**Questionnaire**							
Bottom tenth (least active)	4,433	0.00 (reference)		0.00 (reference)		0.00 (reference)	
2nd tenth	4,187	-0.81	-1.00, -0.61	-1.05	-1.33, -0.78	-1.77	-2.25, -1.29
3rd tenth	4,278	-1.37	-1.57, -1.18	-1.89	-2.16, -1.61	-3.33	-3.81, -2.86
4th tenth	4,318	-1.44	-1.64, -1.25	-2.16	-2.43, -1.88	-3.74	-4.22, -3.27
5th tenth	4,296	-1.60	-1.79, -1.40	-2.44	-2.72, -2.17	-3.92	-4.39, -3.44
6th tenth	4,308	-1.86	-2.05, -1.66	-2.89	-3.16, -2.61	-4.71	-5.18, -4.23
7th tenth	4,276	-1.98	-2.18, -1.79	-3.18	-3.45, -2.90	-5.02	-5.50, -4.55
8th tenth	4,300	-2.20	-2.39, -2.00	-3.57	-3.85, -3.30	-5.73	-6.20, -5.25
9th tenth	4,305	-2.28	-2.48, -2.09	-3.86	-4.14, -3.59	-5.91	-6.39, -5.44
Top tenth (most active)	4,291	-2.51	-2.70, -2.31	-4.25	-4.52, -3.97	-6.39	-6.87, -5.91
**Accelerometer**							
Bottom tenth (least active)	4,314	0.00 (reference)		0.00 (reference)		0.00 (reference)	
2nd tenth	4,292	-1.14	-1.33, -0.95	-1.43	-1.70, -1.16	-3.02	-3.49, -2.56
3rd tenth	4,293	-1.84	-2.03, -1.65	-2.35	-2.62, -2.09	-4.42	-4.89, -3.95
4th tenth	4,307	-2.31	-2.50, -2.12	-2.90	-3.17-2.63	-5.43	-5.90, -4.97
5th tenth	4,312	-2.60	-2.79, -2.41	-3.52	-3.79, -3.25	-6.27	-6.74, -5.80
6th tenth	4,286	-2.92	-3.11, -2.72	-4.05	-4.32, -3.79	-7.18	-7.65, -6.71
7th tenth	4,292	-3.22	-3.42, -3.04	-4.63	-4.90, -4.36	-7.88	-8.35, -7.41
8th tenth	4,301	-3.60	-3.80, -3.41	-5.34	-5.61, -5.07	-8.80	-9.27, -8.33
9th tenth	4,305	-4.17	-4.36, -3.98	-6.37	-6.64, -6.10	-10.12	-10.59, -9.64
Top tenth (most active)	4,290	-4.80	-4.99, -4.60	-8.06	-8.33, -7.78	-11.92	-12.39, -11.44

Analyses are adjusted for age, socioeconomic status, alcohol intake, smoking status, educational qualifications, and employment status. Analyses are further adjusted for parity and hormone replacement therapy use in women.

Supplementary Table 5. Association of accelerometer-measured physical activity with body fat percent, measured by bioelectrical impedance and dual energy X-ray absorptiometry (DXA) in UK Biobank

Accelerometer-measured physical activity	N	Mean body fat percent	95% CI	Coefficient	95% CI
**Men**		**Measured by impedance**			
Bottom fifth (least active)	237	27.4	26.7, 28.0	0.00 (reference group)	
2nd fifth	231	26.0	25.3, 26.6	-1.29	-2.21, -0.38
3rd fifth	229	24.7	24.0, 25.3	-2.63	-3.55, -1.71
4th fifth	253	23.8	23.1, 24.4	-3.46	-4.36, -2.56
Top fifth (most active)	235	21.8	21.1, 22.4	-5.51	-6.45, -4.58
**Men**		**Measured by DXA**			
Bottom fifth (least active)	237	34.1	33.3, 34.8	0.00 (reference group)	
2nd fifth	231	31.6	30.9, 32.4	-2.38	-3.45, -1.31
3rd fifth	229	30.0	29.2, 30.7	-4.12	-5.20, -3.05
4th fifth	253	28.7	28.0, 29.4	-5.48	-6.54, -4.42
Top fifth (most active)	235	26.1	25.4, 26.9	-8.18	-9.27, -7.08
**Women**		**Measured by impedance**			
Bottom fifth (least active)	270	38.4	37.6, 39.1	0.00 (reference group)	
2nd fifth	244	36.6	35.9, 37.4	-1.46	-2.55, -0.37
3rd fifth	265	36.1	35.4, 36.9	-1.93	-3.01, -0.85
4th fifth	228	34.5	33.7, 35.3	-3.43	-4.55, -2.31
Top fifth (most active)	265	30.8	30.1, 31.6	-6.97	-8.07, -5.87
**Women**		**Measured by DXA**			
Bottom fifth (least active)	270	42.5	41.7, 43.3	0.00 (reference group)	
2nd fifth	244	40.4	39.6, 41.2	-1.87	-3.04, -0.71
3rd fifth	265	39.8	39.0, 40.6	-2.55	-3.70, -1.40
4th fifth	228	37.1	36.2, 37.9	-4.98	-6.17, -3.78
Top fifth (most active)	265	33.3	32.5, 34.1	-8.83	-10.0, -7.65

Analyses are adjusted for age, socioeconomic status, alcohol intake, smoking status, educational qualifications, and employment status. Analyses are further adjusted for parity and hormone replacement therapy use in women.

For peer review only

STROBE Statement—Checklist of items that should be included in reports of ***cross-sectional studies***

	Item No	Recommendation
**Title and abstract**	1	☑ (*a*) Indicate the study's design with a commonly used term in the title or the abstract pages 1-2
		☑ (*b*) Provide in the abstract an informative and balanced summary of what was done and what was found pages 2-3
**Introduction**		
Background/rationale	2	☑ Explain the scientific background and rationale for the investigation being reported page 4
Objectives	3	☑ State specific objectives, including any prespecified hypotheses page 4
**Methods**		
Study design	4	☑ Present key elements of study design early in the paper pages 5-9
Setting	5	☑ Describe the setting, locations, and relevant dates, including periods of recruitment, exposure, follow-up, and data collection pages 5-7
Participants	6	☑ (*a*) Give the eligibility criteria, and the sources and methods of selection of participants pages 5-6
Variables	7	☑ Clearly define all outcomes, exposures, predictors, potential confounders, and effect modifiers. Give diagnostic criteria, if applicable pages 6-8
Data sources/ measurement	8*	☑ For each variable of interest, give sources of data and details of methods of assessment (measurement). Describe comparability of assessment methods if there is more than one group pages 6-7
Bias	9	☑ Describe any efforts to address potential sources of bias page 4
Study size	10	☑ Explain how the study size was arrived at pages 5-6
Quantitative variables	11	☑ Explain how quantitative variables were handled in the analyses. If applicable, describe which groupings were chosen and why pages 6-8
Statistical methods	12	☑ (*a*) Describe all statistical methods, including those used to control for confounding pages 7-9
		☑ (*b*) Describe any methods used to examine subgroups and interactions pages 8-9
		☑ (*c*) Explain how missing data were addressed pages 8-9
		N/A (*d*) If applicable, describe analytical methods taking account of sampling strategy
		☑ (*e*) Describe any sensitivity analyses pages 8-9
**Results**		
Participants	13*	☑ (a) Report numbers of individuals at each stage of study—eg numbers potentially eligible, examined for eligibility, confirmed eligible, included in the study, completing follow-up, and analysed page 9 and Supplementary Figure 1
		☑ (b) Give reasons for non-participation at each stage Supplementary Figure 1
		☑ (c) Consider use of a flow diagram Supplementary Figure 1
Descriptive data	14*	☑ (a) Give characteristics of study participants (eg demographic, clinical, social) and information on exposures and potential confounders pages 9-10
		☑ (b) Indicate number of participants with missing data for each variable of interest pages 5-6, Supplementary Figure 1
Outcome data	15*	☑ Report numbers of outcome events or summary measures pages 10-12, figures
Main results	16	☑ (*a*) Give unadjusted estimates and, if applicable, confounder-adjusted estimates and their precision (eg, 95% confidence interval). Make clear which confounders were adjusted for and why they were included page 8, figures, supplementary tables,

For peer review only

		22-24 (figure legends)
		☑ (*b*) Report category boundaries when continuous variables were categorized page 9
		N/A (*c*) If relevant, consider translating estimates of relative risk into absolute risk for a meaningful time period
Other analyses	17	☑ Report other analyses done—eg analyses of subgroups and interactions, and sensitivity analyses pages 11-12
**Discussion**		
Key results	18	☑ Summarise key results with reference to study objectives pages 12-13
Limitations	19	☑ Discuss limitations of the study, taking into account sources of potential bias or imprecision. Discuss both direction and magnitude of any potential bias pages 15-16
Interpretation	20	☑ Give a cautious overall interpretation of results considering objectives, limitations, multiplicity of analyses, results from similar studies, and other relevant evidence pages 13-16
Generalisability	21	☑ Discuss the generalisability (external validity) of the study results page 16
**Other information**		
Funding	22	☑ Give the source of funding and the role of the funders for the present study and, if applicable, for the original study on which the present article is based page 16

*Give information separately for exposed and unexposed groups.

Note: An Explanation and Elaboration article discusses each checklist item and gives methodological background and published examples of transparent reporting. The STROBE checklist is best used in conjunction with this article (freely available on the Web sites of PLoS Medicine at http://www.plosmedicine.org/, Annals of Internal Medicine at http://www.annals.org/, and Epidemiology at http://www.epidem.com/). Information on the STROBE Initiative is available at www.strobe-statement.org.

For peer review only
